# Optimization of 2-Amino-4,6-diarylpyrimidine-5-carbonitriles as Potent and Selective A_1_ Antagonists

**DOI:** 10.1021/acs.jmedchem.1c01636

**Published:** 2022-01-22

**Authors:** Cristina Val, Carlos Rodríguez-García, Rubén Prieto-Díaz, Abel Crespo, Jhonny Azuaje, Carlos Carbajales, Maria Majellaro, Alejandro Díaz-Holguín, José M. Brea, Maria Isabel Loza, Claudia Gioé-Gallo, Marialessandra Contino, Angela Stefanachi, Xerardo García-Mera, Juan C. Estévez, Hugo Gutiérrez-de-Terán, Eddy Sotelo

**Affiliations:** † Centro Singular de Investigación en Química Biolóxica e Materiais Moleculares (CiQUS), Universidade de Santiago de Compostela, Santiago de Compostela 15782, Spain; ‡ Departamento de Química Orgánica, Universidade de Santiago de Compostela, Santiago de Compostela 15782, Spain; § Centro Singular de Investigación en Medicina Molecular y Enfermedades Crónicas (CiMUS), Universidade de Santiago de Compostela, Santiago de Compostela 15782, Spain; ∥ Dipartimento di Farmacia-Scienze del Farmaco, Università degli Studi di Bari ALDO MORO, via Orabona 4, Bari 70125, Italy; ⊥ Department of Cell and Molecular Biology, Uppsala University, Uppsala 75124, Sweden

## Abstract

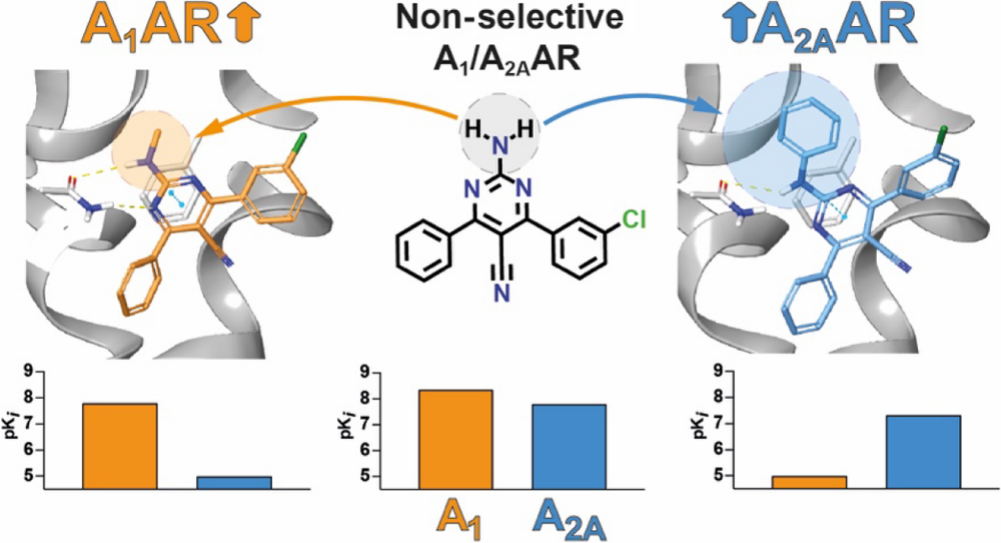

We herein document a large collection of 108 2-amino-4,6-disubstituted-pyrimidine derivatives as potent, structurally simple, and highly selective A_1_AR ligands. The most attractive ligands were confirmed as antagonists of the canonical cyclic adenosine monophosphate pathway, and some pharmacokinetic parameters were preliminarilly evaluated. The library, built through a reliable and efficient three-component reaction, comprehensively explored the chemical space allowing the identification of the most prominent features of the structure–activity and structure–selectivity relationships around this scaffold. These included the influence on the selectivity profile of the aromatic residues at positions R^4^ and R^6^ of the pyrimidine core but most importantly the prominent role to the unprecedented A_1_AR selectivity profile exerted by the methyl group introduced at the exocyclic amino group. The structure–activity relationship trends on both A_1_ and A_2A_ARs were conveniently interpreted with rigorous free energy perturbation simulations, which started from the receptor-driven docking model that guided the design of these series.

## Introduction

The endogenous nucleoside adenosine is essential for the proper functioning of every cell in mammalian species.^1,2^ Adenosine is produced intra- and extracellularly (both in the brain and in the periphery) under diverse physiological and pathophysiological conditions, and its effects are mediated through activation of four membrane adenosine receptors (ARs), namely, A_1_AR, A_2A_AR, A_2B_AR, and A_3_AR.^1,3^ ARs are expressed ubiquitously and play critical roles in the regulation of cardiac muscles,^4^ neuronal function,^5,6^ pain,^2,3^ and sleep.^3,7,8^ In addition to its cytoprotective mission, there are instances in which a chronic overproduction of adenosine becomes pathological (e.g., cancer, diabetes, colitis, fibrosis, hepatic steatosis, or asthma).^2,3,9,10^ A large body of evidence supports that the regulation of the adenosinergic signaling pathways by compounds that modulate the different ARs [e.g., (full or partial) agonists, antagonists/inverse agonists, and allosteric modulators] constitutes innovative approaches to address challenging medical needs.^11−14^

Since its early discovery and cloning,^15^ the A_1_AR has been considered an attractive target for therapeutic intervention.^16^ It is highly abundant not only in the central nervous system (cortex, hippocampus, cerebellum, astrocytes, oligodendrocytes, and microglia) but also in peripheral tissues (heart, kidney, airway smooth muscles, skeletal muscles, liver, or pancreas), thus emphasizing its pivotal role in a diverse physiological process.^3,14^ The A_1_AR is implicated not only in the central excitatory system, participating within the development of several neurological and neurodegenerative disorders (e.g., epilepsy, depression, or Parkinson’s), but also in cognitive functions.^3,7^ Recent evidence supports that the A_1_AR blockade increases extracellular levels of acetylcholine, a neurotransmitter highly decreased in Alzheimer’s disease.^17^ On the other hand, peripheral A_1_AR has been targeted in the search of novel drugs for hypertension, heart failure, allergy, or asthma.^18^ In particular, A_1_AR antagonists have been proposed as effective potassium-sparing diuretic agents with kidney protecting properties.^19^ Currently, the only A_1_AR antagonist in clinical studies is PBF-680 (structure not disclosed), which is undergoing phase **II** as a peripheral selective oral treatment for respiratory diseases (asthma and COPD).^20,21^

The increasing availability of crystallographic and cryo-EM AR structures, complemented with homology models and decades of site-directed mutagenesis studies,^22^ allowed us to improve our molecular understanding of ligand recognition and receptor signaling within the AR family, thus providing solid foundations for the rational design of AR modulators.^23,24^ In particular, we now have A_1_AR structures in both the inactive and active states.^25,26^ Moreover, the X-ray crystal structures of both of A_1_AR and A_2A_AR in complex with the A_1_ selective antagonist PSB36 provided structural insight into receptor selectivity,^27^ further explored with computational methods.^28^

**Figure 1:**
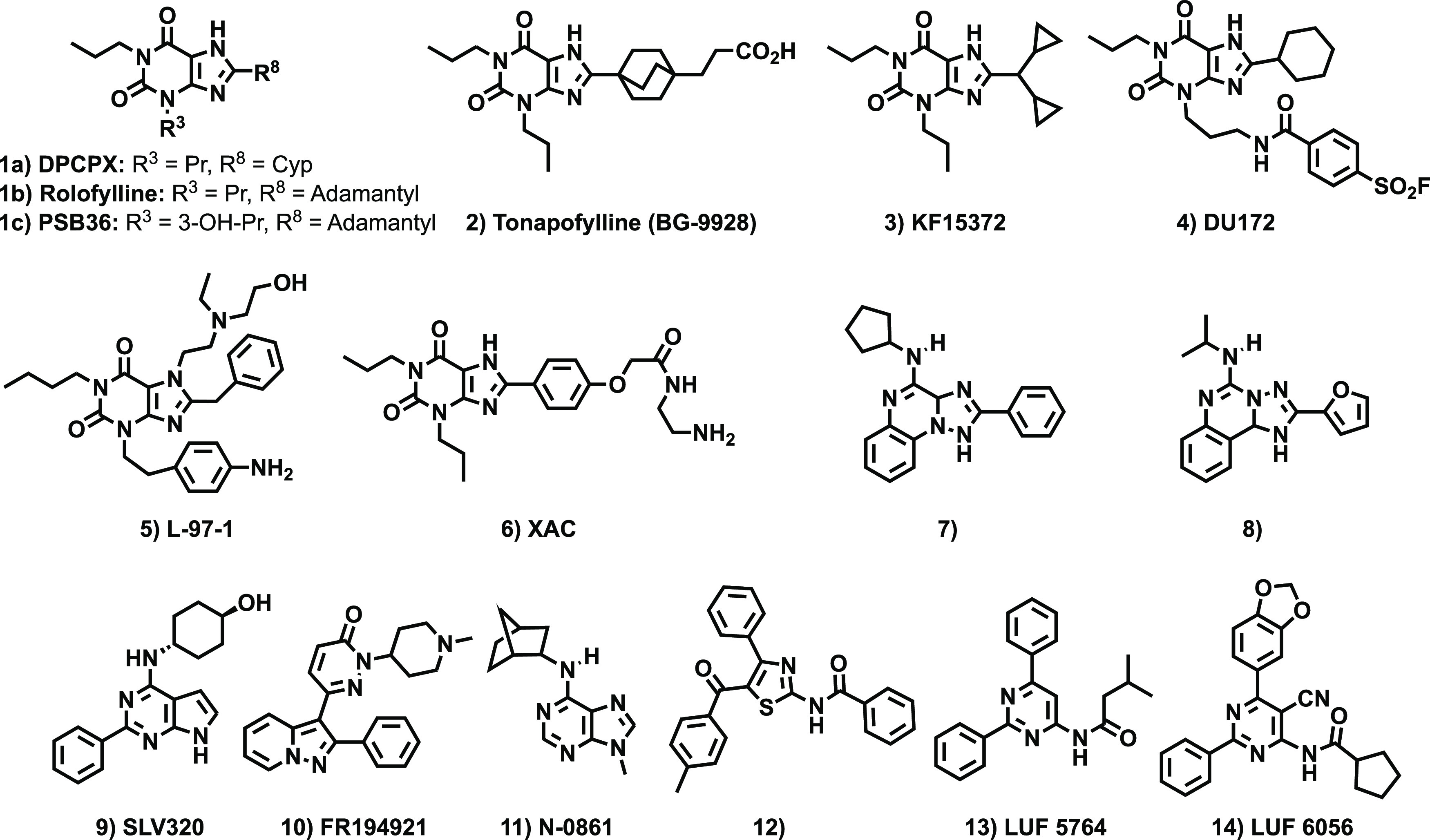
Structure of representative A_1_ adenosine receptor antagonists.^25,29−42^

The therapeutic applications emerging from A_1_AR modulation stimulated the development of several series of small molecule A_1_AR ligands.^18−21^ From these, A_1_AR antagonists can be classified in two structural families: xanthines and non-xanthines (Figure 1). The discovery that naturally occurring alkylxanthines (e.g., caffeine, theophylline, and theobromine) are micromolar (non-selective) AR antagonists inspired extensive pharmacomodulation of the xanthine moiety, thus culminating with the identification of potent and selective A_1_AR, A_2A_AR, and A_2B_AR antagonists. Xanthine-based A_1_AR antagonists generally contain a bulky hydrophobic group at position 8 and alkyl chains at positions 1 and 3 (Figure 1, Cpds **1**–**6**).^25,29−35^ Despite possessing excellent affinity and subtype selectivity, the advancement of xanthine-based A_1_AR antagonists as drug candidates has been hampered by their poor bioavailability and low water solubility, narrow efficacy, and off-target effects.^18−21^ Efforts to identify non-xanthine A_1_AR antagonists mostly focused on bicyclic scaffolds that somehow mimic the adenine core present in the endogenous ligand (adenosine) and, to a lesser extent, tricyclic (Figure 1, Cpds **7** and **8**)^36,37^ and monocyclic systems (Figure 1, Cpds **9**–**11**).^38−40^ However, the high structural homology between the A_1_AR and A_2A_AR, particularly in the orthosteric site, has limited the development of A_1_AR antagonists exhibiting both high affinity and selectivity against the A_2A_AR. Thus, only a few truly selective monocyclic A_1_AR antagonists have been described so far, with representative examples based on the thiazole and pyrimidine cores (Figure 1, Cpds **12**–**14**).^41,42^ It follows that the identification of highly potent and selective structurally simple A_1_AR antagonists remains a challenging goal.

As part of a program aimed at the development of adenosine receptor antagonists, we here report the discovery, optimization, pharmacological profiling, and structure-based SAR of potent, structurally simple, and highly selective non-xanthine A_1_AR antagonists. A large library, consisting of 108 ligands derived of the 2-amino-4,6-disubstitutedpyrimidin-5-carbonitrile chemotype, was obtained by using a novel, succinct, and efficient three-component synthetic strategy. The interpretation of the main structure–activity relationship trends within the series was supported by free energy perturbation (FEP) simulations based on the crystal structure of the human A_1_ receptor. A preliminary exploration of the pharmacokinetic profile of the most attractive ligands identified (**19l**, **19v**, and **19ao**) was carried out by determining its microsomal stability and solubility. Finally, to explore the potential of the designed ligands as CNS agents, we investigated their ability to be substrates of P-glycoprotein (P-gp), the efflux pump present at the blood brain barrier, which represents the first line of defense of the CNS.

## Results and Discussion

### Design

**Figure 2:**
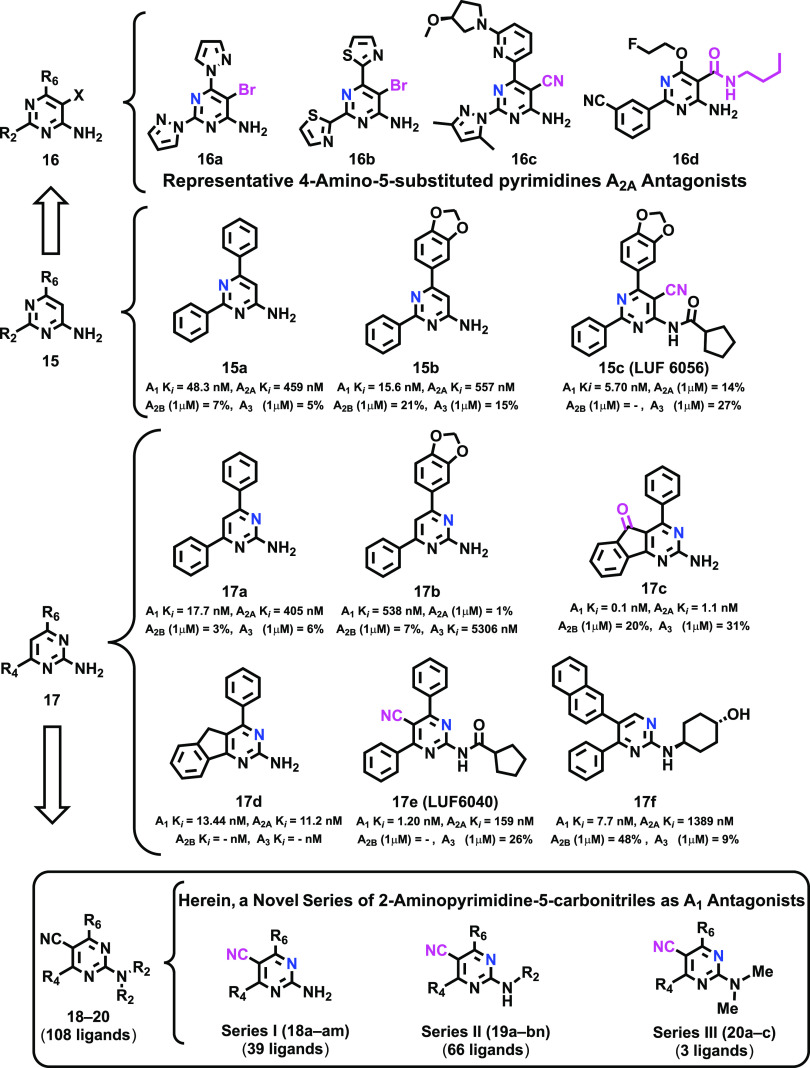
Structure of the model (2- or 4-)-aminopyrimidines (**15**–**17**) and herein documented A_1_ antagonists (**18**–**20**).^42−44^

The design of the 2-amino-4,6-disubstitued-pyriminine-5-carbonitriles (**18**–**20**) was based on the analysis of the adenosinergic profile observed for two regioisomeric series of (2- or 4-)-aminodiarylpyrimidine derivatives (Figure 2, Cpds **15** and **17**), complemented by further inspection of the SAR available for these subsets.^42−44^ (2- or 4-)-Aminodiarylpyrimidine derivatives tend to exhibit a rather intrinsic dual A_1_AR/A_2A_AR antagonistic profile. However, over the last few years, these scaffolds have been explored to develop a novel series of either A_1_AR or A_2A_AR selective ligands. Pharmacomodulation of the 4-aminopyrimidine core successfully afforded a novel series of selective A_2A_AR antagonists, achieved by substitution at position 5 of the heterocycle and adequate decoration of positions 2 and 6 (Figure 2, Cpds **16**). Conversely, this same scaffold provided A_1_AR antagonists by introducing a cyano group at position 5 and transforming the amino group in substituted amides (Figure 2, Cpd **15c**). In a clear contrast, most ligands derived from the 2-aminopyrimidine scaffold retained the dual A_1_AR/A_2A_AR antagonistic profile (Figure 2, Cpds **17a**–**d**), showing that achieving a selective profile for this scaffold is somehow more challenging. Two remarkable exceptions are compounds **17e** and **17f** (Figure 2).^42^ The former was developed by van Veldhoven et al.^42^ by the introduction of analogous substitutions used in their A_1_-selective 4-amidopyrimidine **15c**, while **17f** was obtained by moving the aromatic ring (naphthyl group) to position 5 and the introduction of the cycloalkyl fragment present in SLV320 (Figure 1) in position 2. Although compounds **15c** and **17e** showed high A_1_AR potency and selectivity, most of their congeners could not escape the dual A_2A_AR/A_1_AR profile observed in the early series, thus suggesting that amide formation is not enough to achieve A_1_AR selectivity. The beneficial effects of dual A_1_AR/A_2A_AR antagonism in Parkinson animal models, observed for Cpds **17c**–**e**^43^ (Figure 2), supported prioritization of dual ligands, resulting in a decay of the interest in the identification of selective ligands between these two subtypes of the ARs.

We herein developed a novel series of 2-aminodiarylpyrimidine derivatives (Figure 2, Cpds **18**–**20**), eliciting excellent A_1_AR affinity and selectivity, which distinctively combine chemical decorations inspired by the SAR data discussed above: (i) a cyano group at position 5, (ii) diverse aryl groups at positions 4 and 6, and (iii) free, mono-, or disubstituted amino groups at position 2. The hypothesis behind this design relies on the effect of the cyano group at position 5, which increases the acidity of the exocyclic (substituted) amino group, leading to stronger binding to the ARs by reinforcing the double-hydrogen bond with Asn^6.55^, while R^4^, R^6^, and particularly R^2^ would control the selectivity profile.

### Chemistry

**Scheme 1:**
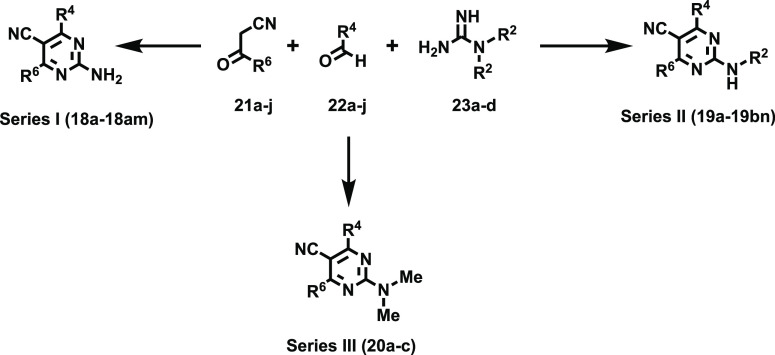
Three-Component Assembly of the Novel 2-Amino-4,6-diaryl-5-carbonitriles (**18**–**20**)

The targeted 2-amino-4,6-disubstitutedpyrimidine-5-carbonitriles (**18**, **19**, and **20**) were assembled following an efficient and convergent three-component transformation (Scheme 1) described by our group.^45^ The Biginelli-inspired preparative method relies on the reaction of α-cyanoketones (**21**), carboxaldehydes (**22**), and guanidines (**23**) in a one-pot sequence that renders **18**–**21** in moderate to excellent yields (45–89%) after purification by either column chromatography or crystallization (isopropanol or ethanol). The three-component transformation includes a sequence involving condensation, nucleophilic addition, cyclization, and spontaneous aromatization of the 2-amino-1,4-dihydropyrimidine-5-carbonitrile intermediate. A collection of structurally diverse starting materials (**21**–**23**) was selected to accomplish an exhaustive exploration of the SAR trends within positions 2, 4, and 6 in the pyrimidine template. Four guanidine precursors (**23a**–**c**) were employed for library synthesis, thus enabling a detailed exploration of the SAR trends in this series. According to the substitution pattern of the amino group (Scheme 1), the 2-amino-4,6-disubstitutedpyrimidine-5-carbonitrile collection was classified in three subsets (**18**, **19**, and **20**) containing 39, 66, and 3 derivatives, respectively.

### Biological Evaluation

The adenosinergic profile (affinity and selectivity) of the 108 synthesized derivatives of the 2-aminopyrimidine-5-carbonitrile scaffold was evaluated *in vitro* using radioligand binding assays at the four human AR subtypes.^46−49^Tables 1–3 contain the binding data of the three novel series herein reported. In brief, human adenosine receptors were expressed in transfected CHO (A_1_AR), HeLa (A_2A_AR and A_3_AR), and HEK-293 (A_2B_AR) cells. [^3^H]-1,3-Dipropyl-8-cyclopentylxanthine ([^3^H]DPCPX) for both A_1_AR and A_2B_AR, [^3^H]4-(2-[7-amino-2-(2-furyl)[1,2,4]triazolo[2,3-*a*][1,3,5]triazin-5-ylamino]ethyl)phenol ([^3^H]ZM241385) for A_2A_AR, and [^3^H]NECA for A_3_AR were employed as radioligands in binding assays. The biological data are expressed as *K_i_* (nM, *n* = 3) or as percentage inhibition of specific binding at 1 μM (*n* = 2, average) for those compounds that did not fully displace specific radioligand binding. *K_i_* values were obtained by fitting the data with non-linear regression using Prism 2.1 software (GraphPad, San Diego, CA). For comparative purposes, the binding affinities obtained for three representative AR ligands (**XAC**, **ZM241385**, and **DPCPX**), using the binding protocols herein employed, are included in Tables 1–3. The whole set of ligands (**18**–**20**) was in silico evaluated, using the PAINS filter in the RDkit,^50^ to rule out these ligands being promiscuous pan-assay interference compounds (PAINS).

**Table 1:**
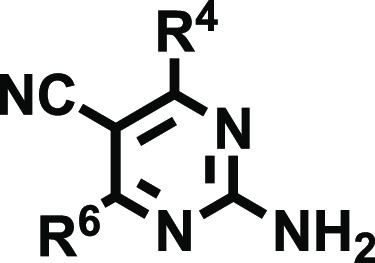
Structure and Affinity Binding Data for Series **I**: 2-Amino-4,6-diaryl-5-carbonitriles **18a**–**18am** at the Human ARs

			*K_i_* (nM) or % at 1 μM			
Cpd	R^4^	R^6^	*h*A_1_^a^	*h*A_2A_^b^	*h*A_2B_^c^	*h*A_3_^d^
**18a**(27)	Ph	Ph	4.42 ± 0.16	18.6 ± 3.4	33%	1%
**18b**	2-F-Ph	Ph	6.44 ± 1.25	17.6 ± 2.8	34%	8%
**18c**	2-Cl-Ph	Ph	5%	1%	9%	1%
**18d**	2-MeO-Ph	Ph	13.1 ± 3.2	17.7 ± 2.1	14%	8%
**18e**	3-F-Ph	Ph	13.4 ± 4.1	19.5 ± 3.3	186 ± 11	2%
**18f**	3-Cl-Ph	Ph	4.25 ± 1.10	15.5 ± 2.4	177 ± 13	1%
**18g**	3-MeO-Ph	Ph	2.08 ± 0.16	6.91 ± 1.52	31.2 ± 6.3	12%
**18h**	3-OH-Ph	Ph	1.49 ± 0.43	10.2 ± 3.52	50.1 ± 8.2	9%
**18i**	3-CN-Ph	Ph	6.49 ± 1.14	84.1 ± 11.7	19%	1%
**18j**	4-F-Ph	Ph	4.19 ± 1.16	16.3 ± 2.9	7%	10%
**18k**	4-Br-Ph	Ph	9.19 ± 2.81	21.9 ± 5.0	8%	2%
**18l**	4-MeO-Ph	Ph	7.16 ± 2.03	46.0 ± 3.2	8%	11%
**18m**	4-OH-Ph	Ph	4.14 ± 0.55	26.5 ± 1.6	14%	18%
**18n**	4-Me-Ph	Ph	5.38 ± 1.36	8.93 ± 0.12	28%	16%
**18o**	2,4-F-Ph	Ph	4.00 ± 1.82	15.5 ± 2.21	7%	12%
**18p**	2,4-Cl-Ph	Ph	35%	29%	2%	7%
**18q**	2,4-MeO-Ph	Ph	3.98 ± 0.74	10.2 ± 1.83	13%	1%
**18r**	3,5-F-Ph	Ph	27%	24%	1%	1%
**18s**	3,5-Cl-Ph	Ph	15.9 ± 2.11	95.4 ± 16.5	1%	10%
**18t**	3,5-MeO-Ph	Ph	2.58 ± 0.67	1.73 ± 0.33	45.1 ± 3.7	3%
**18u**	3,4-OCH_2_O-Ph	Ph	1.75 ± 0.31	10.2 ± 1.09	43.3 ± 4.2	12%
**18v**	3,4,5-MeO-Ph	Ph	2.58 ± 0.05	0.95 ± 0.07	3%	13%
**18w**	2,4,6-F-Ph	Ph	2%	2%	2%	19%
**18x**	2-furyl	Ph	9.70 ± 1.20	10.1 ± 3.7	21.8 ± 2.7	9%
**18y**	2-thienyl	Ph	17.3 ± 4.4	24.9 ± 4.6	40.6 ± 3.7	41%
**18z**	3-furyl	Ph	18.7 ± 3.4	52.1 ± 5.1	37%	3%
**18aa**	3-thienyl	Ph	5.25 ± 2.16	20.6 ± 2.7	40%	1%
**18ab**	4-pyridyl	Ph	216 ± 23	676 ± 27	1%	12%
**18ac**	3-pyridyl	Ph	19.6 ± 1.47	36.4 ± 5.7	20%	1%
**18ad**	cPent	Ph	10%	12%	1%	19%
**18ae**	cHex	Ph	20%	10%	5%	2%
**18af**	2-naphthyl	Ph	8.27 ± 2.10	5.78 ± 1.16	3%	8%
**18ag**	4-Ph-Ph	Ph	6500 ± 451	16%	1%	1%
**18ah**	3-Cl-Ph	3-Cl-Ph	4.82 ± 0.37	35.3 ± 7.7	73.6 ± 6.8	1%
**18ai**	3-Cl-Ph	3,5-Cl-Ph	7.81 ± 1.43	190 ± 22	9%	2%
**18aj**	3-Cl-Ph	3,4-OCH_2_O-Ph	5.61 ± 0.74	101 ± 15	386 ± 15	26%
**18ak**	4-F-Ph	3,4-OCH_2_O-Ph	12.5 ± 2.3	61.0 ± 4.9	2%	22%
**18al**	4-MeO-Ph	4-MeO-Ph	74.3 ± 3.4	2%	1%	9%
**18 am**	2-furyl	4-F-Ph	9.08 ± 1.16	5.72 ± 0.26	13.9 ± 3.7	11%
**XAC**			29.1 ± 7.7	1.0 ± 0.2	141 ± 26	91.9 ± 26.1
**DPCPX**			2.20 ± 0.17	157 ± 38	73.24 ± 5.18	1722 ± 112
**ZM241385**			683 ± 57	1.9 ± 0.27	65.7 ± 5.6	863 ± 37

a Displacement of specific [^3^H]DPCPX binding in human CHO cells expressed as *K_i_* in nM ± SEM (*n* = 3) or percentage displacement of specific binding at a concentration of 1 μM (*n* = 2).

b Displacement of specific [^3^H]ZM241385 binding in human HeLa cells expressed as *K_i_* in nM ± SEM (*n* = 3) or percentage displacement of specific binding at a concentration of 1 μM (*n* = 2).

c Displacement of specific [^3^H]DPCPX binding in human HEK-293 cells expressed as *K_i_* in nM ± SEM (*n* = 3) or percentage displacement of specific binding at a concentration of 1 μM (*n* = 2).

d Displacement of specific [^3^H]NECA binding in human HeLa cells expressed as *K_i_* in nM ± SEM (*n* = 3) or percentage displacement of specific binding at a concentration of 1 μM (*n* = 2).

**Table 2:**
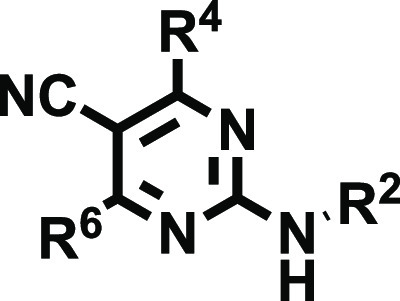
Structure and Affinity Binding Data for Series **II**: 2-Amino-4,6-diaryl-5-carbonitriles **19a**–**19bn** at the Human ARs

				*K_i_* (nM) or % at 1 μM			
Cpd	R^4^	R^6^	R^2^	*h*A_1_^a^	*h*A_2A_^b^	*h*A_2B_^c^	*h*A_3_^d^
**19a**	Ph	Ph	Me	9.14 ± 2.21	711 ± 43	14%	2%
**19b**	Ph	Ph	Et	5.82 ± 1.16	357 ± 21	17%	16%
**19c**	Ph	Ph	Ph	45.6 ± 6.7	55.1 ± 4.3	34%	4%
**19d**	2-F-Ph	Ph	Me	29.5 ± 2.3	15%	47.8 ± 3.8	3%
**19e**	2-F-Ph	Ph	Ph	6.25 ± 1.02	52.5 ± 6.2	107 ± 10	2%
**19f**	2-Cl-Ph	Ph	Me	2%	2%	6%	2%
**19g**	2-Cl-Ph	Ph	Ph	16%	5%	14%	1%
**19h**	2-MeO-Ph	Ph	Me	17%	1%	9%	1%
**19i**	2-MeO-Ph	Ph	Ph	2%	2%	5%	9%
**19j**	3-F-Ph	Ph	Me	28.5 ± 2.7	11%	358 ± 27	1%
**19k**	3-F-Ph	Ph	Ph	1%	12%	9%	9%
**19l ISAM-CV207**	3-Cl-Ph	Ph	Me	15.7 ± 3.6	2%	12%	2%
**19m**	3-Cl-Ph	Ph	Et	5.10 ± 1.8	295 ± 27	33%	2%
**19n ISAM-CV245**	3-Cl-Ph	Ph	Ph	22%	46.3 ± 2.5	12%	15%
**19o**	3-OH-Ph	Ph	Me	2.48 ± 0.71	105 ± 8	29%	9%
**19p**	3-OH-Ph	Ph	Ph	18.3 ± 1.6	71.2 ± 5.3	40%	13%
**19q**	3-MeO-Ph	Ph	Me	13.2 ± 4.1	82.6 ± 4.9	31%	3%
**19r**	3-MeO-Ph	Ph	Ph	95.5 ± 11.7	133 ± 11	15%	9%
**19s**	3-CN-Ph	Ph	Me	2.99 ± 0.71	78.5 ± 6.7	34.6 ± 5.7	1%
**19t**	3-CN-Ph	Ph	Et	2.46 ± 0.18	155 ± 21	14.2 ± 3.8	1%
**19u**	3-CN-Ph	Ph	Ph	18.2 ± 3.1	19%	16.4 ± 2.2	2%
**19v ISAM-CV209**	4-F-Ph	Ph	Me	23.2 ± 1.2	9%	13%	1%
**19w**	4-F-Ph	Ph	Ph	36.3 ± 4.1	10%	51.7 ± 3.1	9%
**19x**	4-Br-Ph	Ph	Me	12%	3%	8%	2%
**19y**	4-Br-Ph	Ph	Ph	5%	8%	1%	43%
**19z**	4-OH-Ph	Ph	Me	44.6 ± 3.2	7%	2%	8%
**19aa**	4-OH-Ph	Ph	Ph	4%	9%	1%	1%
**19ab**	4-MeO-Ph	Ph	Me	57.5 ± 2.7	6%	1%	1%
**19ac**	4-MeO-Ph	Ph	Ph	24%	51.4 ± 3.7	186 ± 15	31%
**19ad**	4-Me-Ph	Ph	Me	28.0 ± 9.3	11%	474 ± 32	7%
**19ae**	4-Me-Ph	Ph	Ph	37.9 ± 5.7	157 ± 16	4%	1%
**19af ISAM-CV216**	2,4-F-Ph	Ph	Me	22.6 ± 7.0	3%	3%	2%
**19ag**	2,4-F-Ph	Ph	Ph	1%	2%	2%	29%
**19ah**	3,5-F-Ph	Ph	Me	16%	1%	15%	2%
**19ai**	3,5-F-Ph	Ph	Ph	1%	1%	2%	1%
**19aj ISAM-CV218**	3,5-Cl-Ph	Ph	Me	27.0 ± 3.6	2%	1%	2%
**19ak**	3,5-Cl-Ph	Ph	Et	135 ± 20	16%	1%	1%
**19al**	3,5-Cl-Ph	Ph	Ph	8%	1%	4%	1%
**19am**	3,5-MeO-Ph	Ph	Me	11.0 ± 0.80	11.5 ± 4.7	60.0 ± 5.1	2%
**19an ISAM-CV247**	3,5-MeO-Ph	Ph	Ph	10%	17.3 ± 1.9	64.0 ± 9.6	1%
**19ao ISAM-CV202**	3,4-OCH_2_O-Ph	Ph	Me	6.11 ± 0.60	14%	16%	17%
**19ap**	3,4-OCH_2_O-Ph	Ph	Et	6.70 ± 0.67	894 ± 42	217 ± 18	1%
**19aq**	3,4-OCH_2_O-Ph	Ph	Ph	11.4 ± 3.7	28.0 ± 9.6	188 ± 23	25%
**19ar**	3,4,5-MeO-Ph	Ph	Me	11.7 ± 3.1	3.63 ± 0.88	1%	15%
**19as**	3,4,5-MeO-Ph	Ph	Ph	76.5 ± 9.1	15.8 ± 3.7	1%	20%
**19at**	2-furyl	Ph	Me	6.66 ± 2.4	401 ± 25	51%	4%
**19au**	2-furyl	Ph	Ph	33.7 ± 7.3	2.15 ± 0.11	14.7 ± 4.9	4%
**19av**	2-thienyl	Ph	Me	18%	25%	9%	1%
**19aw**	2- thienyl	Ph	Ph	42.3 ± 4.6	330 ± 27	1%	3%
**19ax**	3-furyl	Ph	Me	1%	23%	1%	1%
**19ay**	3-furyl	Ph	Ph	2%	368 ± 36	14%	2%
**19az ISAM-CV224**	3-thienyl	Ph	Me	42.8 ± 3.7	26%	51%	1%
**19ba ISAM-CV267**	3-thienyl	Ph	Ph	9%	102 ± 27	17%	22%
**19bb**	4-pyridyl	Ph	Me	56.9 ± 6.7	5%	12%	1%
**19bc**	4-pyridyl	Ph	Ph	30%	17%	18%	12%
**19bd ISAM-CV227**	3-pyridyl	Ph	Me	19.3 ± 7.1	19%	13%	1%
**19be**	3-pyridyl	Ph	Et	11.8 ± 3.5	335 ± 18	1%	2%
**19bf ISAM-CV248**	3-pyridyl	Ph	Ph	23%	27.1 ± 5.7	29.4 ± 4.0	17%
**19bg**	cHex	Ph	Me	2%	1%	1%	11%
**19bh**	cHex	Ph	Ph	1%	1%	6%	3%
**19bi**	3-Cl-Ph	3-Cl-Ph	Me	6%	3%	1%	12%
**19bj**	3-Cl-Ph	3-Cl-Ph	Ph	13%	12%	2%	16%
**19bk**	3,5-Cl-Ph	3-Cl-Ph	Me	10%	1%	4%	11%
**19bl**	3,5-Cl-Ph	3-Cl-Ph	Ph	2%	4%	1%	9%
**19bm**	4-MeO-Ph	4-MeO-Ph	Me	2%	2%	7%	23%
**19bn**	4-MeO-Ph	4-MeO-Ph	Ph	1%	2%	2%	1%
**XAC**				29.1 ± 7.7	1.0 ± 0.2	141 ± 26	91.9 ± 26.1
**DPCPX**				2.20 ± 0.17	157 ± 38	73.24 ± 5.18	1722 ± 112
**ZM241385**				683 ± 57	1.9 ± 0.27	65.7 ± 5.6	863 ± 37

a Displacement of specific [^3^H]DPCPX binding in human CHO cells expressed as *K_i_* in nM ± SEM (*n* = 3) or percentage displacement of specific binding at a concentration of 1 μM (*n* = 2).

b Displacement of specific [^3^H]ZM241385 binding in human HeLa cells expressed as *K_i_* in nM ± SEM (*n* = 3) or percentage displacement of specific binding at a concentration of 1 μM (*n* = 2).

c Displacement of specific [^3^H]DPCPX binding in human HEK-293 cells expressed as *K_i_* in nM ± SEM (*n* = 3) or percentage displacement of specific binding at a concentration of 1 μM (*n* = 2).

d Displacement of specific [^3^H]NECA binding in human HeLa cells expressed as *K_i_* in nM ± SEM (*n* = 3) or percentage displacement of specific binding at a concentration of 1 μM (*n* = 2).

**Table 3:**
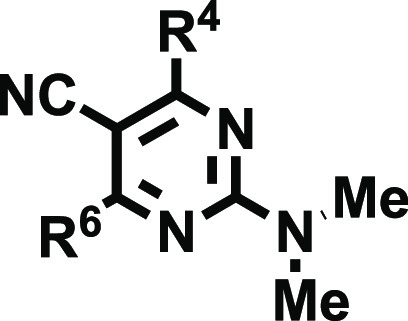
Structure and Affinity Binding Data for Series **III**: 2-Amino-4,6-diaryl-5-carbonitriles **20a**–**c** at the Human ARs

			*K_i_* (nM) or % at 1 μM			
Cpd	R^4^	R^6^	*h*A_1_^a^	*h*A_2A_^b^	*h*A_2B_^c^	*h*A_3_^d^
**20a**	Ph	Ph	8%	1%	3%	9%
**20b**	3-Cl-Ph	Ph	11%	2%	1%	2%
**20c**	4-F-Ph	Ph	12%	2%	4%	1%
**XAC**			29.1 ± 7.7	1.0 ± 0.2	141.0 ± 26.6	91.9 ± 26.1
**DPCPX**			2.20 ± 0.17	157 ± 38	73.24 ± 5.18	1722 ± 112
**ZM241385**			683 ± 57	1.9 ± 0.27	65.7 ± 5.6	863 ± 37

a Displacement of specific [^3^H]DPCPX binding in human CHO cells expressed as *K_i_* in nM ± SEM (*n* = 3) or percentage displacement of specific binding at a concentration of 1 μM (*n* = 2).

b Displacement of specific [^3^H]ZM241385 binding in human HeLa cells expressed as *K_i_* in nM ± SEM (*n* = 3) or percentage displacement of specific binding at a concentration of 1 μM (*n* = 2).

c Displacement of specific [^3^H]DPCPX binding in human HEK-293 cells expressed as *K_i_* in nM ± SEM (*n* = 3) or percentage displacement of specific binding at a concentration of 1 μM (*n* = 2).

d Displacement of specific [^3^H]NECA binding in human HeLa cells expressed as *K_i_* in nM ± SEM (*n* = 3) or percentage displacement of specific binding at a concentration of 1 μM (*n* = 2).

### Functional Experiments and Preliminary ADME Determinations

**Figure 3:**
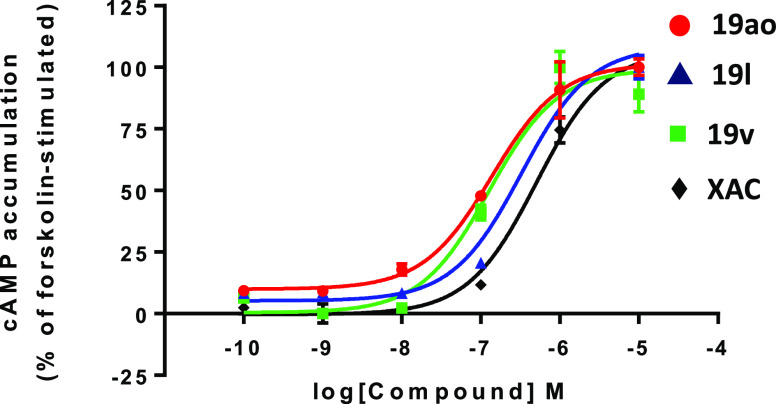
Concentration-response curves of the effect of **19ao**, **19l**, **19v**, and **XAC** on 3 μM forskolin-stimulated cAMP production in the presence of NECA 100 nM.

A representative set of the obtained A_1_AR ligands (**19ao**, **19l**, and **19v**) was evaluated in cAMP assays to determinate their ability to reverse the inhibitory effect of NECA (100 nM) on forskolin-stimulated (3 μM) cAMP production. The log concentration-response curves of cAMP accumulation for selected antagonists to *h*A_1_ARs are presented in Figure 3. These experiments demonstrated that selected compounds (**19ao**, **19l**, and **19v**) and **XAC** reverse the inhibitory effect of NECA on FSK-induced cAMP accumulation, unequivocally validating their antagonism at *h*A_1_ARs. The *K*_B_ values obtained during the functional experiments at *h*A_1_ARs show low nanomolar range data (*K*_B_ = 3.90, 6.21, 9.72, and 14.50 nM). As a complement of these experiments, the functional data of selected compounds (**19ao**, **19l**, and **19v**) was investigated at the other three adenosine receptor subtypes (*h*A_2A_ARs, *h*A_2B_ARs, and *h*A_3_ARs). This study (Supporting Information, Table S2) confirmed that the excellent selectivity profile observed in the binding studies (Tables 1 and 2) is reproduced when evaluating the functional behavior of the new ligands documented here.

Some preliminary ADME experiments were performed to gain insight into the pharmacological profile of representative ligands (**19ao**, **19l**, and **19v**). A solubility assay was performed to evaluate the aqueous solubilities of **19l**, **19v**, and **19ao**. The solubilities, phosphate buffered saline (pH 7.4), were determined to be 75.4, 18.0, and 5.4 μM, respectively. The low solubility observed for **19ao** may be attributed to the lipophilic piperonyl group at R^4^. The stability of selected compounds in human microsomes was also studied (Supporting Information, Table S1). After 60 min of incubation in human microsomes, the remanent ligand ranged from 3.7 to 15%, so further structural optimization should be performed to improve the microsomal stability profile within the series. According to McNaney et al.’s classification,^51^ ligands **19l** and **19ao** can be categorized as intermediate clearance compounds (CL_int_ = 32.41 and 27.78 μL·mg_protein_^–1^·min^–1^, respectively) while **19v** can be considered a high clearance compound (CL_int_ = 50.28 μL·mg_protein_^–1^·min^–1^).

### P-Glycoprotein Interaction Assays

An exploratory cellular-based assay was performed to evaluate the potential of the A_1_AR antagonists here described as active agents at the CNS level. P-Glycoprotein (P-gp)^52^ is an ATPase representing a first line of defense of our brain toward toxins and drugs. P-gp uses the hydrolysis of ATP to efflux drugs out from the brain parenchyma. Therefore, the P-gp interaction profile of drug candidates constitutes an *in vitro* assay informative of the ability of drugs to hit the central targets. For this purpose, we studied the ability of selected compounds (**19l**, **19v**, **19ao**, **19af**, and **19aj**) to compete with the transport of a profluorescent probe, Calcein-AM, that is also a P-gp substrate, in a cell line overexpressing P-gp (MDCK-MDR1 cell line) mimicking the BBB that was measured. Briefly, in MDCK-MDR1 cells, the pro-fluorescent Calcein-AM is not able to enter the cell membrane as effluxed by P-gp; in the presence of an agent able to interact with the pump (as a substrate), Calcein-AM enters the cell membrane and it is hydrolyzed, by the cytosol esterases, to the fluorescent Calcein (responsible for the fluorescence signal).^53−55^ The results of this study are presented in Table 4. As observed, any of the tested compounds showed a significant interaction in MDCK-MDR1 cells with the Calcein-AM transport with respect to the P-gp reference substrate verapamil (EC_50_ = 0.50 μM).^56^ This preliminary (cellular) data suggest that, herein, described ligands should not be effluxed by the pump, thus showing a potential ability to overcome the BBB.

**Table 4:**
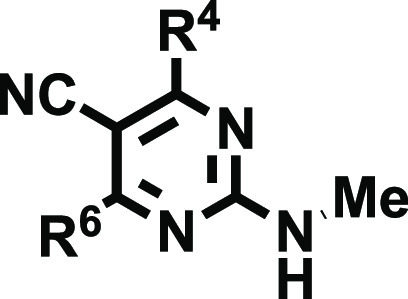
Structure, A_1_AR Binding Data, and Inhibition of the Transport of a P-gp Substrate at 100 μM Representative 2-Amino-4,6-diaryl-5-carbonitriles

cmpd	R^4^	R^6^	*h*A_1_*K_i_* (nM)	Calcein-AM transport inhibition at 100 μM
**19l**	3-Cl-Ph	Ph	15.7 nM	NA
**19ao**	3,4-OCH_2_O-Ph	Ph	6.11 nM	59%^a^
**19v**	4-F-Ph	Ph	23.2 nM	NA
**19af**	2,4-F-Ph	Ph	22.6 nM	44%^a^
**19aj**	3,5-Cl-Ph	Ph	27.0 nM	NA

a Percentage of inhibition at 100 μM. NA = not active.

### Structure–Activity Relationship

Examination of the binding data reveals the identification of eight A_1_AR ligands that combine high affinity (*K_i_* < 50 nM) and outstanding selectivity (>1000-fold; see Table 2, compounds **19l**, **19v**, **19z**, **19af**, **19aj**, **19ao**, **19az**, and **19bd**). Although this project focuses on the identification of A_1_AR antagonists, during the pharmacological screening of the obtained library, we identified three A_2A_AR selective ligands eliciting high (**19n**, *K_i_* = 46.3 nM) or moderate (**19ba**, *K_i_* = 102.0 nM; **19ay**, *K_i_* = 368.1 nM) affinity for this receptor and negligible affinities for the remaining ARs (Table 2). Moreover, three of the pyrimidine-5-carbonitriles prepared exhibited an attractive dual A_2A_AR/A_2B_AR profile (Table 2, compounds **19ab**, **19an**, and **19bf**), which are now being investigated within the context of our anticancer programs.

**Figure 4:**
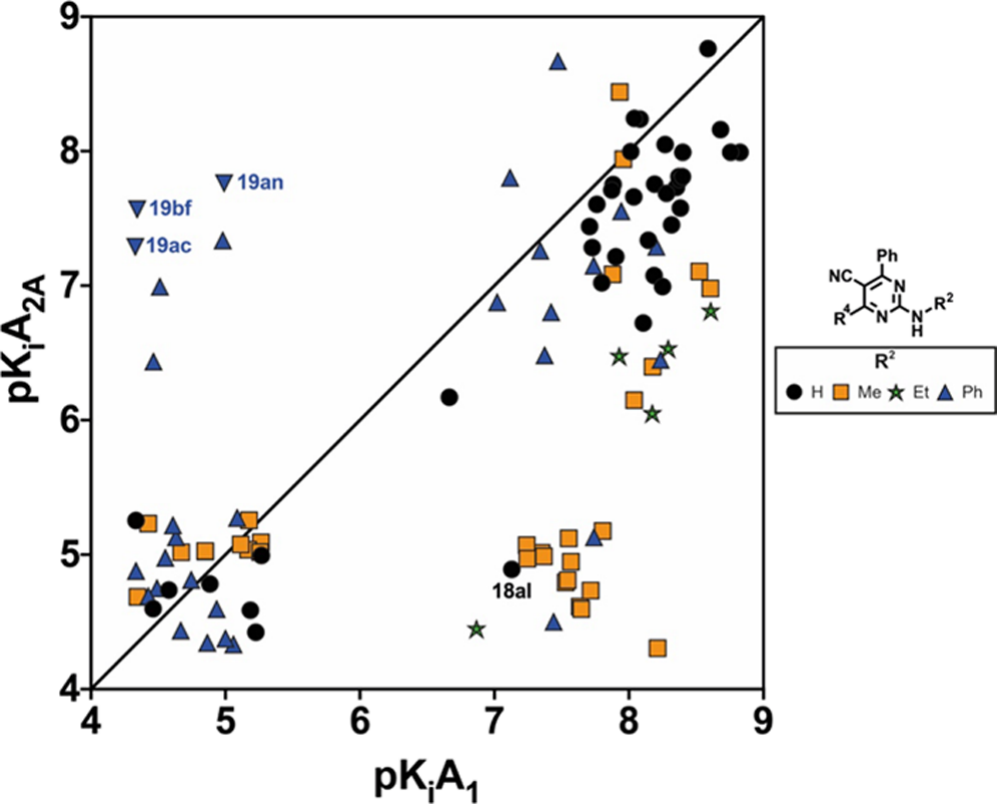
Affinity-selectivity plot for the 2-amino-4,6-diaryl-5-carbonitriles of series **I** (**18a**–**18 am**) and series **II** (**19a**–**19bn**). Inverted triangles show dual A_2A_/A_2B_ compounds.

**Figure 5:**
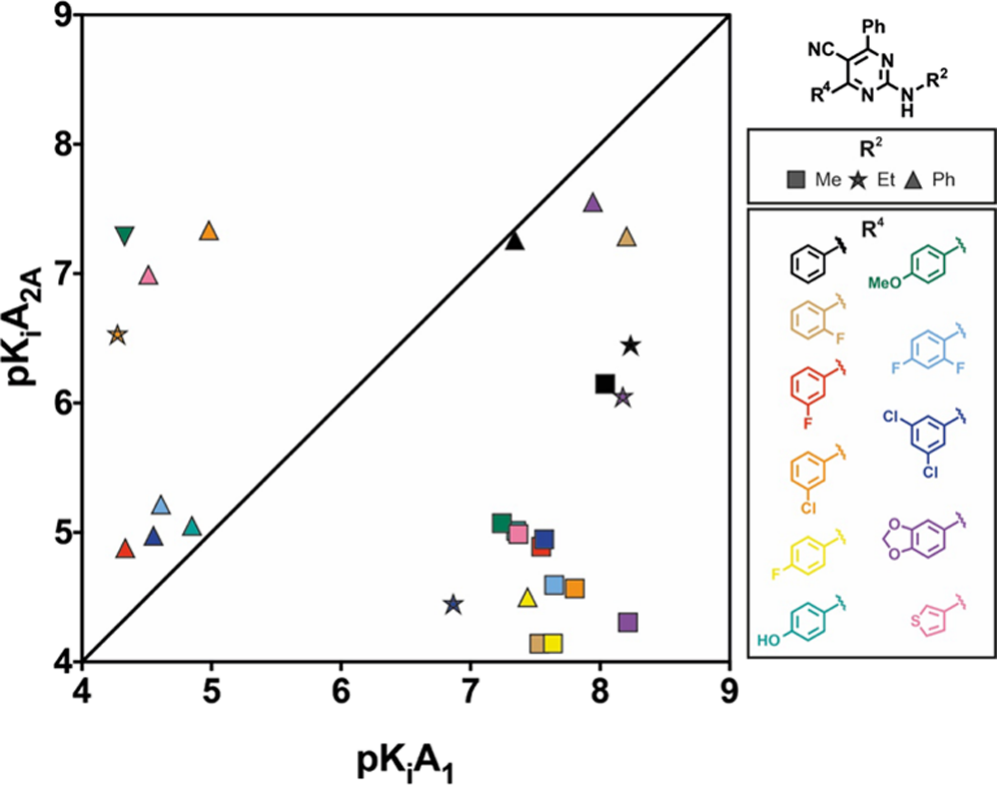
Affinity-selectivity plot of a selection of 2-amino-4,6-diaryl-5-carbonitriles from series **II** (the shape indicates the substituent on R^2^, and the color indicates the substituent on R^4^).

For a more immediate and efficient analysis of the variation of both affinity and selectivity, the binding data of the main series **18** and **19** (Tables 1 and 2) is presented as a function of the p*K*i A_2A_AR (*Y* axis) vs p*K*i A_1_AR (*X* axis) (Figures 4 and 5). Compounds lining around the diagonal of this square plot will bear equal affinities at both receptors, whereas A_1_AR or A_2A_AR selective compounds will cluster on regions below or above the diagonal, respectively, with the distance from the diagonal being directly correlated with their degree of selectivity. In this work, the emphasis is put on compounds with high A_1_AR affinity and high A_1_AR/A_2A_AR selectivity, so we will thus focus on the lower right-hand side corner of the plots.

Series **I** (compounds **18**) always maintained a non-substituted exocyclic amino group and was designed in two subsets: in the first one (Table 1, compounds **18a**–**18ag**), a phenyl ring was maintained invariable at position 6 to explore the effect of diverse substituents at position 4. In the second subset, the chemical groups at positions 4 and 6 were simultaneously modified (Table 1, compounds **18ah**–**18am**). Inspection of the pharmacological data obtained for the whole series of 2-aminopyrimidine-5-carbonitriles (**18**; Figure 4, black circles, and Table 1) reveals that, regardless of the aryl or heteroaryl group present at R^4^ and R^6^, most compounds exhibit a rather dual A_1_AR/A_2A_AR affinity profile. Collectively, these ligands elicit superior (low nanomolar) affinity at A_1_AR but generally exhibiting low (3- to 10-fold) selectivity toward A_2A_AR. This trend can be visualized in Figure 4 (black circles), with most derivatives appearing only slightly under the diagonal in the right part of the plot. The only exception to this trend is observed for **18al**, which elicits modest A_1_AR affinity (*K_i_* = 74.3 nM) and a noticeable selective profile (Table 3).

Interestingly, accompanying their A_1_AR/A_2A_AR profile, a relevant A_2B_AR binding affinity is observed for compounds bearing **3**-substituted phenyl groups or heterocyclic moieties at R^4^ (e.g., **18e**, **18f**, **18g**, **18h**, **18t**, **18u**, **18x**, and **18y**) and R^6^ (**18ah** and **18am**). The absence of any AR affinity in ligands bearing cyclopentyl or cyclohexyl groups at R^4^ (Table 1, compounds **18ad** and **18ae**) confirms the importance of the (hetero)aromatic substituents at these positions. Moreover, the data presented in Table 1 is coherent with the SAR trends observed for a structurally related series (Figure 2),^42−44^ thus indicating that the aromatic moieties at 4 and 6 are critical contributors for recognition and binding at both A_1_AR and A_2A_AR.

Inspection of the data presented in Table 2 (series **II**) reveals the significant effect of the substituent on the amino group (R^2^) in the adenosinergic profile of these series. While these derivatives retain the excellent A_1_AR affinity observed in series **I** discussed above, alkylation at the amino group substantially affects the observed selectivity profile (Table 2 and Figure 5). Thus, pyrimidine-5-carbonitriles bearing a methylamino group at position 2 generally combine high A_1_AR affinity and outstanding selectivity toward A_2A_AR (Table 2, compounds **19j**, **19l**, **19v**, **19z**, **19af**, **19aj**, **19ao**, **19az**, and **19bd**), which appear consequently clustered in the low-right corner of the corresponding selectivity plot (Figure 4, orange squares, and Figure 5). In a clear contrast, the introduction of a phenyl group on the exocyclic amino (R^2^ = Ph) generally afforded either inactive, promiscuous, or, in few cases, A_2A_AR selective derivatives (**19ay** and **19ba**) with moderate affinity. These compounds cluster in the upper-left side of the graph in Figure 4 (blue triangles), together with compounds **19ac**, **19an**, and **19bf** which, as mentioned before, exhibit an attractive dual A_2A_AR/A_2B_AR profile (inverted triangles in Figure 4). In particular, ligand **19bf** has similar affinity at A_2A_AR (*K_i_* = 27.1 nM) and A_2B_AR (*K_i_* = 29.4 nM), constituting a highly attractive pharmacological tool to explore the effect of simultaneous blockage of A_2A_AR and A_2B_AR in A_2_AR-responsive cancer cell lines.

As part of the SAR study, it was decided to briefly explore the effect of introducing an ethyl group at the exocyclic amine (R^2^ = Et; Table 2). These derivatives elicit excellent to satisfactory A_1_AR affinity (*K_i_* = 2.46–135.9 nM), though the selectivity profile toward A_2A_AR is rather moderate (30- to 65-fold, green stars in Figure 4), thus suggesting a very specific effect of the methyl group in the exocyclic amine.

With 66 pyrimidine-5-carbonitrile derivatives, series **II** constitutes the most interesting subset for further exploration. Ligands illustrative of the observed SAR trends were selected for graphical representation (Figure 5) as a function of the p*K*i A_2A_AR (*Y* axis) vs p*K*i A_1_AR (*X* axis). The observation in series **I** where the introduction of two phenyl-substituted residues at R^4^ and R^6^ of the pyrimidine core (Table 1, compounds **18ah**–**18al**) does not improve the affinity/selectivity profile aimed us to (mostly) maintain invariable a phenyl group at R^6^ in series **II** and instead focus on an exhaustive exploration of the substitution patterns at R^4^ with phenyl or heteroaryl groups. For comparative reasons, some derivatives bearing phenyl-substituted residues at positions 4 and 6 were synthesized and tested (Table 2, compounds **19bi**–**19bn**) and are represented in Figure 5. In a clear contrast with their analogues in series **I**, which exhibited a non-selective profile, all derivatives of series **II** bearing two (identical or different) phenyl substituted residues at R^4^ or R^6^ proved to be inactive, irrespectively of the group contained in the exocyclic amino group.

The data on Table 2 and Figure 5 evidence that the substituent at the phenyl group has a clear impact on both affinity and selectivity. Thus, **2**-substituted derivatives (**19d**–**19i**) were either non-selective (2-F) or inactive (2-Cl and 2-OMe), while pyrimidine-5-carbonitriles bearing a **3**-substituted phenyl group at R^4^ (**19j**–**19u**) generally reproduced the non-selective profile observed in series **I**. Within the 3-phenyl substituted derivatives (Figure 5), a 3-chlorophenyl residue led to attractive ligands (**19l** and **19n**). Interestingly, while the 2-methylamino derivative **19l** is a potent (*K_i_* = 15.7 nM) and selective A_1_AR antagonist, its 2-phenylamino analogue (**19n**) exhibits moderate and selective A_2A_AR affinity (*K_i_* = 46.3 nM). The introduction of substituents at position 4 of the R^4^ phenyl group afforded several ligands with excellent (**19v**) to moderate (**19w**, **19z**, **19ab**, **19ad**, and **19ae**) A_1_AR affinity and selectivity (Figure 5). However, as observed early in this series, only ligands bearing a methylamino group at position 2 of the heterocyclic core combined the desired affinity and selectivity profile (e.g., **19v**, **19z**, and **19ab**).

Thirteen derivatives were selected to explore the effect of different disubstituted patterns on the phenyl group at R^4^ (Table 2, compounds **19af**–**19aq**). The SAR trends discussed above for monosubstituted phenyl groups were generally reproduced within this subset (Figure 5), with three pyrimidine-5-carbonitriles (e.g., **19af**, **19aj**, and **19ao**) eliciting excellent A_1_AR affinity and selectivity (Figure 5, blue and purple squares). Among these ligands, **19ao** (Figure 5, purple square) stands out as the most attractive A_1_AR antagonist identified during this study, combining high potency (*K_i_* = 6.11 nM) with excellent selectivity toward the rest of the ARs (Table 2). It should be noticed that, in addition to its 2-methylamino group at 2, **19ao** contains a piperonyl group at R^4^, a relatively frequent motif within A_1_AR antagonists.^18−21^ Further introduction of pentagonal or hexagonal heterocyclic moieties at R^4^ enabled the identification of three potent and selective A_1_AR ligands (**19az**, **19bb**, and **19bd**) that combine 3-thienyl, 4-pyridyl, or 3-pyridyl groups at R^4^ with an exocyclic methylamino group in R^2^ (*K_i_* = 42.8, 56.9, and 19.3 nM, respectively). Interestingly, pyrimidine derivatives bearing 3-thienyl or 3-furyl substituents at R^4^ and an *N*-phenylamino at R^2^ exhibit moderate affinity (*K_i_* = 368 and 102 nM, respectively) and complete selectivity toward the A_2A_AR. This data is coherent with the potent and selective A_2A_AR profile observed for **19n** (Table 2 and Figure 5), which contains a 3-chlorophenyl group at R^4^. Finally, the introduction of a cyclohexyl group at R^4^ or of two substituted phenyl rings at both R^4^ and R^6^ (Table 1, compounds **19bg**–**19bn**) afforded inactive compounds, irrespectively of the substitution pattern at any of the three points of diversity explored (i.e., R^2^, R^4^, and R^6^). Similarly, pyrimidine-5-carbonitriles bearing an *N*,*N*-dimethylamino group at position 2 showed to be inactive (Table 3).

### Molecular Modeling

Taking advantage of the AR experimental crystal structures, we carried on a structure-based analysis of the binding mode of these compound series, addressed to further interpret the SAR observations discussed above. The study consisted of a first phase, where all compounds with measured A_1_AR affinity were docked on both this receptor and the A_2A_AR, leading to two alternative binding models. Each of these binding mode proposals was the bases of extensive free energy perturbation (FEP) simulations on the A_1_AR, which univocally selected one binding mode and allowed a quantitative interpretation of the observed SAR, setting the grounds to further structure-based design optimizations.

**Figure 6:**
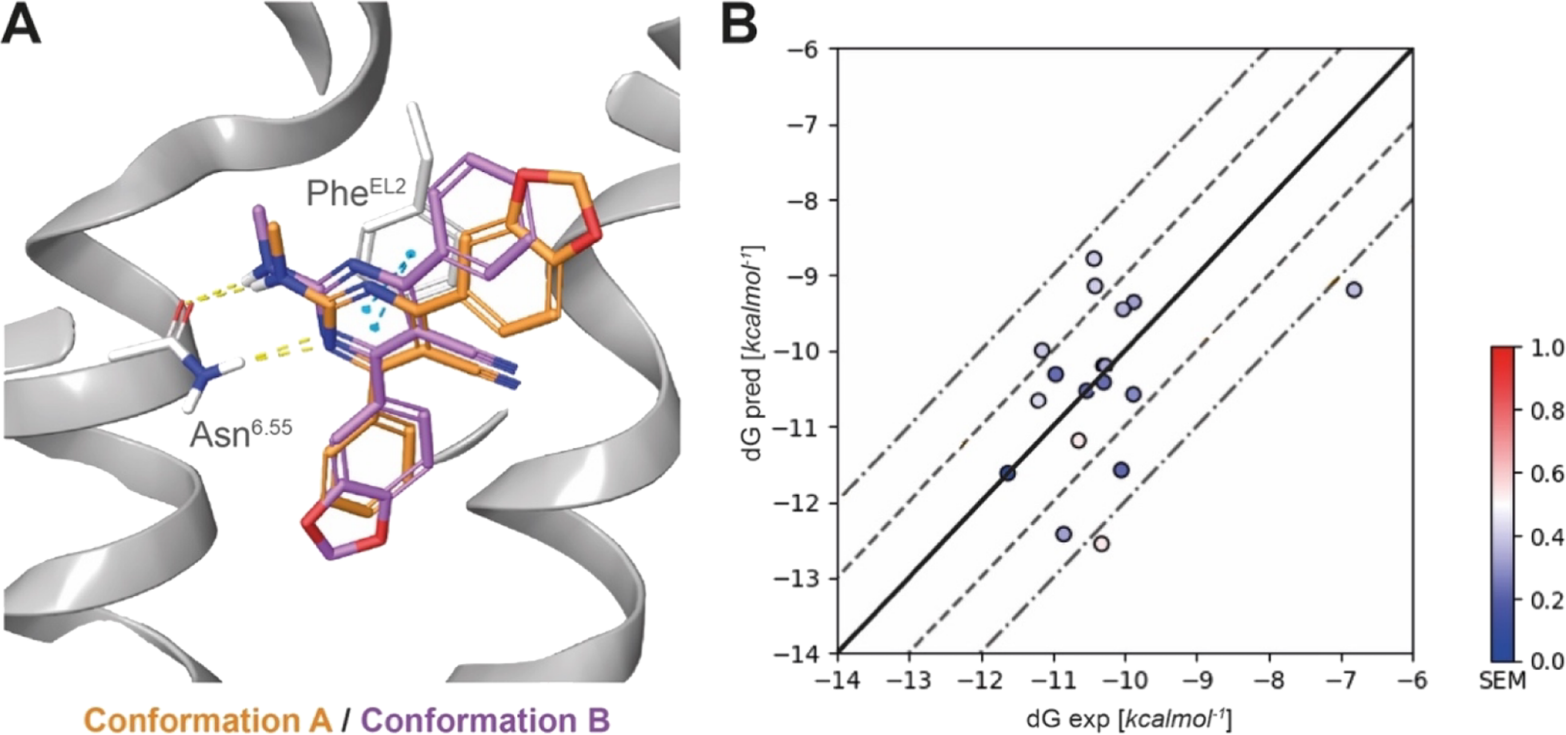
(A) Two binding modes considered for this series (conformation A, orange; conformation B, magenta) illustrated on compound **19ao** on the A_1_AR (PDB: 5N2S). (B) Scatter plot of the predicted (vertical axis) vs experimental (horizontal axis) binding free energies for the A_1_AR, as determined by FEP calculations using conformation A. The dots are colored according to the SEM of the associated FEP simulations after cycle closure correction (see Experimental Section).

The two alternative binding modes arose as a consequence of the asymmetric substitution pattern of these compounds. In both orientations, the central heterocycle and exocyclic amino group maintain the two key hydrogen bonds with the side chain of Asn^6.55^, totally conserved within the ARs (Figure 6A). The two binding modes essentially differ on the orientation of the bulkiest substituent (at R^4^ or R^6^), which is either located at the extracellular loop region (Figure 6A, conformation A, orange) or within the deep TM cavity of the receptor (Figure 6B, conformation B, magenta). To identify the most probable binding mode, each binding mode was the starting point of a series of FEP calculations performed on a selection of compounds from series **II**. The criteria of selection were to cover a wide span of experimental affinities on the A_1_AR and sufficient structural diversity while retaining the most interesting scaffolds from the medicinal chemistry perspective, resulting in an initial subset of 21 A_1_AR antagonists where R^2^ = Me. From these, we further retained those compounds where a change in R^2^ would lead to a substantial change in their experimental selectivity profile, leading to a final selection of 18 compounds (**19a**, **19d**, **19j**, **19l**, **19s**, **19v**, **19z**, **19ab**, **19ad**, **19af**, **19aj**, **19am**, **19ao**, **19at**, **19ax**, **19az**, **19bb**, and **19bd**). The dataset was studied on each binding pose through 28 FEP pair comparisons, see Figure S1. Each FEP cycle was performed with the QligFEP protocol,^57^ leading to estimated relative affinities between each compound pair (ΔΔ*G*_bind_). The absolute binding affinity (Δ*G*_bind_, kcal/mol) was then calculated with a cycle closure correction approach following the idea presented by Wang et al.^58^ (see Experimental Section). The results clearly favor conformation A (Figure 6B, MUE = 0.87 ± 0.17 kcal/mol, RMSE = 1.13 ± 0.18 kcal/mol, and SEM = 0.34 ± 0.03 kcal/mol) as it shows better predictivity and convergence than the alternative conformation B (MUE = 1.68 ± 0.39, RMSE = 2.26 ± 0.59, and SEM 1.12 ± 0.1, Figure S2). Consequently, conformation A was retained for further analysis.

The structural binding model “conformation A” shows a pattern of ligand–receptor interactions that is compatible with the binding mode of monocyclic compounds predicted in the A_3_ and A_2B_ ARs as a part of our ligand design programs on these receptors.^47,59,60^ A qualitative structural analysis of all docked compounds with measured affinity, on both A_1_AR and A_2A_AR, allowed us to rationalize the experimentally observed differences in selectivity. The series of free-amine compounds (series **I**, A_1_/A_2A_ non-selective profile) invariably shows a conserved interaction pattern with both A_1_AR and A_2A_AR, consisting of the double-hydrogen bond with Asn^6.55^ (Asn254/253 in A_1_/A_2A_ AR, respectively) and a π-stacking between the aromatic core and the conserved phenylalanine in EL2 (Phe171/168^EL2^).

**Figure 7:**
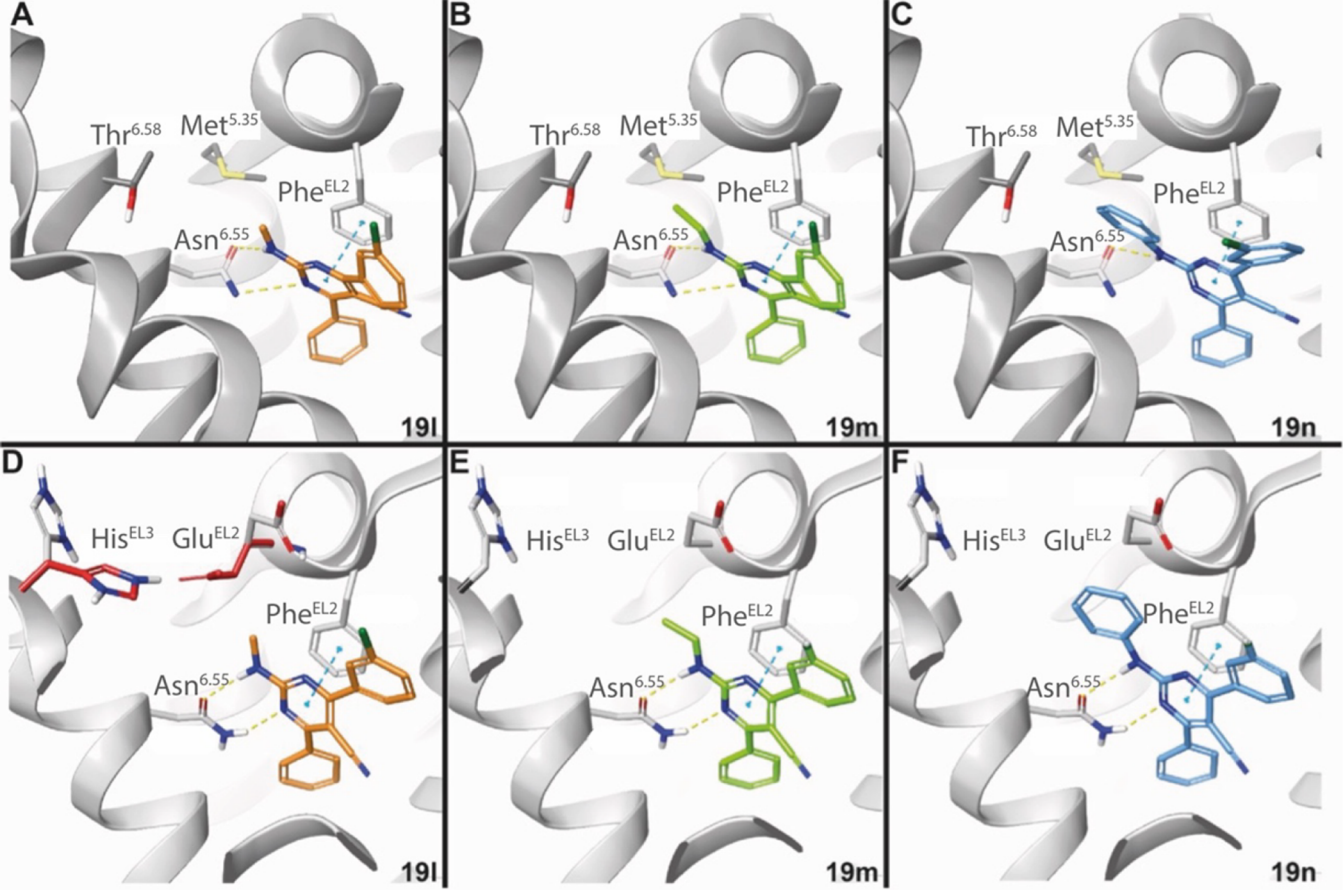
Binding mode to the A_1_AR (A–C, PDB: 5N2S) and the A_2A_AR (D, PDB: 4EIY with closed conformation, red; D–F, PDB: 3UZC, open conformation, gray) of N-substituted compounds: **19l**, R^2^ = Me, orange (A, D); **19m**, R^2^ = Et, green (B, E); **19n**, R^2^ = Ph, blue (C, F).

The substituents at R^2^ explored within series **II**, however, show different behaviors at A_1_AR and A_2A_AR (Figure 7). Generally speaking, N-alkylation (series **II**) causes a decrease in affinity compared with the free-amine compounds (series **I**) in both the AR subtypes. It is indeed the relative loss in affinity for one or another receptor that drives that gain in A_1_AR selectivity, with *N*-methyl and *N*-ethyl derivatives showing a much smaller loss of affinity in A_1_AR than A_2A_AR, a pattern that is somehow inverted in *N*-phenyl substituted compounds. The binding model resulting from our computational study (Figure 7) offers a structural interpretation of these tendencies. *N*-Methyl (Figure 7A) and *N*-ethyl (Figure 7B) compounds can maintain the dual H-bond in the A_1_AR with Asn254^6.55^ observed on the free-amine compounds in series **I**, though showing some difficulty in accommodating the new substituents within the pocket defined by Thr259^6.58^ and Met177^5.35^. A bulky phenyl group at R^2^, however, has a greater impact in obstructing the double-hydrogen bond formation (Figure 7C), explaining a greater loss in affinity than the methyl and ethyl substituted compounds. The difference between the two receptors in responding to these substitutions resides on the two possible conformations of the subpocket accommodating the R^2^ substituent in the A_2A_AR: open (Figure 7D–F, gray) and closed (Figure 7D, red), as defined by the absence or presence, respectively, of the salt bridge between His264^EL3^ and Glu169^EL2^ connecting EL3 and EL2. *N*-Methyl and *N*-ethyl bearing compounds can be hardly accommodated in the closed conformation of the A_2A_AR (Figure 7D), neither they can stabilize the A_2A_AR open conformation (Figure 7E). *N*-Phenyl compounds, on the other side, clearly stabilize this A_2A_AR open conformation by hydrophobic interactions (Figure 7F), providing a rationale for their increased selectivity for this receptor. A further look into the complex of A_2A_AR with the congeneric series formed by compounds **19l** (R^2^ = Me), **19l** (R^2^ = Et), and **19n** (R^2^ = Ph) illustrates this idea (Figure S3). To further confirm this hypothesis, we conducted unbiased MD simulations of the A_2A_AR in complex with the methyl (**19l**) and phenyl (**19n**) derivatives and monitored the distance between His264^EL3^ and Glu169^EL2^. The results (Figure S4) show how the latter is incompatible with a closed conformation without really stabilizing the open alternative, while the *N*-methyl derivative promotes a closing of the loops.

## Conclusions

In summary, we have disclosed a large collection of 2-amino-4,6-disubstituted-pyrimidine derivatives as potent, structurally simple, and highly selective A_1_AR ligands. The pharmacological characterization of the most attractive A_1_AR ligands identified during this study confirmed its antagonistic behavior (through cAMP assays). Further studies to complete the bioavailability profile and in vivo BBB permeation of lead compounds are currently in progress. The reliable and efficient three-component reaction facilitated the rapid assembly of a large library, thus enabling to comprehensively examinate the most prominent features of the SAR and SSR in this series. This building-block scheme is an asset in our lab to further grow the chemical library, guided by the rationale derived from this work. The SSR studies highlighted the influence of the aromatic residues at R^4^ and R^6^ of the pyrimidine core to the selectivity profile but most importantly the prominent role exerted by the methylation of the 2-amino group as the main contributor to the unprecedented A_1_AR selectivity profile observed in these series. The SAR trends herein disclosed were complemented and interpreted with a comprehensive computational modeling analysis based on rigorous FEP simulations, starting from the receptor-driven docking model that initially guided the design of these series. Particularly revealing was the orientation of the new asymmetrically substituted scaffold, for which the binding mode on the A_1_AR was herein supported by first-principle binding affinity calculations, which can be therefore used in the next stage of ligand optimization.

## Experimental Section

### Chemistry

Unless otherwise indicated, all starting materials, reagents, and solvents were purchased and used without further purification. After extraction from aqueous phases, the organic solvents were dried over anhydrous sodium sulfate. The reactions were monitored by thin-layer chromatography (TLC) on 2.5 mm Merck silica gel GF 254 strips, and the purified compounds each showed a single spot; unless stated otherwise, UV light and/or iodine vapor were used to detect compounds. The Biginelli reactions were performed in coated Kimble vials on a PLS (6 × 4) organic synthesizer with orbital stirring. The purity and identity of all tested compounds were established by a combination of high-performance liquid chromatography (HPLC), elemental analysis, mass spectrometry, and NMR spectroscopy as described below. Purification of isolated products was carried out by column chromatography (Kieselgel 0.040–0.063 mm, E. Merck) or medium pressure liquid chromatography (MPLC) on a CombiFlash Companion (Teledyne ISCO) with RediSep pre-packed normal-phase silica gel (35–60 μm) columns followed by recrystallization. Melting points were determined on a Gallenkamp melting point apparatus and were uncorrected. The NMR spectra were recorded on Bruker AM300 and XM500 spectrometers. Chemical shifts were given as δ values against tetramethylsilane as the internal standard, and *J* values were given in Hz. Mass spectra were obtained on a Varian MAT-711 instrument. High-resolution mass spectra were obtained on an Autospec Micromass spectrometer. Analytical HPLC was performed on an Agilent 1100 system using an Agilent Zorbax SB-Phenyl, 2.1 mm × 150 mm, 5 μm column with gradient elution using the mobile phases (A) H_2_O containing 0.1% CF_3_COOH and (B) MeCN and a flow rate of 1 mL/min. All reported compounds are >95% pure by HPLC analysis. HPLC traces obtained for representative lead compounds herein identified are provided in the Supporting Information. The structural and spectroscopic data obtained for all compounds described are provided in the Supporting Information.

### General Procedure for the Three-Component Synthesis of 2-Amino-4,6-diarylpyrimidin-5-carbonitriles (**18**–**20**)

A mixture of α-cyanoketone **21a**–**j** (1 mmol), aldehyde **22a**–**j** (1 mmol), the guanidine salt **23a**–**d** (1.2 mmol), and Na_2_CO_3_ (3 mmol) in 3 mL of THF in coated Kimble vials was stirred with orbital stirring at 80 °C for 12 h. After completion of the reaction (controlled by TLC), the solvent was evaporated to dryness and the resulting residue was resuspended in water and extracted with ethyl acetate. The organic phase was dried with Na_2_SO_4_ and evaporated to dryness, when the oily residue was resuspended with methanol the product generally precipitates, was filtered, and purified by recrystallization or column chromatography (silica gel) generally using hexane/AcOEt mixtures as the eluent.

### Pharmacological Characterization

Radioligand binding competition assays were performed *in vitro* using human ARs expressed in transfected HeLa [*h*A_2A_AR (9 pmol/mg protein), *h*A_3_AR (3 pmol/mg protein)], HEK-293 [*h*A_2B_AR (1.5 pmol/mg protein)], and CHO [*h*A_1_AR (1.5 pmol/mg protein)] cells as described previously.^46−48,59^ A brief description is given below. A_1_AR competition binding experiments were carried out in membranes from CHO-A_1_ cells labeled with 1 nM [^3^H]DPCPX (*K*_D_ = 0.7 nM). Non-specific binding was determined in the presence of 10 μM R-PIA. The reaction mixture was incubated at 25 °C for 60 min. A_2A_AR competition binding experiments were carried out in membranes from HeLa-A_2A_ cells labeled with 3 nM [^3^H]ZM241385 (*K*_D_ = 2 nM). Non-specific binding was determined in the presence of 50 μM NECA. The reaction mixture was incubated at 25 °C for 30 min. A_2B_AR competition binding experiments were carried out in membranes from HEK-293-A_2B_ cells (Euroscreen, Gosselies, Belgium) labeled with 25 nM [^3^H]DPCPX (*K*_D_ = 21 nM). Non-specific binding was determined in the presence of 400 μM NECA. The reaction mixture was incubated at 25 °C for 30 min. A_3_AR competition binding experiments were carried out in membranes from HeLa-A_3_ cells labeled with 10 nM [^3^H]NECA (*K*_D_ = 8.7 nM). Non-specific binding was determined in the presence of 100 μM R-PIA. The reaction mixture was incubated at 25 °C for 180 min. After the incubation time, membranes were washed and filtered and radioactivity was detected in a Microbeta Trilux reader (PerkinElmer).

### Solubility Determinations

The stock solutions (10^–2^ M) of the selected ligands were diluted to decreased molarity, from 300 to 0.1 μM, in a 384-well transparent plate (Greiner 781801) with 1% DMSO:99% PBS buffer. These were incubated at 37 °C and read after 2 h in a NEPHELOstar Plus (BMG LABTECH). The results were adjusted to a segmented regression to obtain the maximum concentration in which compounds are soluble.

### Human Microsomal Stability

The human microsomes employed were purchased from Tebu-Xenotech. The compound was incubated with microsomes at 37 °C in a 50 mM phosphate buffer (pH = 7.4) containing 30 mM MgCl_2_, 10 mM NADP, 100 mM glucose-6-phosphate, and 40 U/mL glucose-6-phosphate dehydrogenase. Samples (75 μL) were taken from each well at 0, 10, 20, 40, and 60 min and transferred to a plate containing 75 μL of acetonitrile (4 °C), and 30 μL of 0.5% formic acid in water was added for improving the chromatographic conditions. The plate was centrifuged (4000*g*, 60 min), and supernatants were taken and analyzed in a UPLC-MS/MS (Xevo-TQD, Waters) by employing a BEH C18 column and an isocratic gradient of 0.1% formic acid in water:0.1% formic acid acetonitrile (60:40). The metabolic stability of the compounds was calculated from the logarithm of the remaining compounds at each of the time points studied.

### Functional Experiments

cAMP assays were performed at human A_1_ARs using a cAMP enzyme immunoassay kit (Amersham Biosciences). CHO cells were seeded (10,000 cells per well) in 96-well culture plates and incubated at 37 °C in an atmosphere with 5% CO_2_ in Nutrient Mixture F-12 Ham (Ham’s F-12) containing 10% fetal bovine serum dialyzed (FBS), penicillin/streptomycin (1%), amphotericin B (2.5 μg/mL), and Geneticin (400 μg/mL). Cells were washed 2× with 200 μL of the assay medium (Ham’s F-12 and 25 mM HEPES pH = 7.4) and pre-incubated with the assay medium containing 20 μM rolipram and test compounds at 37 °C for 15 min. Stimulation was carried out by the addition of 0.1 μM NECA incubated for 10 min and 3 μM forskolin incubated for 5 min at 37 °C (total incubation time, 30 min). Reaction was stopped with lysis buffer supplied in the kit, and the enzyme immunoassay was carried out for detection of intracellular cAMP at 450 nm in an Ultra Evolution detector (Tecan). For data analysis, IC_50_ values were obtained by fitting the data with non-linear regression using Prism 5.0 software (GraphPad, San Diego, CA). For those compounds that showed either little affinity or poor solubility, a percentage inhibition of specific binding was reported. Results are the mean of three experiments (*n* = 3) each performed in duplicate.

### Calcein-AM Experiments

Calcein cell accumulation was evaluated by following a previously described method.^53−55^ The MDCK-MDR1 cell line (30,000 cells per well) was seeded into a 96-well black culture plate with 100 μL of the medium and allowed to become confluent overnight. Test compounds (100 μL) were solubilized in the culture medium and added to monolayers, with final concentrations ranging from 0.1 to 100 μM. The 96-well plate was incubated at 37 °C for 30 min. Calcein-AM was added in 100 μL of phosphate buffered saline (PBS) to yield a final concentration of 2.5 μM, and the plate was incubated for 30 min. Each well was washed three times with ice-cold PBS. Saline buffer was added to each well, and the plate was read with Victor^3^ (PerkinElmer) at excitation and emission wavelengths of 485 and 535 nm, respectively. In these experimental conditions, Calcein cell accumulation in the absence and in the presence of tested compounds was evaluated and the fluorescence basal level was estimated with untreated cells. In treated wells, the increase in fluorescence with respect to the basal level was measured. EC_50_ values were determined by fitting the fluorescence increase percentage versus log[dose].

### Protein Preparation and Ligand Docking

Receptor structures were retrieved from the PDB with codes 5N2S (*h*A_1_AR), 4EIY (*h*A_2A_AR-closed), and 3UZC (*h*A_2A_AR-open) and prepared for ligand docking and MD simulations. The initial preparation steps were performed with the Schrödinger suite (protein preparation wizard) and included modeling of the missing loop segments, reverting the protein construct to the wt sequence, addition of protons, and assessment of Asn/Gln/His rotamers and protonation states (in all cases, Asp, Glu, Lys, and Arg residues were assigned in their default charged state). All His residues in both receptors were modeled as neutral with the proton on Nδ except for His^6.52^, protonated on Nε, and His264 in A_2A_AR that is positively charged. Each receptor structure was then inserted in the membrane and equilibrated under periodic boundary conditions (PBC) using the PyMemDyn protocol described elsewhere.^61^ Shortly, the receptor was embedded in a pre-equilibrated membrane consisting of POPC (1-palmitoyl-2-oleoyl phosphatidylcholine) lipids, with the TM bundle aligned to its vertical axis. An hexagonal prism-shaped box was then built and soaked with bulk water; thereafter, the system was energy-minimized with GROMACS 4.6.^62^ using the OPLS-AA force field^63^ for combination with the Berger parameters for lipids.^64^ An energy minimization of the system (50,000 conjugate gradient steps, convergence criteria of 1000 kJ/mol) precedes a short (2.5 ns length) MD equilibration, where initial restraints imposed on protein heavy atoms are gradually released as described in detail in our original protocol.^61^ The final receptor structure was energy-minimized with similar settings as above.

An automated docking exploration was performed with GlideXP,^65^ applying default parameters, for ligands **18a**, **19a**, **19b**, and **19c** as model compounds of free-amine, methylamine, ethylamine, and phenylamine derivatives, respectively. These ligands were initially built in their 2D structures, and the SD file generated was the input for the ligand preparation wizard in Schrödinger, which generated the most probable protonation state and an energy-minimized 3D conformer with the OPLS3 force field. The search box was defined by the co-crystallized ligand in each case, resulting in very similar boxes since all ligands occupy the same orthosteric site. We used the results of this automated docking exploration to build the corresponding complexes with an expanded dataset of 60 ligands, consisting of the compounds from series **II** that have measurable *K_i_* affinity values for either A_1_AR or A_2A_AR, plus the analogous of these on series **I** (Tables 1 and 2). Each of these compounds was directly built from the structurally closest ligand–receptor complex, from those generated by automated docking (i.e., **18a**, **19a**, **19b**, and **19c**), and energy minimization of the resulting complex followed (default parameters within the Schrödinger suite).

### MD and FEP Calculations

Selected receptor–ligand complexes were grouped in a set of pair comparisons for free energy perturbation (FEP) calculations using the QligFEP protocol^57^ and the MD software Q.^66,67^ The so-called FEP pathway (see Figure S1) was designed based on maximal compound similarity, computed upon Morgan Fingerprint descriptors, with a series of corrections to ensure a cycle closure correction. This approach allows the estimation of absolute binding free energies (Δ*G*_bind_) using the experimental value of one compound in the series as a reference, together with the associated statistical figures of merit: the mean unassigned error (MUE) and root mean squared error (RMSE), between calculated and experimental binding affinities, together with the convergence obtained along the calculations (expressed as standard error of the mean, SEM), in all cases in kcal/mol. Confidence intervals for the regression metrics were estimated using bootstrap sampling.

A 25 Å sphere centered on the center of geometry of the ligand is considered for MD simulations under spherical boundary conditions. Protein atoms in the boundary of the sphere (22–25 Å outer shell) had a positional restraint of 20 kcal/mol/Å^2^, while solvent atoms were subject to polarization and radial restrains using the surface constrained all-atom solvent (SCAAS)^66,68^ model to mimic the properties of bulk water at the sphere surface. Atoms lying outside the simulation sphere were tightly constrained (200 kcal/mol/Å^2^ force constant) and excluded from the calculation of non-bonded interactions. Long-range electrostatic interactions beyond a 10 Å cutoff were treated with the local reaction field method,^69^ except for the atoms undergoing the FEP transformation where no cutoff was applied. Solvent bonds and angles were constrained using the SHAKE algorithm.^70^ All titratable residues outside the sphere were neutralized, and histidine residues were assigned a hydrogen atom on the δ nitrogen. Residue parameters were translated from the OPLS-AA/M force field,^71^ and the ligand parameters were generated using the ffld server as implemented in the Schrödinger suite and the lipid parameters as described above. The simulation sphere was warmed up from 0.1 to 298 K, during a first equilibration period of 0.61 ns, where an initial restraint of 25 kcal/mol/Å^2^ imposed on all heavy atoms was slowly released for all complexes. Thereafter, the system was subject to 10 parallel replicates of unrestrained MD, where the following FEP protocol was applied for each ligand transformation: an initial 0.25 ns unbiased equilibration period, with different initial velocities for each replica, was followed by 101 FEP λ-windows, consisting of 10 ps each, distributed using a sigmoidal sampling schedule. During the FEP transformation, the potentials of the two ligands involved were combined using a double topology scheme.^57^ To fulfill a thermodynamic cycle and calculate relative binding free energies, analogous FEP transformations were run for the same ligand pair in a sphere of water, maintaining the same MD parameters (i.e., sphere size, simulation time, etc.). The relative binding free energy difference was then estimated by solving the thermodynamic cycle using the Bennett acceptance ratio (BAR).^72^

## Supporting Information Available

Molecular formula strings (CSV)
Homology models and binding mode of compounds **18**–**20** as well as PDB codes of the structures used for models 5N2S (*h*A_1_AR), 4EIY (*h*A_2A_AR-closed), and 3UZC (*h*A_2A_AR-open) with the authors releasing the atomic coordinates upon article publication (ZIP)
^1^H NMR, ^13^C NMR, and mass spectra data of all compounds described as well as HPLC traces of lead compounds (PDF)

## Abbreviations Used

ARs: adenosine receptors
BBB: blood brain barrier
cAMP: cyclic adenosine monophosphate
CNS: central nervous system
COPD: chronic obstructive pulmonal disease
Cpd: compound
EM: electronic microscopy
FEP: free energy perturbation
P-gp: P-glucoprotein
SAR: structure–activity relationship
SSR: structure–selectivity relationship
