# Supplementary material for: Optimization of 2-Amino-4,6-diarylpyrimidine-5-carbonitriles as Potent and Selective A1 Antagonists
Source: J Med Chem. 2022 Jan 22;65(3):2091–106. doi: 10.1021/acs.jmedchem.1c01636 (PMC8842224; doi:10.1021/acs.jmedchem.1c01636)
Supplement: Supplementary file 3 — jm1c01636_si_003.pdf [file jm1c01636_si_003.pdf]

SUPPORTING INFORMATION

Optimization of 2-Amino-4,6-diarylpyrimidine-5-carbonitriles as Potent and Selective A_1 Antagonists

Cristina Val,[1,2§] Carlos Rodríguez-García,[1,2§] Rubén Prieto-Díaz,[1,2,5] Abel Crespo,[1,2] Jhonny Azuaje,[1,2] Carlos Carbajales,[1,2] Maria Majellaro,[1,2] Alejandro Díaz-Holguín,[5] José M. Brea,[3*] Maria Isabel Loza,[3] Claudia Gioé-Gallo,[1,2] Marialessandra Contino,[4] Angela Stefanachi,[4] Xerardo García-Mera,[2] Juan C. Estévez,[1] Hugo Gutiérrez-de-Terán,[5*] and Eddy Sotelo[1,2*]

[1]Centro Singular de Investigación en Química Biolóxica e Materiais Moleculares (CiQUS) and [2]Departamento de Química Orgánica, Universidade de Santiago de Compostela, Santiago de Compostela, 15782, Spain. [3]Centro Singular de Investigación en Medicina Molecular y Enfermedades Crónicas (CiMUS). Universidade de Santiago de Compostela, Santiago de Compostela, 15782, Spain. [4]Dipartimento di Farmacia-Scienze del Farmaco, Università degli Studi di Bari ALDO MORO, via Orabona 4, Bari, 70125, Italy. [5]Department of Cell and Molecular Biology, Uppsala University, Uppsala 75124, Sweden.

[§]These authors contributed equally to this work

*To whom correspondence should be addressed:
(ES) +34 881815732, Fax.: +34-881815704, e-mail: e.sotelo@usc.es
(HGT) +46(0)184715056, e-mail: hugo.gutierrez@icm.uu.se

(JB): +34 881815459, Fax.: +34-8818115474, e-mail: pepo.brea@usc.es

TABLE OF CONTENTS

Chemistry. General information........S2
Spectroscopic and analytical data for compounds described........S3
Supplementary Figures........S18
Supplementary Tables........S22
HPLC traces for lead compounds........S25
References........S31

Chemistry. General information

Commercially available starting materials, reagents and solvents were purchased and used without further purification. The reactions were monitored by thin-layer chromatography (TLC) with 2.5 mm Merck silica gel GF 254 strips, and the purified compounds each showed a single spot; unless stated otherwise, UV light, *p*-anisaldehyde solution and/or iodine vapor were used for detection of compounds. The Biginelli reactions were performed in coated Kimble vials on a PLS (6X4) Organic Synthesizer with orbital stirring. Purity and identity of all tested compounds were established by a combination of mass spectrometry, HRMS and NMR spectra as described below. Purification of isolated products was carried out by column chromatography (Kieselgel 0.040–0.063 mm, E. Merck) or medium pressure liquid chromatography (MPLC) on a ComBiFlash Companion (Teledyne ISCO) with RediSep pre-packed normal-phase silica gel (35-60 μm) columns. Melting points were determined on a Stuart Scientific melting point apparatus and are uncorrected.

The NMR spectra were recorded on Bruker AM300 and XM500 spectrometers. Chemical shifts are given as δ values against tetramethylsilane as internal standard and J values are given in Hz. Mass spectra were obtained on a Varian MAT-711 instrument. High-resolution mass spectra were obtained on an Autospec Micromass spectrometer. The purity of all tested compounds was determined to be >95%. All reported compounds are >95% pure by HPLC analysis. HPLC traces obtained for representative lead compounds herein identified are provided in the supporting information. The structural and spectroscopic data obtained for all compounds described is provided.

General procedure for the three-component synthesis of 2-Amino-4,6-diarylpyrimidin-5-carbonitriles (18-20). A mixture of α-cyanoketone **21a-j** (1 mmol), aldehyde **22a-j** (1 mmol), the guanidine salt **23a-d** (1.2 mmol) and Na_2CO_3 (3 mmol) in 3 mL of THF in coated Kimble vials was stirred with orbital stirring at 80ºC for 12h. After completion of the reaction (controlled by TLC), the solvent is evaporated to dryness, the resulting residue is resuspended in water and extracted with ethyl acetate. The organic phase is dried with Na_2SO_4 and evaporated to dryness, when the oily residue is resuspended with methanol the product generally precipitates, is filtered and purified by recrystallization or column chromatography (silica gel) generally using Hexane/AcOEt mixtures as eluent.

Spectroscopic and analytical data for compounds described

2-amino-4,6-diphenylpyrimidine-5-carbonitrile (18a).[1] Yield: 58%. Mp 214-216ºC. ^{1}H NMR (300 MHz, $CDCl_3$), δ (ppm): 7.94-7.91 (m, 4H), 7.57-7.49 (m, 6H), 5.93 (brs, 2H). ^{13}C NMR (75 MHz, $CDCl_3$) δ (ppm): 171.0, 162.0, 135.9, 131.1, 128.7, 128.5, 117.8, 71.6. HRMS (ESI) *m/z* calcd for $C_{17}H_{12}N_4$ $[M+H]^+$: 273.3040; found: 273.1140.

2-amino-4-(2-fluorophenyl)-6-phenylpyrimidine-5-carbonitrile (18b). Yield: 47%. Mp 207-208ºC. ^{1}H NMR (300 MHz, $CDCl_3$), δ (ppm): 7.97-7.94 (m, 2H), 7.60-7.52 (m, 5H), 7.33-7.22 (m, 2H), 6.07 (brs, 2H). MS (EI) *m/z*: 290.1 (89, M^+), 289.1 (100), 272.1 (27), 271.1 (52), 247.1 (10), 145.1 (24), 127.1 (12), 77.1 (11).

2-amino-4-(2-chlorophenyl)-6-phenylpyrimidine-5-carbonitrile (18c). Yield: 59%. Mp 217-218ºC. ^{1}H NMR (300 MHz, DMSO-d_6), δ (ppm): 7.96 (s, 2H), 7.93-7.78 (m, 4H), 7.65-7.53 (m, 5H). MS (EI) *m/z*: 308.1 (22) 307.1 (33), 306.1 (M^+,37), 305.1 (100), 304 (36).

2-amino-4-(2-methoxyphenyl)-6-phenylpyrimidine-5-carbonitrile (18d). Yield: 75%. Mp 198-199ºC. ^{1}H NMR (300 MHz, $CDCl_3$), δ (ppm): 8.16 – 8.10 (m, 2H), 7.99 (dd, J = 8.3, 1.3 Hz, 1H), 7.56 – 7.48 (m, 2H), 7.44 – 7.37 (m, 2H), 7.23 (td, J = 7.5, 1.3 Hz, 1H), 6.85 (dd, J = 7.7, 1.3 Hz, 1H), 5.71 (brs, 2H), 3.94 (s, 3H). HRMS (EI) *m/z* calcd for $C_{18}H_{14}N_4O$ $[M^+]$: 302.1168; found: 302.1090.

2-amino-4-(3-fluorophenyl)-6-phenylpyrimidine-5-carbonitrile (18e). Yield: 70%. Mp 245−246°C. ^{1}H NMR (300 MHz, DMSO-d_6), δ (ppm): 7.96 (bs, 2H), 7.89-7.85 (m, 2H), 7.73-7.54 (m, 6H), 7.53-7.40 (m, 1H).^{13}C NMR (75 MHz, DMSO-d_6) δ (ppm): 170.7, 169.4, 163.8, 162.8, 159.9, 138.9, 136.5, 131.0, 128.8, 128.5, 125.1, 118.4, 117.9, 115.9, 91.2. HRMS (ESI) *m/z* calcd for $C_{17}H_{11}FN_4$ $[M+H]^+$: 291.1046; found: 291.1041.

2-amino-4-(3-chlorophenyl)-6-phenylpyrimidine-5-carbonitrile (18f). Yield: 59%. Mp 261-262ºC. ^{1}H NMR (300 MHz, DMSO-d_6), δ (ppm): 8.70-7.69 (m, 6H), 7.71-7.43 (m, 5H). MS (EI) *m/z*: 307.2 (33), 306.2 (M^+, 92), 305.1 (100), 288.1 (42), 271.1 (85), 254.1 (42).

2-amino-4-(3-methoxyphenyl)-6-phenylpyrimidine-5-carbonitrile (18g). Yield: 75%. Mp 196-198ºC. ^{1}H NMR (300 MHz, DMSO-d_6), δ (ppm): 7.97-7.83 (m, 4H), 7.61-7.40 (m, 6H), 7.19-7.12 (m, 1H), 3.83 (s, 3H). HRMS (EI) *m/z*: calcd for $C_{18}H_{14}N_4O$ $[M^+]$: 302.1168, found: 302.1160.

2-amino-4-(3-hydroxyphenyl)-6-phenylpyrimidine-5-carbonitrile (18h). Yield: 46%. Mp 249−251ºC. ^{1}H NMR (300 MHz, DMSO-d_6), δ (ppm): 9.75 (s, 1H, OH), 7.85-7.82 (m, 4H), 7.55-7.52

(m, 3H), 7.36-7.32 (m, 3H), 6.96-6.93 (m, 1H).^{13}C NMR (75 MHz, DMSO-d_6) δ (ppm): 170.8, 170.7, 162.8, 157.3, 137.8, 136.6, 130.9, 129.5, 128.8, 128.4, 119.5, 118.6, 117.8, 115.6, 91.0. HRMS (ESI) *m/z* calcd for $C_{17}H_{12}N_4O$ [M+H]$^+$: 289.1089; found: 289.1084.

2-amino-4-(3-cyanophenyl)-6-phenylpyrimidine-5-carbonitrile (18i). Yield: 39%. Mp 218-219ºC. ^{1}H NMR (300 MHz, DMSO-d_6), *δ* (ppm): 8.37-8.14 (m, 2H), 8.14-7.95 (m, 3H), 7.95-7.72 (m, 3H), 7.67-7.50 (m, 3H).^{13}C NMR (75 MHz, $CDCl_3$) δ (ppm): 170.6, 168.9, 162.8, 137.7, 136.4, 134.3, 133.5, 132.6, 131.1, 129.9, 128.8, 128.5, 118.3, 111.6, 95.1, 91.4. HRMS (ESI) *m/z* calcd for $C_{18}H_{11}N_5$ [M+H]$^+$: 298.1092, found: 298.1087.

2-amino-4-(4-fluorophenyl)-6-phenylpyrimidine-5-carbonitrile (18j). Yield: 62%. Mp 223-224ºC. ^{1}H NMR (300 MHz, $CDCl_3$), *δ* (ppm): 8.02-7.92 (m, 4H), 7.57-7.51 (m, 3H), 7.25-7.19 (m, 2H), 5.60 (brs, 2H).^{13}C NMR (75 MHz, $CDCl_3$) δ (ppm): 115.67, 115.96, 118.02, 128.66, 128.83, 131.17, 131.31, 162.16, 166.28, 170.44, 171.84. HRMS (EI) *m/z*: calcd for $C_{17}H_{11}FN_4$ [M$^+$]: 290.0968, found: 290.0890.

2-amino-4-(4-bromophenyl)-6-phenylpyrimidine-5-carbonitrile (18k). Yield: 43%. Mp 218-219ºC. ^{1}H NMR (300 MHz, $CDCl_3$), *δ* (ppm): 7.97-7.90 (m, 2H), 7.88-7.80 (m, 2H), 7.71-7.64 (m, 2H), 7.59-7.50 (m, 3H), 5.69 (brs, 2H). MS (EI) *m/z*: 352 (96), 351 (100, M$^+$), 350 (98), 349 (90, M$^+$), 272 (22), 271 (91), 135 (21).

2-amino-4-(4-methoxyphenyl)-6-phenylpyrimidine-5-carbonitrile (18l). Yield: 61%. Mp 185-187ºC. ^{1}H NMR (300 MHz, $CDCl_3$), *δ* (ppm): 8.01-7.91 (m, 4H), 7.55-7.52 (m, 3H), 7.26-7.02 (m, 2H), 5.60 (brs, 2H), 3.88 (s, 3H).^{13}C NMR (75 MHz, $CDCl_3$) δ (ppm): 171.86, 170.79, 162.19, 136.26, 131.12, 130.74, 129.58, 128.82, 128.60, 128.41, 118.46, 114.02, 92.89, 55.44. HRMS (EI) *m/z*: calcd for $C_{18}H_{14}N_4O$ [M$^+$]: 302.1168, found: 302.1169.

2-amino-4-(4-hydroxyphenyl)-6-phenylpyrimidine-5-carbonitrile (18m). Yield: 69%. Mp 292-293ºC. ^{1}H NMR (300 MHz, DMSO-d_6), *δ* (ppm): 10.14 (brs, 1H), 7.84-7.82 (m, 6H), 7.55-7.53 (m, 3H), 6.89 (d, *J* = 8.68, 2H). HRMS (EI) *m/z*: calcd for $C_{17}H_{12}N_4O$ [M$^+$]: 288.1011, found: 288.0932.

2-amino-4-phenyl-6-(p-tolyl)pyrimidine-5-carbonitrile (18n). Yield: 73%. Mp 192-193ºC. ^{1}H NMR (300 MHz, $CDCl_3$), *δ* (ppm): 7.93 (m, 2H), 7.85 (d, *J* = 8.0 Hz, 2H), 7.54 (m, 3H), 7.33 (m, 2H), 5.73 (brs, 2H), 2.48 (s, 3H). ^{13}C NMR (75 MHz, $CDCl_3$) δ (ppm): 171.1, 170.8, 163.1, 141.3, 137.0, 134.1, 131.2, 129.3, 129.2, 128.7, 119.1, 91.2, 21.4. HRMS (ESI) *m/z*: calcd for $C_{18}H_{14}N_4$ [M+H]$^+$: 287.1296, found: 287.1291.

2-amino-4-(2,4-difluorophenyl)-6-phenylpyrimidine-5-carbonitrile (18o). Yield: 75%. Mp 152-156ºC. ^{1}H NMR (300 MHz, $CDCl_3$), *δ* (ppm): 7.98-7.95 (m, 2H), 7.57-7.53 (m, 4H), 7.06-7.00 (m, 2H), 5.69 (brs, 2H). MS (EI) *m/z*: 308.1 (M$^+$, 99), 307.1 (100), 299.1 (94), 272.0 (30), 163.0 (21).

2-amino-4-(2,4-dichlorophenyl)-6-phenylpyrimidine-5-carbonitrile (18p). Yield: 51%. Mp 178-180ºC. ^{1}H NMR (300 MHz, $CDCl_3$), δ (ppm): 7.99-7.97 (m, 2H), 7.57-7.41 (m, 4H), 7.41 (s, 2H), 5.69 (brs, 2H). HRMS (EI) *m/z*: calcd for $C_{17}H_{10}Cl_2N_4$ [M$^+$]: 340.0283, found: 340.0283.

2-amino-4-(2,4-dimethoxyphenyl)-6-phenylpyrimidine-5-carbonitrile (18q). Yield: 40%. Mp 187-188ºC. ^{1}H NMR (300 MHz, $CDCl_3$), δ (ppm): 7.83-7.78 (m, 3H), 7.55-7.53 (m, 2H), 7.33 (d, *J* = 8.4Hz, 1H), 6.71 (d, *J* = 1.8Hz, 1H), 6.64 (dd, *J* = 8.4, 2.1Hz, 1H), 3.86 (s, 3H), 3.82 (s, 3H). MS (EI) *m/z*: 332.1 (M$^+$, 100), 331.0 (92), 315.0 (27), 301,0 (37), 289.0 (29).

2-amino-4-(3,5-difluorophenyl)-6-phenylpyrimidine-5-carbonitrile (18r). Yield: 61%. Mp 225-226ºC. ^{1}H NMR (300 MHz, DMSO-*d*$_6$), δ (ppm): 10.42 (brs, 2H), 8.14 – 8.08 (m, 2H), 7.71 (d, *J* = 2.2 Hz, 2H), 7.56 – 7.48 (m, 2H), 7.44 – 7.37 (m, 1H), 7.01 (t, *J* = 2.2 Hz, 1H). HRMS (EI) *m/z*: calcd for $C_{17}H_{10}F_2N_4$ [M$^+$]: 308.0874, found: 308.0876.

2-amino-4-(3,5-dichlorophenyl)-6-phenylpyrimidine-5-carbonitrile (18s). Yield: 40%. Mp 181-183ºC. ^{1}H NMR (300 MHz, DMSO-*d*$_6$), δ (ppm): 8.03 (s, 2H), 7.92-7.87 (m, 5H), 7.57-7.55 (m, 3H). MS (EI) *m/z*: 340.01 (M$^+$, 100), 305.04 (95), 288.0 (39), 152.0 (42).

2-amino-4-(3,5-dimethoxyphenyl)-6-phenylpyrimidine-5-carbonitrile (18t). Yield: 55%. Mp 219-221ºC. ^{1}H NMR (300 MHz, DMSO-*d*$_6$), δ (ppm): 7.90 (s, 2H), 7.89-7.80 (m, 2H), 7.67-7.47 (m, 3H), 7.20-7.11 (m, 2H), 6.83-6.64 (m, 1H), 3.79 (s, 6H). HRMS (EI) *m/z*: calcd for $C_{19}H_{16}N_4O_2$ [M$^+$]: 332.1273 found: 332.1273.

2-amino-4-(benzo[*d*][1,3]dioxol-5-yl)-6-phenylpyrimidine-5-carbonitrile (18u). Yield: 68%. Mp 243-245ºC. ^{1}H NMR (300 MHz, $CDCl_3$), δ (ppm): 7.97-7.87 (m, 2H), 7.63-7.43 (m, 5H), 6.95 (d, *J* = 8.2 Hz, 1H), 6.06 (s, 2H), 5.59 (brs, 2H). HRMS (EI) *m/z*: calcd for $C_{18}H_{12}N_4O_2$ [M$^+$]: 316.0960, found: 316.0959.

2-amino-4-phenyl-6-(3,4,5-trimethoxyphenyl)pyrimidine-5-carbonitrile (18v). Yield: 50%. Mp 185-186ºC. ^{1}H NMR (300 MHz, DMSO-*d*$_6$), δ (ppm): 7.88-7.87 (m, 4H), 7.56-7.53 (m, 3H), 7.22 (s, 2H), 3.83 (s, 6H), 3.74 (s, 3H). ^{13}C NMR (75 MHz, $CDCl_3$) δ (ppm): 170.71, 170.08, 162.62, 152.55, 139.67, 136.54, 131.54, 130.79, 128.75, 128.29, 118.75, 106.55, 91.00, 60.19, 56.07 HRMS (EI) *m/z*: calcd for $C_{20}H_{18}N_4O_3$ [M$^+$]: 362.1379, found: 362.1381.

2-amino-4-phenyl-6-(2,4,6-trifluorophenyl)pyrimidine-5-carbonitrile (18w). Yield: 41%. Mp 226-227ºC. ^{1}H NMR (300 MHz, DMSO-*d*$_6$), δ (ppm): 7.63-7.60 (m, 2H), 7.45-7.39 (m, 3H), 7.27 (dd, *J* = 8.7 Hz, 2H), 5.7 (brs, 2H). MS (EI) *m/z*: 328.1 (40), 327.1 (16), 326.1 (M$^+$, 16), 307.1 (16), 197.1 (100)

2-amino-4-(furan-2-yl)-6-phenylpyrimidine-5-carbonitrile (18x). Yield: 41%. Mp 164-165ºC. ^{1}H NMR (300 MHz, $CDCl_3$), δ (ppm): 7.97-7.83 (m, 2H), 7.79 (d, *J* = 3.7 Hz, 1H), 7.73 (d, *J*

= 1.7 Hz, 1H), 7.65-7.41 (m, 3H), 6.64 (dd, *J* = 3.7, 1.7 Hz, 1H), 5.73 (brs, 2H). HRMS (EI) *m/z*: calcd for $C_{15}H_{10}N_4O$ [M^+]: 262.0855, found 262.0854.

2-amino-4-phenyl-6-(thiophen-2-yl)pyrimidine-5-carbonitrile (18y). Yield: 63%. Mp 163-165ºC. ^{1}H NMR (300 MHz, $CDCl_3$), *δ* (ppm): 8.48 (dd, *J* = 4.0, 1.0 Hz, 1H), 7.92-7.85 (m, 2H), 7.63-7.60 (m, 1H), 7.56-7.52 (m, 3H), 7.21 (dd, *J* = 5.1, 4.0 Hz, 1H), 5.54 (brs, 2H). HRMS (EI) *m/z*: calcd for $C_{15}H_{10}N_4S$ [M^+]: 278.0626, found: 278.0627.

2-amino-4-(furan-3-yl)-6-phenylpyrimidine-5-carbonitrile (18z). Yield: 74%. Mp 182-184ºC. ^{1}H NMR (300 MHz, $CDCl_3$), *δ* (ppm): 8.49-8.47 (m, 1H), 7.90-7.87 (m, 2H), 7.62-7.59 (m, 1H), 7.56-7.51 (m, 3H), 7.23-7.19 (m, 1H), 5.52 (brs, 2H). HRMS (EI) *m/z* calcd for $C_{15}H_{10}N_4O$ [M^+]: 262.0855 found: 262.0857.

2-amino-4-phenyl-6-(thiophen-3-yl)pyrimidine-5-carbonitrile (18aa). Yield: 48%. Mp 181-182ºC. ^{1}H NMR (300 MHz, DMSO-*d*6), *δ* (ppm): 8.64 (dd, *J* = 1.6, 1.0 Hz, 1H), 7.91-7.88 (m, 3H), 7.55-7.51 (m, 3H), 7.44-7.41 (m, 1H), 5.60 (bs, 2H). ^{13}C NMR (75 MHz, DMSO-*d*6) δ (ppm): 171.1, 164.4, 162.9, 138.1, 136.6, 130.9, 130.1, 128.8, 128.4, 127.8, 127.1, 119.1, 89.8. HRMS (ESI) *m/z*: calcd for $C_{15}H_{10}N_4S$ [M+H]$^+$: 279.0704, found: 279.0699.

2-amino-4-phenyl-6-(pyridin-4-yl)pyrimidine-5-carbonitrile (18ab). Yield: 49%. Mp 266-267ºC. ^{1}H NMR (300 MHz, DMSO-*d*6), *δ* (ppm): 8.8-8.78 (m, 2H), 8.03 (s, 2H), 7.88-7.85 (m, 2H), 7.80-7.78 (m, 2H), 7.59-7.54 (m, 3H). ^{13}C NMR (75 MHz, $CDCl_3$) δ (ppm): 168.77, 162.77, 150.03, 143.70, 136.23, 136.02, 131.03, 128.70, 128.43, 122.80. MS (EI) *m/z*: 273.1 (M^+, 98), 271.9 (100).

2-amino-4-phenyl-6-(pyridin-3-yl)pyrimidine-5-carbonitrile (18ac). Yield: 68%. Mp 243-244°C. ^{1}H NMR (300 MHz, DMSO-*d*6), *δ* (ppm): 9.17 (d, *J* = 1.6 Hz, 1H), 8.75-8.70 (m, 1H), 8.28-8.22 (m, 1H), 8.10-8.01 (m, 2H), 7.90-7.86 (m, 2H), 7.62-7.55 (m, 4H).^{13}C NMR (75 MHz, DMSO-*d*6) δ (ppm): 187.9, 170.6, 168.5, 162.9, 151.5, 149.2, 136.4, 132.5, 131.1, 128.8, 128.5, 123.5, 118.4, 91.5. HRMS (ESI) *m/z*: calcd for $C_{16}H_{11}N_5$ [M+H]$^+$: 274.1093, found: 274.1087.

2-amino-4-cyclopentyl-6-phenylpyrimidine-5-carbonitrile (18ad). Yield: 67%. Mp 185-186ºC. ^{1}H NMR (300 MHz, $CDCl_3$), *δ* (ppm): 7.90-7.87 (m, 2H), 7.53-7.41 (m, 3H), 5.46 (brs, 2H), 2.27-1.52 (m, 9H). HRMS (EI) *m/z*: calcd for $C_{16}H_{16}N_4$ [M^+]: 264.1375, found: 264.1303.

2-amino-4-cyclohexyl-6-phenylpyrimidine-5-carbonitrile (18ae). Yield: 46%. Mp 186-187 °C . ^{1}H NMR (300 MHz, $CDCl_3$), *δ* (ppm): 7.98-7.87 (m, 2H), 7.50-7.22 (m, 3H), 5.62 (brs, 2H), 3.11-2.99 (m, 1H), 1.85-1.20 (m, 10H).^{13}C NMR (75 MHz, $CDCl_3$) δ (ppm): 180.4, 162.3, 136.5, 172.5, 169.5, 130.9, 128.4, 117.2, 93.7, 44.3, 30.9, 25.8, 25.6. HRMS (ESI) *m/z*: calcd for $C_{17}H_{18}N_4$ [M+H]$^+$: 279.1610, found: 279.1604.

2-amino-4-(naphthalen-2-yl)-6-phenylpyrimidine-5-carbonitrile (18af). Yield: 44%. Mp 199-201ºC. ^1H NMR (300 MHz, $CDCl_3$), *δ* (ppm): 8.51 (s, 1H), 8.00-7.96 (m, 6H), 7.60-7.54 (m, 5H), 5.68 (brs, 2H). HRMS (EI) *m/z*: calcd for $C_{21}H_{14}N_4$ [M^+]: 322.1218, found: 322.1231.

4-([1,1'-biphenyl]-4-yl)-2-amino-6-phenylpyrimidine-5-carbonitrile (18ag). Yield: 46%. Mp 219-221ºC. ^1H NMR (300 MHz, DMSO-d_6), *δ* (ppm): 8.00 (d, *J* = 7.8 Hz, 2H), 7.95-7.81 (m, 5H), 7.77 (d, *J* = 7.5 Hz, 2H), 7.62–7.35 (m, 5H), 5.69 (brs, 2H). MS (EI) *m/z*: 348.3 (M^+, 79), 347.3 (100), 330.2 (8).

2-amino-4,6-bis(3-chlorophenyl)pyrimidine-5-carbonitrile (18ah). Yield: 49%. Mp 241-242ºC. ^1H NMR (300 MHz, DMSO-d_6), *δ* (ppm): 8.05 (s, 2H), 7.99-7.78 (m, 4H), 7.77-7.50 (m, 4H). MS (EI) *m/z*: 341.0 (M^+, 85), 306.0 (100).

2-amino-4-(3-chlorophenyl)-6-(3,5-dichlorophenyl)pyrimidine-5-carbonitrile (18ai). Yield: 46%. Mp 274-275 °C . ^1H NMR (300 MHz, DMSO-d_6), *δ* (ppm): 8.11 (s, 2H), 7.93-7.84 (m, 5H), 7.66-7.59 (m, 2H).^{13}C NMR (75 MHz, $CDCl_3$) δ (ppm): 169.1, 167.9, 162.8, 139.6, 138.3, 134.3, 133.3, 130.9, 130.6, 130.4, 128.6, 127.6, 127.5, 118.0, 91.5. HRMS (ESI) *m/z*: calcd for $C_{17}H_9Cl_3N_4$ $[M+H]^+$: 374.9971, found: 374.9966.

2-amino-4-(benzo[*d*][1,3]dioxol-5-yl)-6-(3-chlorophenyl)pyrimidine-5-carbonitrile (18aj). Yield: 55%. Mp 262-263ºC. ^1H NMR (300 MHz, $CDCl_3$), *δ* (ppm): 7.90-7.81 (m, 2H), 7.62-7.41 (m, 4H), 6.95 (dd, *J* = 8.2, 0.8 Hz, 1H), 6.06 (s, 2H), 5.58 (brs, 2H). MS (EI) *m/z*: 350.1 (M^+, 100), 315.1 (46), 146.0 (32).

2-amino-4-(benzo[d][1,3]dioxol-5-yl)-6-(4-fluorophenyl)pyrimidine-5-carbonitrile (18ak). Yield: 49%. Mp 242-243ºC. ^1H NMR (300 MHz, $CDCl_3$), *δ* (ppm): 8.07-7.91 (m, 2H), 7.58 (dd, *J* = 8.2, 1.8 Hz, 1H), 7.44 (d, *J* = 1.8 Hz, 1H), 7.21 (dd, *J* = 8.7 Hz, 2H), 6.95 (d, *J* = 8.2 Hz, 1H), 6.06 (s, 2H), 5.56 (brs, 2H). HRMS (EI) *m/z*: calcd for $C_{18}H_{11}FN_4O_2$ [M^+]: 334.0866, found: 334.0859.

2-amino-4,6-bis(4-methoxyphenyl)pyrimidine-5-carbonitrile (18al). Yield: 66%. Mp 320-322ºC. ^1H NMR (300 MHz, DMSO-d_6), *δ* (ppm): 7.94-7.83 (m, 4H), 7.77 (s, 2H), 7.15-7.03 (m, 4H), 3.84 (s, 6H). HRMS (EI) *m/z*: calcd for $C_{19}H_{16}N_4O_2$ [M^+]: 332.1273, found 332.1259.

2-amino-4-(4-fluorophenyl)-6-(furan-2-yl)pyrimidine-5-carbonitrile (18am). Yield: 58%. Mp 268-269ºC. ^1H NMR (300 MHz, $CDCl_3$), *δ* (ppm): 8.00-7.88 (m, 2H), 7.78 (d, *J* = 3.6 Hz, 1H), 7.73 (d, *J* = 1.5 Hz, 1H), 7.26-7.18 (m, 2H), 6.65 (dd, *J* = 3.6, 1.7 Hz, 1H), 5.61 (brs, 2H). HRMS (EI) *m/z*: calcd for $C_{15}H_9FN_4O$ [M^+]: 280.0760, found: 280.0765.

2-(methylamino)-4,6-diphenylpyrimidine-5-carbonitrile (19a). Yield: 57%. Mp 214-216 °C . ^1H NMR (300 MHz, $CDCl_3$), *δ* (ppm): 8.05-7.89 (m, 4H), 7.54-7.52 (m, 6H), 5.86 (bs, 1H), 3.11 (d, *J* = 5.1 Hz, 3H).^{13}C NMR (75 MHz, $CDCl_3$) δ (ppm): 171.0, 161.7, 136.3, 131.0, 128.9, 128.6, 118.5, 91.7, 28.1. HRMS (ESI) *m/z*: calcd for: $C_{18}H_{14}N_4$ $[M+H]^+$: 287.1296, found: 287.1291.

2-(ethylamino)-4,6-diphenylpyrimidine-5-carbonitrile (19b). Yield: 75%. Mp 178-180 °C. ^{1}H NMR (300 MHz, $CDCl_3$), *δ* (ppm): 8.02-7.90 (m, 4H), 7.50-7.25 (m, 6H), 5.86 (t, *J* = 4.63, 1H), 3.58 (m, 2H), 1.26 (t, *J* = 7.2, 3H).^{13}C NMR (75 MHz, $CDCl_3$) δ (ppm): 171.0, 161.1, 136.3, 130.8, 128.9, 128.4, 118.5, 91.7, 36.3, 14.6. HRMS (EI) *m/z*: calcd for $C_{19}H_{16}N_4$ [M^+]: 300.1375, found: 300.3567.

4,6-diphenyl-2-(phenylamino)pyrimidine-5-carbonitrile (19c). Yield: 73%. Mp 165-167 °C. ^{1}H NMR (300 MHz, $CDCl_3$), *δ* (ppm): 8.04-8.02 (m, 4H), 7.76-7.68 (m, 3H), 7.60-7.55 (m, 6H), 7.38 (dd, *J* = 7.5 Hz, 2H), 7.13 (dd, *J* = 7.5 Hz, 1H).^{13}C NMR (75 MHz, $CDCl_3$) δ (ppm): 171.1, 158.9, 138.0, 136.0, 131.2, 128.9, 128.8, 128.5, 123.8, 119.8, 118.0, 93.9. HRMS (ESI) *m/z*: calcd for $C_{23}H_{16}N_4$ [M+H]$^+$: 349.1453, found: 349.1448.

4-(2-fluorophenyl)-2-(methylamino)-6-phenylpyrimidine-5-carbonitrile (19d). Yield: 52%. Mp 184-186ºC. ^{1}H NMR (300 MHz, DMSO-d_6), *δ* (ppm): 8.47 (bs, 1H), 7.95-7.83 (m, 2H), 7.72-7.59 (m, 5H), 7.45-7.37 (m, 2H), 2.96-2.89 (m, 3H). HRMS (EI) *m/z*: calcd for $C_{18}H_{13}FN_4$ [M^+]: 304.1124, found: 304.1139.

4-(2-fluorophenyl)-6-phenyl-2-(phenylamino)pyrimidine-5-carbonitrile (19e). Yield: 67%. Mp 132-134ºC. ^{1}H NMR (300 MHz, $CDCl_3$), *δ* (ppm): 10.70 (brs, 1H), 8.04-7.92 (m, 2H), 7.88-7.57 (m, 6H), 7.52-7.26 (m, 4H), 7.17-7.00 (m, 1H). HRMS (EI) *m/z*: calcd for $C_{23}H_{15}FN_4$ [M^+]: 366.1281, found: 366.1281.

4-(2-chlorophenyl)-2-(methylamino)-6-phenylpyrimidine-5-carbonitrile (19f). Yield: 75%. Mp 198-200ºC. ^{1}H NMR (300 MHz, $CDCl_3$), *δ* (ppm): 8.48 (s, 1H), 8.01-7.82 (m, 2H), 7.68-7.47 (m, 7H), 3.00-2.86 (m, 3H). HRMS (EI) *m/z*: calcd for $C_{18}H_{13}ClN_4$ [M^+]: 320.0829, found: 320.0823.

4-(2-chlorophenyl)-6-phenyl-2-(phenylamino)pyrimidine-5-carbonitrile (19g). Yield: 52%. Mp 119-121ºC. ^{1}H NMR (300 MHz, $CDCl_3$), *δ* (ppm): 8.13-8.05 (m, 2H), 7.70 (d, *J* = 8.0 Hz, 2H), 7.65-7.50 (m, 6H), 7.52-7.40 (m, 2H), 7.37 (dd, *J* = 7.8 Hz, 2H), 7.13 (dd, *J* = 7.4 Hz, 1H). MS (EI) *m/z*: 383.0 (M^+, 48), 382.0 (M^+, 65), 381.0 (M^+, 100), 172.0 (39).

4-(2-methoxyphenyl)-2-(methylamino)-6-phenylpyrimidine-5-carbonitrile (19h). Yield: 39%. Mp 229-230°C. ^{1}H NMR (300 MHz, DMSO-d_6), *δ* (ppm): 8.32-8.30 (m, 1H), 7.94-7.90 (m, 1H), 7.85-7.81 (m, 1H), 7.57-7.35 (m, 5H), 7.28 (d, *J* = 4.1 Hz, 1H), 7.20-7.04 (m, 1H), 3.81 (s, 3H), 2.95 (d, *J* = 4.7 Hz, 3H).^{13}C NMR (75 MHz, DMSO-d_6) δ (ppm): 171.2, 168.7, 162.0, 156.6, 136.7, 131.8, 31.1, 130.2, 129.9, 128.8, 126.1, 120.6, 118.1, 111.9, 93.3, 55.7, 28.1. HRMS (ESI) *m/z*: calcd for $C_{19}H_{16}N_4O$ [M+H]$^+$: 317.1402, found: 317.1397.

4-(2-methoxyphenyl)-6-phenyl-2-(phenylamino)pyrimidine-5-carbonitrile (19i). Yield: 47%. Mp 199-201ºC. ^{1}H NMR (300 MHz, DMSO-d_6), *δ* (ppm): 8.79 – 8.73 (m, 2H), 8.34 – 8.28 (m,

2H), 8.17 (dd, *J* = 8.4, 1.3 Hz, 1H), 7.56 – 7.48 (m, 2H), 7.44 – 7.37 (m, 2H), 7.23 (td, *J* = 7.5, 1.3 Hz, 1H), 7.20 – 7.13 (m, 2H), 6.93 (tt, *J* = 7.1, 1.1 Hz, 1H), 6.85 (dd, *J* = 7.7, 1.3 Hz, 1H), 5.54 (brs, 1H) 3.94 (s, 3H). HRMS (EI) *m/z*: calcd for $C_{24}H_{18}N_4O$ [M^+]: 378.1481, found: 378.1476.

4-(3-fluorophenyl)-2-(methylamino)-6-phenylpyrimidine-5-carbonitrile (19j). Yield: 45%. Mp 237-239ºC. ^{1}H NMR (300 MHz, DMSO-*d*6), *δ* (ppm): 8.32 – 8.26 (m, 2H), 8.18 – 8.12 (m, 1H), 8.06 (s, 1H), 7.74 (dd, *J* = 8.6, 7.8 Hz, 1H), 7.56 – 7.48 (m, 2H), 7.44 – 7.37 (m, 1H), 7.06 – 7.00 (m, 1H), 5.56 (brs, 1H), 3.30 (d, *J* = 2.7 Hz, 3H). HRMS (EI) *m/z*: calcd for $C_{18}H_{13}FN_4$ [M^+]: 304.1124, found: 304.1112.

4-(3-fluorophenyl)-6-phenyl-2-(phenylamino)pyrimidine-5-carbonitrile (19k). Yield: 44%. Mp 176-177ºC. ^{1}H NMR (300 MHz, DMSO-*d*6), *δ* (ppm): 10.66 (s, 1H), 8.01-7.93 (m, 2H), 7.87-7.74 (m, 4H), 7.71-7.57 (m, 4H), 7.56-7.42 (m, 1H), 7.35 (dd, *J* = 7.9 Hz, 2H), 7.07 (dd, *J* = 7.4 Hz, 1H). HRMS (EI) *m/z*: calcd for $C_{23}H_{15}FN_4$ [M^+]: 366.1281, found: 366.1266.

4-(3-chlorophenyl)-2-(methylamino)-6-phenylpyrimidine-5-carbonitrile (19l). Yield: 69%. Mp 214-215ºC. ^{1}H NMR (300 MHz, $CDCl_3$), *δ* (ppm): 8.89-7.70 (m, 4H), 7.89-7.35 (m, 5H), 5.76 (brs, 1H), 3.16 (d, *J* = 5.1 Hz, 3H). HRMS (EI) *m/z*: calcd for $C_{18}H_{13}ClN_4$ [M^+]: 320.0829, found: 320.0824.

4-(3-chlorophenyl)-2-(ethylamino)-6-phenylpyrimidine-5-carbonitrile (19m). Yield: 46%. Mp 179-180ºC. ^{1}H NMR (300 MHz, $CDCl_3$), *δ* (ppm): 9.14-8.99 (m, 1H), 8.82-8.66 (m, 1H), 8.57 (dd, *J* = 6.1 Hz, 1H), 8.41-8.20 (m, 1H), 7.99-7.82 (m, 2H), 7.71-7.51 (m, 4H), 3.53-3.37 (m, 2H), 1.18 (t, *J* = 7.1 Hz, 3H). HRMS (EI) *m/z*: calcd for $C_{19}H_{15}ClN_4$: 334.0985, found: 334.0823.

4-(3-chlorophenyl)-6-phenyl-2-(phenylamino)pyrimidine-5-carbonitrile (19n). Yield: 43%. Mp 214-215ºC. ^{1}H NMR (300 MHz, $CDCl_3$), *δ* (ppm): 8.07-7.91 (m, 3H), 7.70 (d, *J* = 7.7 Hz, 2H), 7.64-7.45 (m, 7H), 7.40 (dd, *J* = 8.0 Hz, 2H), 7.15 (dd, *J* = 7.8 Hz, 1H). HRMS (EI) *m/z*: calcd for $C_{23}H_{15}ClN_4$ [M^+]: 382.0985, found: 382.1006.

4-(3-hydroxyphenyl)-2-(methylamino)-6-phenylpyrimidine-5-carbonitrile (19o). Yield: 51%. Mp 256-258ºC. ^{1}H NMR (300 MHz, DMSO-*d*6), *δ* (ppm): 9.80 (s, 1H), 8.36-8.26 (m, 1H), 7.94 (dd, *J* = 7.5, 2.1 Hz, 1H), 7.85 (dd, *J* = 7.5, 2.1 Hz, 1H), 7.59-7.51 (m, 3H), 7.38-7.22 (m, 3H), 7.02-6.93 (m, 1H), 2.96 (d, *J* = 4.7 Hz, 3H). HRMS (EI) *m/z*: calcd for $C_{18}H_{14}N_4O$ [M^+]: 302.1168, found 302.1167.

4-(3-hydroxyphenyl)-6-phenyl-2-(phenylamino)pyrimidine-5-carbonitrile (19p). Yield: 54%. Mp 205-207ºC. ^{1}H NMR (300 MHz, DMSO-*d*6), *δ* (ppm): 10.60 (dd, 6.2 Hz, 1H), 9.88 (dd, *J* = 5.8 Hz, 1H), 8.08-7.72 (m, 4H), 7.73-7.22 (m, 8H), 7.18-6.92 (m, 2H). HRMS (EI) *m/z*: calcd for $C_{23}H_{16}N_4O$ [M^+]: 364.1324, found: 364.1309.

4-(3-methoxyphenyl)-2-(methylamino)-6-phenylpyrimidine-5-carbonitrile (19q). Yield: 58%. Mp 197-198ºC. ^{1}H NMR (300 MHz, DMSO-*d*$_6$), *δ* (ppm): 8.38 (d, *J* = 4.5 Hz, 1H), 8.00-7.81 (m, 2H), 7.65-7.39 (m, 6H), 7.26-7.08 (m, 1H), 3.84 (s, 3H), 2.96 (d, *J* = 4.6 Hz, 3H). HRMS (EI) *m/z*: calcd for $C_{19}H_{16}N_4O$ [M$^+$]: 316.1324, found: 316.1336.

4-(3-methoxyphenyl)-6-phenyl-2-(phenylamino)pyrimidine-5-carbonitrile (19r). Yield: 67%. Mp 135-137ºC. ^{1}H NMR (300 MHz, DMSO-*d*$_6$), *δ* (ppm): 10.62 (s, 1H), 8.04-7.92 (m, 2H), 7.83 (d, *J* = 8.5 Hz, 2H), 7.67-7.48 (m, 6H), 7.36 (t, *J* = 7.8 Hz, 2H), 7.23-7.15 (m, 1H), 7.07 (dd, *J* = 7.8, 1H), 3.85 (s, 3H). HRMS (EI) *m/z*: calcd for $C_{24}H_{18}N_4O$ [M$^+$]: 378.1481, found: 378.1490.

4-(3-cyanophenyl)-2-(methylamino)-6-phenylpyrimidine-5-carbonitrile (19s). Yield: 43%. Mp 247-248ºC. ^{1}H NMR (300 MHz, DMSO-*d*$_6$), *δ* (ppm): 8.55-8.41 (m, 1H), 8.42-8.13 (m, 2H), 8.12-8.02 (m, 1H), 7.96 (dd, *J* = 7.5, 2.1 Hz, 1H), 7.87 (dd, *J* = 7.5, 2.1 Hz, 1H), 7.82-7.76 (m, *J* = 7.9, 2.7 Hz, 1H), 7.64-7.50 (m, 3H), 2.96 (d, *J* = 4.7 Hz, 3H).^{13}C NMR (75 MHz, DMSO-*d*$_6$) δ (ppm): 170.5, 169.3, 162.2, 138.3, 137.2, 135.1, 134.2, 134.1, 133.2, 131.8, 130.5, 129.5, 129.2, 119.0, 112.2, 91.4, 28.6. HRMS (EI) *m/z*: calcd for $C_{19}H_{13}N_5$ [M$^+$]: 311.3400, found: 311.3394.

4-(3-cyanophenyl)-2-(ethylamino)-6-phenylpyrimidine-5-carbonitrile (19t). Yield: 40%. Mp 175-176ºC. ^{1}H NMR (300 MHz, $CDCl_3$), *δ* (ppm): 8.58 (s, 1H), 8.34 (dd, *J* = 15.8 , 1.0 Hz, 1H), 8.21 (dd, *J* = 15.8, 7.2 Hz, 1H), 8.07 (d, *J* = 7.2 Hz, 1H), 7.91 (dd *J* = 18.6, 6.9 Hz, 2H), 7.86-7.72 (m, 1H), 7.69-7.51 (m, 3H), 3.52-3.39 (m, 2H), 1.17 (t, *J* = 7.1 Hz, 3H).^{13}C NMR (75 MHz, DMSO-*d*$_6$) δ (ppm): 170.9, 169.2, 161.5, 138.3, 137.2, 135.1, 134.2, 134.1, 133.2, 131.8, 130.5, 129.5, 129.1, 119.0, 112.2, 91.5, 36.5, 15.0. HRMS (EI) *m/z*: calcd for $C_{20}H_{15}N_5$: 325.1327 found: 325.1328.

4-(3-cyanophenyl)-6-phenyl-2-(phenylamino)pyrimidine-5-carbonitrile (19u). Yield: 46%. Mp 211-212ºC. ^{1}H NMR (300 MHz, DMSO-*d*$_6$), *δ* (ppm): 10.70 (s, 1H), 8.41 (s, 1H), 8.33-8.21 (m, 1H), 8.16-8.05 (m, 1H), 8.04-7.93 (m, 2H), 7.90-7.73 (m, 3H), 7.68-7.57 (m, 3H), 7.35 (dd, *J* = 7.9 Hz, 2H), 7.07 (dd, *J* = 7.3 Hz, 1H). HRMS (EI) *m/z*: calcd for: $C_{24}H_{15}N_5$ [M$^+$]: 373.1327, found: 373.1325.

4-(4-fluorophenyl)-2-(methylamino)-6-phenylpyrimidine-5-carbonitrile (19v). Yield: 47%. Mp 250-251ºC. ^{1}H NMR (300 MHz, $CDCl_3$), *δ* (ppm): 7.95-7.67 (m, 4H), 7.42-7.36 (m, 3H), 7.04 (t, *J* = 8.6 Hz, 2H), 5.65 (bs, 1H), 2.96 (t, *J* = 4.3 Hz, 3H). HRMS (EI) *m/z*: calcd for $C_{18}H_{13}FN_4$ [M$^+$]: 304.1124, found: 304.1122.

4-(4-fluorophenyl)-6-phenyl-2-(phenylamino)pyrimidine-5-carbonitrile (19w). Yield: 65%. Mp 188-190ºC. ^{1}H NMR (300 MHz, $CDCl_3$), *δ* (ppm): 8.09-8.00 (m, 4H), 7.79-7.55 (m, 6H), 7.38 (t, *J* = 7.91 Hz, 2H), 7.27-7.20 (m, 2H), 7.14 (t, *J* = 7.41 Hz, 1H). HRMS (EI) *m/z*: calcd for $C_{23}H_{15}FN_4$ [M$^+$]: 366.1281, found: 366.1291.

4-(4-bromophenyl)-2-(methylamino)-6-phenylpyrimidine-5-carbonitrile (19x). Yield: 56%. Mp 230-231ºC. ^{1}H NMR (300 MHz, $CDCl_3$), *δ* (ppm): 8.17-7.73 (m, 4H), 7.76-7.42 (m, 5H), 5.76 (brs, 1H), 3.14 (s, 3H). MS (EI) *m/z*: 366.2 ($[M+H]^+$, 99), 365.2 (M^+, 85), 364.2 ($[M+H]^+$, 100), 363.2 (M^+, 67), 285.2 (61), 256.1 (43).

4-(4-bromophenyl)-6-phenyl-2-(phenylamino)pyrimidine-5-carbonitrile (19y). Yield: 44%. Mp 205-207ºC. ^{1}H NMR (300 MHz, $CDCl_3$), *δ* (ppm): 8.07-7.88 (m, 4H), 7.76-7.64 (m, 4H), 7.65-7.52 (m, 3H), 7.43-7.34 (m, 2H), 7.22-7.11 (m, 1H). MS (EI) *m/z*: 428.1 ($[M+H]^+$, 99), 427.0 (M^+, 85), 426.0 ($[M+H]^+$, 100), 425.0 (M^+, 67), 347.0 (17), 172.0 (15).

4-(4-hydroxyphenyl)-2-(methylamino)-6-phenylpyrimidine-5-carbonitrile (19z). Yield: 75%. Mp 307-308 °C. ^{1}H NMR (300 MHz, DMSO-d_6), *δ* (ppm): 10.15 (bs, 1H), 8.23 (bs, 1H), 7.92-7.78 (m, 4H), 7.56-7.52 (m, 3H), 6.93-6.88 (m, 2H), 2.95 (d, *J* = 3.8 Hz, 3H) ^{13}C NMR (75 MHz, $CDCl_3$) δ (ppm): 170.3, 169.6, 161.5, 160.5, 137.0, 130.9, 129.0, 128.4, 127.4, 127.1, 119.3, 115.2, 89.8, 28.0. HRMS (ESI) *m/z*: calcd for $C_{18}H_{14}N_4O$ $[M+H]^+$: 303.1246, found: 303.1240.

4-(4-hydroxyphenyl)-6-phenyl-2-(phenylamino)pyrimidine-5-carbonitrile (19aa). Yield: 52%. Mp 286-287 °C. ^{1}H NMR (300 MHz, DMSO-d_6), *δ* (ppm): 10.51 (brs, 1H), 10.24 (s, 1H), 7.96-7.90 (m, 4H), 7.83-7.80 (d, *J* = 7.6 Hz, 2H), 7.60-7.57 (m, 3H), 7.34 (m, 2H), 7.04-7.01 (m, 1H), 6.95 (d, *J* = 7.6 Hz, 2H).^{13}C NMR (75 MHz, $CDCl_3$) δ (ppm): 170.7, 169.7, 160.6, 159.0, 139.3, 136.6, 131.1, 129.1, 129.0, 128.8, 128.6, 126.9, 123.2, 120.4, 118.8, 115.4, 92.2. HRMS (ESI) *m/z*: calcd for: $C_{23}H_{16}N_4O$ $[M+H]^+$: 365.1402, found: 365,1397.

4-(4-methoxyphenyl)-2-(methylamino)-6-phenylpyrimidine-5-carbonitrile (19ab). Yield: 73%. Mp 196-198ºC. ^{1}H NMR (300 MHz, $CDCl_3$), *δ* (ppm): 8.21-7.57 (m, 4H), 7.52-7.23 (m, 3H), 6.99-6.74 (m, 2H), 5.54 (brs, 1H), 3.72 (s, 3H), 2.98 (d, *J* = 4.7 Hz, 3H). HRMS (EI) *m/z*: calcd for $C_{19}H_{16}N_4O$ $[M]^+$: 316.1324, found: 316.1323.

4-(4-methoxyphenyl)-6-phenyl-2-(phenylamino)pyrimidine-5-carbonitrile (19ac). Yield: 60%. Mp 175-177ºC. ^{1}H NMR (300 MHz, $CDCl_3$), *δ* (ppm): 8.16-8.03 (m, 2H), 8.07-7.95 (m, 2H), 7.77-7.67 (m, 2H), 7.61-7.51 (m, 4H), 7.39 (dd, *J* = 7.9 Hz, 2H), 7.18-7.02 (m, 3H), 3.90 (s, 3H). HRMS (EI) *m/z*: calcd for $C_{24}H_{18}N_4O$ $[M]^+$: 378.1481, found: 378.1487.

2-(methylamino)-4-phenyl-6-(*p*-tolyl)pyrimidine-5-carbonitrile (19ad). Yield: 63%. Mp 191-192ºC. ^{1}H NMR (300 MHz, $CDCl_3$), *δ* (ppm): 8.30-7.67 (m, 4H), 7.66-7.41 (m, 3H), 7.33 (d, *J* = 7.9 Hz, 2H), 5.78 (brs, 1H), 3.12 (d, *J* = 4.9 Hz, 3H), 2.44 (s, 3H). HRMS (EI) *m/z*: calcd for $C_{19}H_{16}N_4$ $[M]^+$: 300.1375, found: 300.1380.

4-phenyl-2-(phenylamino)-6-(*p*-tolyl)pyrimidine-5-carbonitrile (19ae). Yield: 74%. Mp 147-149ºC. ^{1}H NMR (300 MHz, DMSO-d_6), *δ* (ppm): 10.54 (s, 1H), 8.01-7.92 (m, 2H), 7.88 (d, *J* = 8.1 Hz, 2H), 7.81 (dd, *J* = 7.8, 0.9 Hz, 2H), 7.68-7.52 (m, 3H), 7.40 (d, *J* = 7.9 Hz, 2H), 7.33 (dd, *J*

= 7.1Hz, 2H), 7.05 (td, *J* = 7.2, 1.1 Hz, 1H), 2.40 (s, 3H). HRMS (EI) *m/z*: calcd for $C_{24}H_{18}N_4$ $[M]^+$: 362.1530, found: 362.1520.

4-(2,4-difluorophenyl)-2-(methylamino)-6-phenylpyrimidine-5-carbonitrile (19af). Yield: 54%. Mp 221-223ºC. ^{1}H NMR (300 MHz, $CDCl_3$), δ (ppm): 8.06 (d, *J* = 5.6 Hz, 1H), 7.93 (d, *J* = 6.1 Hz, 1H), 7.75-7.45 (m, 3H), 7.11-6.93 (m, 3H), 5.84 (d, *J* = 22.5 Hz, 1H), 3.12 (dd, *J* = 17.6, 4.2 Hz, 3H). HRMS (EI) *m/z*: calcd for $C_{18}H_{12}F_2N_4$ $[M]^+$: 322.1030, found 322.1017.

4-(2,4-difluorophenyl)-6-phenyl-2-(phenylamino)pyrimidine-5-carbonitrile (19ag). Yield: 51%. Mp 192-194ºC. ^{1}H NMR (300 MHz, $CDCl_3$), δ (ppm): 7.66-7.47 (m, 2H), 7.39-7.18 (m, 6H), 7.10 (dd, *J* = 7.3 Hz, 1H), 6.97 (dd, *J* = 8.2 Hz, 2H), 6.85 (d, *J* = 7.6 Hz, 2H), 5.77 (brs, 1H). MS (EI) *m/z*: 384.1 (M^+, 75), 273.1 (100), 269.0 (48).

4-(3,5-difluorophenyl)-2-(methylamino)-6-phenylpyrimidine-5-carbonitrile (19ah). Yield: 69%. Mp 264-266ºC. ^{1}H NMR (300 MHz, $CDCl_3$), δ (ppm): 8.03 (d, *J* = 6.4 Hz, 1H), 7.90 (d, *J* = 5.8 Hz, 1H), 7.62-7.41 (m, 5H), 7.05-6.94 (m, 1H), 7.82-7.77 (m, 1H), 3.16 (d, *J* = 5.8 Hz, 3H). HRMS (EI) *m/z*: calcd for $C_{18}H_{12}F_2N_4$ $[M]^+$: 322.1030, found: 322.1032.

4-(3,5-difluorophenyl)-6-phenyl-2-(phenylamino)pyrimidine-5-carbonitrile (19ai). Yield: 45%. Mp 264-264ºC. ^{1}H NMR (300 MHz, $CDCl_3$), δ (ppm): 8.02 (d, *J* = 4.6 Hz, 2H), 7.81-7.51 (m, 8H), 7.40 (dd, *J* = 7.5 Hz, 2H), 7.16 (dd, *J* = 7.5 Hz, 1H), 7.03 (t, *J* = 8.0 Hz, 1H). HRMS (EI) *m/z*: calcd for $C_{23}H_{14}F_2N_4$ $[M]^+$: 384.1187, found: 384.1191.

4-(3,5-dichlorophenyl)-2-(methylamino)-6-phenylpyrimidine-5-carbonitrile (19aj). Yield: 62%. Mp 245-246ºC. ^{1}H NMR (300 MHz, $CDCl_3$), δ (ppm): 8.03 (d, *J* = 6.9 Hz, 1H), 7.94-7.85 (m, 2H), 7.79 (s, 1H), 7.63-7.44 (m, 4H), 5.91-5.68 (m, 1H), 3.15 (d, *J* = 5.7 Hz, 3H). HRMS (EI) *m/z*: calcd for $C_{18}H_{12}Cl_2N_4$: 354.0439, found: 354.0427.

4-(3,5-dichlorophenyl)-2-(ethylamino)-6-phenylpyrimidine-5-carbonitrile (19ak). Yield: 54%. Mp 192-193ºC. ^{1}H NMR (300 MHz, DMSO-d_6), δ (ppm): 10.42 (brs, 1H), 8.32 – 8.26 (m, 2H), 8.20 (s, 2H), 7.56 – 7.48 (m, 2H), 7.44 – 7.37 (m, 1H), 7.35 (t, *J* = 2.2 Hz, 1H), 3.46 (q, *J* = 7.6 Hz, 2H), 1.25 – 1.19 (m, 3H). MS (EI) *m/z*: 372.0 (33), 370.0 (60), 368.0 (M^+, 100), 352.7 (57).

4-(3,5-dichlorophenyl)-6-phenyl-2-(phenylamino)pyrimidine-5-carbonitrile (19l). Yield: 55%. Mp 179-181ºC. ^{1}H NMR (300 MHz, $CDCl_3$), δ (ppm): 7.95-7.79 (m, 2H), 7.72 (d, *J* = 1.9 Hz, 2H), 7.52 (d, *J* = 7.8 Hz, 2H), 7.43-7.34 (m, 4H), 7.24 (dd, *J* = 7.8 Hz, 2H), 7.01 (dd, *J* = 7.4 Hz, 1H). MS (EI) *m/z*: 420.0 (31), 416.9 (97), 416.0 (M^+, 100), 414.9 (95), 381.0 (56), 208.0 (49).

4-(3,5-dimethoxyphenyl)-2-(methylamino)-6-phenylpyrimidine-5-carbonitrile (19am). Yield: 64%. Mp 207-209ºC. ^{1}H NMR (300 MHz, DMSO-d_6), δ (ppm): 8.58-8.21 (m, 1H), 7.93 (d, *J* = 6.9 Hz, 1H), 7.84 (d, *J* = 6.9 Hz, 1H), 7.76-7.41 (m, 3H), 7.07 (d, *J* = 2.2 Hz, 1H), 6.98 (d, *J* = 2.1

Hz, 1H), 6.79-6.61 (m, 1H), 3.80 (s, 3H), 3.79 (s, 3H), 2.94 (d, *J* = 4.7 Hz, 3H). HRMS (EI) *m/z*: calcd for $C_{20}H_{18}N_4O_2$ $[M]^+$: 346.1430, found: 346.1416.

4-(3,5-dimethoxyphenyl)-6-phenyl-2-(phenylamino)pyrimidine-5-carbonitrile (19an). Yield: 59%. Mp 196-197ºC. ^{1}H NMR (300 MHz, DMSO-d_6), δ (ppm): 10.61 (s, 1H), 8.00-7.92 (m, 2H), 7.81 (d, *J* = 7.6 Hz, 2H), 7.65-7.51 (m, 3H), 7.34 (dd, *J* = 7.9 Hz, 2H), 7.17-6.99 (m, 3H), 6.74 (t, *J* = 2.3 Hz, 1H), 3.82 (s, 6H). HRMS (EI) *m/z*: calcd for $C_{25}H_{20}N_4O_2$: 408.1586, found: 408.1594.

4-(benzo[*d*][1,3]dioxol-5-yl)-2-(methylamino)-6-phenylpyrimidine-5-carbonitrile (19ao). Yield: 59%. Mp 214-216ºC. ^{1}H NMR (300 MHz, $CDCl_3$), δ (ppm): 8.04-7.84 (m, 2H), 7.72-7.39 (m, 5H), 6.95 (d, *J* = 8.2 Hz, 1H), 6.06 (s, 2H), 5.83 (s, 1H), 3.11 (d, *J* = 5.1 Hz, 3H). ^{13}C NMR (75 MHz, DMSO-d_6) δ (ppm): 170.2, 169.4, 161.5, 149.9, 147.5, 136.9, 131.0, 130.3, 128.9, 128.5, 124.0, 119.0, 109.1, 109.0, 101.9, 90.1, 28.0. HRMS (EI) *m/z*: calcd for $C_{19}H_{14}N_4O_2$ $[M]^+$: 330.1117, found: 330.1117.

4-(benzo[*d*][1,3]dioxol-5-yl)-2-(ethylamino)-6-phenylpyrimidine-5-carbonitrile (19ap). Yield: 43%. Mp 189-190ºC. ^{1}H NMR (300 MHz, $CDCl_3$), δ (ppm): 8.46-8.34 (m, 1H), 7.97-7.80 (m, 2H), 7.67-7.39 (m, 5H), 7.10 (dd, *J* = 8.1, 3.2 Hz, 2H), 6.14 (d, *J* = 1.4 Hz, 1H), 3.56-3.37 (m, 2H), 1.17 (t, *J* = 7.1 Hz, 3H). ^{13}C NMR (75 MHz, DMSO-d_6) δ (ppm): 171.1, 169.8, 161.5, 150.5, 148.1, 137.2, 131.4, 130.9, 129.6, 129.1, 124.4, 119.6, 109.6, 108.8, 102.5, 90.6, 36.4, 15.1. HRMS (EI) *m/z*: calcd for $C_{20}H_{16}N_4O_2$: 344.1273, found: 344.1284.

4-(benzo[*d*][1,3]dioxol-5-yl)-6-phenyl-2-(phenylamino)pyrimidine-5-carbonitrile (19aq). Yield: 59%. Mp 190-192ºC. ^{1}H NMR (300 MHz, $CDCl_3$), δ (ppm): 8.01-7.99 (m, 2H), 7.71-7.69 (m, 3H), 7.57-7.52 (m, 5H), 7.39 (dd, *J* =7.84, 2H), 7.13 (dd, *J* =7.41, 1H), 6.97 (d, *J* =8.17, 1H), 6.08 (s, 2H). ^{13}C NMR (75 MHz, $CDCl_3$) δ (ppm): 171.2. 170.0, 158.8, 150.3, 147.9, 138.0, 101.6, 93.2. HRMS (EI) *m/z*: calcd for $C_{24}H_{16}N_4O_2$ $[M]^+$: 392.1273, found 392.1270.

2-(methylamino)-4-phenyl-6-(3,4,5-trimethoxyphenyl)pyrimidine-5-carbonitrile (19ar). Yield: 49%. Mp 197-199ºC. ^{1}H NMR (300 MHz, $CDCl_3$), δ (ppm): 8.07-7.97 (m, 1H), 7.94-7.83 (m, 1H), 7.61-7.48 (m, 3H), 7.34 (s, 1H), 7.24-7.14 (m, 1H), 5.94 (s, 1H), 3.78 (s, 6H), 3.75 (s, 3H), 3.23-2.92 (m, 3H). HRMS (EI) *m/z*: calcd for $C_{21}H_{20}N_4O_3$ $[M]^+$: 376.1535, found: 376.1543.

4-phenyl-2-(phenylamino)-6-(3,4,5-trimethoxyphenyl)pyrimidine-5-carbonitrile (19as). Yield: 68%. Mp 192-194ºC. ^{1}H NMR (300 MHz, $CDCl_3$), δ (ppm): 8.05-7.98 (m, 2H), 7.77-7.69 (m, 2H), 7.64 (s, 1H), 7.60-7.52 (m, 3H), 7.45-7.40 (m, 4H), 7.14 (dd, *J* = 7.4 Hz, 1H), 3.80 (s, 6H), 3.78 (s, 3H). HRMS (EI) *m/z*: calcd for $C_{26}H_{22}N_4O_3$: 438.1692, found: 438.1703.

4-(furan-2-yl)-2-(methylamino)-6-phenylpyrimidine-5-carbonitrile (19at). Yield: 46%. Mp 193-195ºC. ^{1}H NMR (300 MHz, DMSO-d_6), δ (ppm): 8.42-8.23 (m, 1H), 8.13-8.01 (m, 1H), 7.94-

7.74 (m, 2H), 7.69-7.50 (m, 4H), 6.85-6.76 (m, 1H), 3.39-2.89 (m, 3H). HRMS (EI) *m/z*: calcd for $C_{16}H_{12}N_4O$: 276.1011, found: 276.1012.

4-(furan-2-yl)-6-phenyl-2-(phenylamino)pyrimidine-5-carbonitrile (19au). Yield: 48%. Mp 141-143ºC. ^{1}H NMR (300 MHz, DMSO-d_6), δ (ppm): 8.14 (s, 1H), 7.97-7.80 (m, 5H), 7.68-7.55 (m, 4H), 7.35 (dd, J = 7.3 Hz, 2H), 7.06 (dd, J = 7.1 Hz, 1H), 6.90-6.80 (m, 1H). HRMS (EI) *m/z*: calcd for $C_{21}H_{14}N_4O$: 338.1168, found: 338.1169.

2-(methylamino)-4-phenyl-6-(thiophen-2-yl)pyrimidine-5-carbonitrile (19av). Yield: 75%. Mp 198-199ºC. ^{1}H NMR (300 MHz, $CDCl_3$), δ (ppm): 8.31 (d, J = 3.8 Hz, 2H), 8.00-7.75 (m, 3H), 7.71-7.51 (m, 3H), 7.38-7.26 (m, 1H), 3.08-2.82 (m, 3H). HRMS (EI) *m/z*: calcd for $C_{16}H_{12}N_4S$: 292.0783, found: 292.0779.

4-phenyl-2-(phenylamino)-6-(thiophen-2-yl)pyrimidine-5-carbonitrile (19aw). Yield: 46%. Mp 178-179ºC. ^{1}H NMR (300 MHz, DMSO-d_6), δ (ppm): 10.55 (s, 1H), 8.42-8.31 (m, 1H), 8.08-7.96 (m, 1H), 7.95-7.77 (m, 4H), 7.64-7.53 (m, 3H), 7.44-7.29 (m, 3H), 7.13-7.02 (m, 1H). HRMS (EI) m*/z*: calcd for $C_{21}H_{14}N_4S$: 354.0939, found: 354.0944.

4-(furan-3-yl)-2-(methylamino)-6-phenylpyrimidine-5-carbonitrile (19ax). Yield: 65%. Mp 152-154ºC. ^{1}H NMR (300 MHz, DMSO-d_6), δ (ppm): 8.72-8.42 (m, 1H), 8.02-7.75 (m, 3H), 7.63-7.48 (m, 3H), 7.46-7.41 (m, 1H), 5.70 (brs, 1H), 3.16 (d, J = 5.2 Hz, 3H). HRMS (EI) *m/z*: calcd for $C_{16}H_{12}N_4O$: 276.101, found: 276.1003.

4-(furan-3-yl)-6-phenyl-2-(phenylamino)pyrimidine-5-carbonitrile (19ay). Yield: 52%. Mp 140-142ºC. ^{1}H NMR (300 MHz, DMSO-d_6), δ (ppm): 10.52 (s, 1H), 8.63 (s, 1H), 8.00-7.86 (m, 3H), 7.81 (d, J = 8.0 Hz, 2H), 7.70-7.55 (m, 3H), 7.37 (dd, J = 7.5 Hz, 2H), 7.20 (s, 1H), 7.07 (dd, J = 7.0 Hz, 1H). HRMS (EI) *m/z*: calcd for $C_{21}H_{14}N_4O$: 338.1168, found: 338.1173.

2-(methylamino)-4-phenyl-6-(thiophen-3-yl)pyrimidine-5-carbonitrile (19az). Yield: 58%. Mp 160-162ºC. ^{1}H NMR (300 MHz, $CDCl_3$), δ (ppm): 8.62-8.34 (m, 1H), 8.06-7.77 (m, 3H), 7.61-7.45 (m, 3H), 7.42 (dd, J = 4.8, 2.9 Hz, 1H), 5.70 (brs, 1H), 3.14 (d, J = 5.1 Hz, 3H). HRMS (EI) *m/z*: calcd for $C_{16}H_{12}N_4S$: 292.0783, found: 292.0787.

4-phenyl-2-(phenylamino)-6-(thiophen-3-yl)pyrimidine-5-carbonitrile (19ba). Yield: 72%. Mp 160-162ºC. ^{1}H NMR (300 MHz, $CDCl_3$), δ (ppm): 8.56 (s, 1H), 8.00-7.94 (m, 2H), 7.74-7.67 (m, 3H), 7.59-7.51 (m, 4H), 7.47-7.36 (m, 3H), 7.19-7.11 (m, 1H). HRMS (EI) *m/z*: calcd for $C_{21}H_{14}N_4S$: 354.0939, found: 354.0941.

2-(methylamino)-4-phenyl-6-(pyridin-4-yl)pyrimidine-5-carbonitrile (19bb). Yield: 74%. Mp 256-258ºC. ^{1}H NMR (300 MHz, DMSO-d_6), δ (ppm): 8.80 (t, J = 4.3 Hz, 2H), 8.47 (s, 1H), 8.05-7.72 (m, 4H), 7.68-7.49 (m, 3H), 2.96 (brs, 3H). HRMS (EI) *m/z*: calcd for $C_{17}H_{13}N_5$: 287.1171, found: 287.1175.

4-phenyl-2-(phenylamino)-6-(pyridin-4-yl)pyrimidine-5-carbonitrile (19bc). Yield: 66%. Mp 307-308ºC. ^{1}H NMR (300 MHz, DMSO-d_6), δ (ppm): 10.71 (s, 1H), 8.88-8.79 (m, 2H), 8.02-7.93 (m, 1H), 7.88 (d, J = 5.9 Hz, 2H), 7.79 (d, J = 7.7 Hz, 2H), 7.67-7.53 (m, 4H), 7.34 (dd, J = 7.7 Hz, 2H), 7.07 (dd, J = 7.4 Hz, 1H). HRMS (EI) *m/z*: calcd for $C_{22}H_{15}N_5$: 349.1327, found: 349.1312.

2-(methylamino)-4-phenyl-6-(pyridin-3-yl)pyrimidine-5-carbonitrile (19bd). Yield: 43%. Mp 248-249 °C. ^{1}H NMR (300 MHz, DMSO-d_6), δ (ppm): 9.05 (brs, 1H), 8.78-8.74 (m, 1H), 8.44 (m, 1H), 8.34-8.23 (m, 1H), 7.97-7.85 (m, 2H), 7.62-7.54 (m, 4H), 2.96 (d, J = 4.7 Hz, 3H).^{13}C NMR (75 MHz, DMSO-d_6) δ (ppm): 187.9, 170.3, 168.9, 161.6, 151.7, 149.4, 136.4, 132.7, 131.2, 128.9, 123.5, 118.6, 112.3, 91.3, 28.0. HRMS (ESI) *m/z*: calcd for: $C_{17}H_{13}N_5$ $[M+H]^+$ 288.1249, found: 288.1244.

2-(ethylamino)-4-phenyl-6-(pyridin-3-yl)pyrimidine-5-carbonitrile (19be). Yield: 48%. Mp 152-153ºC. ^{1}H NMR (300 MHz, $CDCl_3$), δ (ppm): 8.58-8.49 (m, 1H), 7.98-7.80 (m, 4H), 7.70-7.52 (m, 5H), 3.50-3.38 (m, 2H), 1.17 (t, J = 7.1 Hz, 3H).^{13}C NMR (75 MHz, DMSO-d_6) δ (ppm): 169.8, 136.8, 131.1, 129.0, 128.5, 123.5, 107.0, 93.1, 36.9. MS (EI) *m/z*: 301.0 (M^+, 91), 300.0 (100), 286.0 (48), 273.0 (19), 272.0 (78), 258.0 (21), 129.0 (21), 77.0 (16).

4-phenyl-2-(phenylamino)-6-(pyridin-3-yl)pyrimidine-5-carbonitrile (19bf). Yield: 42%. Mp 245-246ºC. ^{1}H NMR (300 MHz, DMSO-d_6), δ (ppm): 10.68 (s, 1H), 9.14 (d, J = 2.0 Hz, 1H), 8.85-8.76 (m, 1H), 8.37 (dt, J = 8.1, 1.9 Hz, 1H), 8.07-7.94 (m, 2H), 7.81 (d, J = 8.6 Hz, 2H), 7.68-7.58 (m, 4H), 7.36 (dd, J = 8.0 Hz, 2H), 7.08 (dd, J = 7.4 Hz, 1H). HRMS (EI) *m/z*: calcd for $C_{22}H_{15}N_5$ 349.1327, found: 349.1328.

4-cyclohexyl-2-(methylamino)-6-phenylpyrimidine-5-carbonitrile (19bg). Yield: 51%. Mp 204-205ºC. ^{1}H NMR (300 MHz, $CDCl_3$), δ (ppm): 7.99-7.92 (m, 1H), 7.85-7.78 (m, 1H), 7.49-7.24 (m, 3H), 5.67-5.53 (m, 1H), 3.09-3.04 (m, 4H), 1.82-1.23 (m, 10H). HRMS (EI) *m/z*: calcd for $C_{18}H_{20}N_4$: 292.1610, found: 292.1688.

4-cyclohexyl-6-phenyl-2-(phenylamino)pyrimidine-5-carbonitrile (19bh). Yield: 67%. Mp 118-120ºC. ^{1}H NMR (300 MHz, $CDCl_3$), δ (ppm): 7.59 (d, J = 6.70 Hz, 2H), 7.39-7.32 (m, 6H), 7.18 (t, J = 7.44 Hz, 1H), 6.99 (d, J = 7.55 Hz, 2H), 3.92 (s, 1H), 1.86-0.85 (m, 10H). MS (EI) *m/z*: 354.17 (M^+, 22), 353.15 (17), 274.1 (50), 273.1 (100).

4,6-bis(3-chlorophenyl)-2-(methylamino)pyrimidine-5-carbonitrile (19bi). Yield: 42%. Mp 255-257ºC. ^{1}H NMR (300 MHz, DMSO-d_6), δ (ppm): 8.50 (q, J = 4.2 Hz, 1H), 7.99 (t, J = 1.9Hz, 1H), 7.94-7.88 (m, 2H), 7.84 (dt, J = 7.3, 1.5 Hz, 1H), 7.72-7.54 (m, 4H), 2.95 (d, J = 4.8 Hz, 3H). HRMS (EI) *m/z*: calcd for $C_{18}H_{12}Cl_2N_4$: 354.0439, found: 354.0434.

4,6-bis(3-chlorophenyl)-2-(phenylamino)pyrimidine-5-carbonitrile (19bj). Yield: 52%. Mp 211-213ºC. ^{1}H NMR (300 MHz, DMSO-d_6), δ (ppm): 10.71 (s, 1H), 8.03 (s, 1H), 7.94 (d, J = 7.2

Hz, 2H), 7.83-7.60 (m, 7H), 7.35 (dd, *J* = 7.9 Hz, 2H), 7.08 (dd, *J* = 7.9 Hz, 1H). MS (EI) *m/z*: 420.1 (36), 418.1 (72), 417.1 (83), 416.1 (M^+, 100), 415.1 (88), 381.0 (13).

4-(3-chlorophenyl)-6-(3,5-dichlorophenyl)-2-(methylamino)pyrimidine-5-carbonitrile (19bk). Yield: 72%. Mp 255-257ºC. ^{1}H NMR (300 MHz, DMSO-d_6), δ (ppm): 8.56 (q, *J* = 4.9 Hz, 1H), 8.04-7.79 (m, 5H), 7.74-7.56 (m, 2H), 2.95 (d, *J* = 4.5 Hz, 3H). HRMS (EI) *m/z*: calcd for $C_{18}H_{11}Cl_3N_4$: 388.0049, found: 388.0048.

4-(3-chlorophenyl)-6-(3,5-dichlorophenyl)-2-(phenylamino)pyrimidine-5-carbonitrile (19bl). Yield: 56%. Mp 199-201ºC. ^{1}H NMR (300 MHz, DMSO-d_6), δ (ppm): 8.13-8.05 (m, 2H), 7.88-7.82 (m, 2H), 7.68 (d, *J* = 8.7Hz, 2H), 7.59-7.54 (m, 3H), 7.42-7.37 (m, 2H), 7,16 (t, *J* = 8.4Hz, 1H). MS (EI) *m/z*: 456.2 (10), 454.2 (40) 452.2 (96), 450.2 (M^+, 100), 415.2 (44), 386.2 (28).

4,6-bis(4-methoxyphenyl)-2-(methylamino)pyrimidine-5-carbonitrile (19bm). Yield: 70%. Mp 249-250ºC. ^{1}H NMR (300 MHz, $CDCl_3$), δ (ppm): 8.08 (d, *J* = 7.2 Hz, 2H), 7.93 (d, *J* = 7.2 Hz, 2H), 7.03 (d, *J* = 7.2 Hz, 4H), 5.64 (s, 1H), 3.88 (s, 6H), 3.15 (d, *J* = 5.0 Hz, 3H). HRMS (EI) *m/z*: calcd for $C_{20}H_{18}N_4O_2$: 346.1430, found: 346.1435.

4,6-bis(4-methoxyphenyl)-2-(phenylamino)pyrimidine-5-carbonitrile (19bn). Yield: 48%. Mp 210-211°C. ^{1}H NMR (300 MHz, $CDCl_3$), δ (ppm): 10.50 (bs, 1H), 7.97 (d, *J* =8.7 Hz, 4H), 7.82 (d, *J* = 7.6 Hz, 2H), 7.33 (m, 2H), 7.13 (d, *J* =8.7 Hz, 4H), 7.05 (t, *J* = 7.6 Hz, 1H), 3.85 (s, 6H).^{13}C NMR (75 MHz, $CDCl_3$) δ (ppm): 169.7, 161.8, 159.0, 139.3, 131.0, 128.8, 123.1, 120.3, 119.0, 114.0, 91.8, 55.6. HRMS (ESI) *m/z*: calcd for $C_{25}H_{20}N_4O_2$ $[M+H]^+$: 409.1665, found: 409.1659.

2-(dimethylamino)-4,6-diphenylpyrimidine-5-carbonitrile (20a). Yield: 46%. Mp 233-234ºC. ^{1}H NMR (300 MHz, DMSO-d_6), δ (ppm): 7.99-7.86 (m, 4H), 7.60-7.55 (m, 6H), 3.29 (s, 6H). ^{13}C NMR (75 MHz, DMSO-d_6) δ (ppm): 169.8, 136.8, 131.1, 129.0, 128.5, 123.5, 107.0, 93.1, 36.9. HRMS (ESI) *m/z*: calcd for $C_{19}H_{16}N_4$ $[M+H]^+$: 301.1453, found: 301.1448.

4-(3-chlorophenyl)-2-(dimethylamino)-6-phenylpyrimidine-5-carbonitrile (20b). Yield: 42%. Mp 178-179ºC. ^{1}H NMR (300 MHz, DMSO-d_6), δ (ppm): 7.94-7.86 (m, 4H), 7.66-7.52 (m, 5H), 3.27 (s, 6H). ^{13}C NMR (75 MHz, DMSO-d_6) δ (ppm): 170.1, 168.7, 160.5, 139.1, 137.0, 133.6, 131.5, 131.2, 130.8, 129.3, 129.0, 128.9, 128.0, 119.0, 90.3, 37.3. HRMS (ESI) *m/z*: calcd for $C_{19}H_{15}ClN_4$ $[M+H]^+$: 335.0985, found: 335.0991.

2-(dimethylamino)-4-(4-fluorophenyl)-6-phenylpyrimidine-5-carbonitrile (20c). Yield: 73%. Mp 237-238ºC. ^{1}H NMR (300 MHz, DMSO-d_6), δ (ppm): 8.04-8.01 (m, 2H), 7.99-7.91 (m, 2H), 7.57-7.55 (m, 3H), 7.43-7.37 (m, 2H), 2.48 (s, 3H), 2.47 (s, 3H). ^{13}C NMR (75 MHz, DMSO-d_6) δ (ppm): 170.2, 169.7, 160.6, 137.1, 133.6, 132.0, 131.5, 129.3, 128.9, 119.2, 116.1, 115.8, 90.3, 37.3. HRMS (ESI) *m/z*: calcd for $C_{19}H_{15}N_4F$ $[M+H]^+$: 319.1359, found: 319.1354.

Supplementary Figures

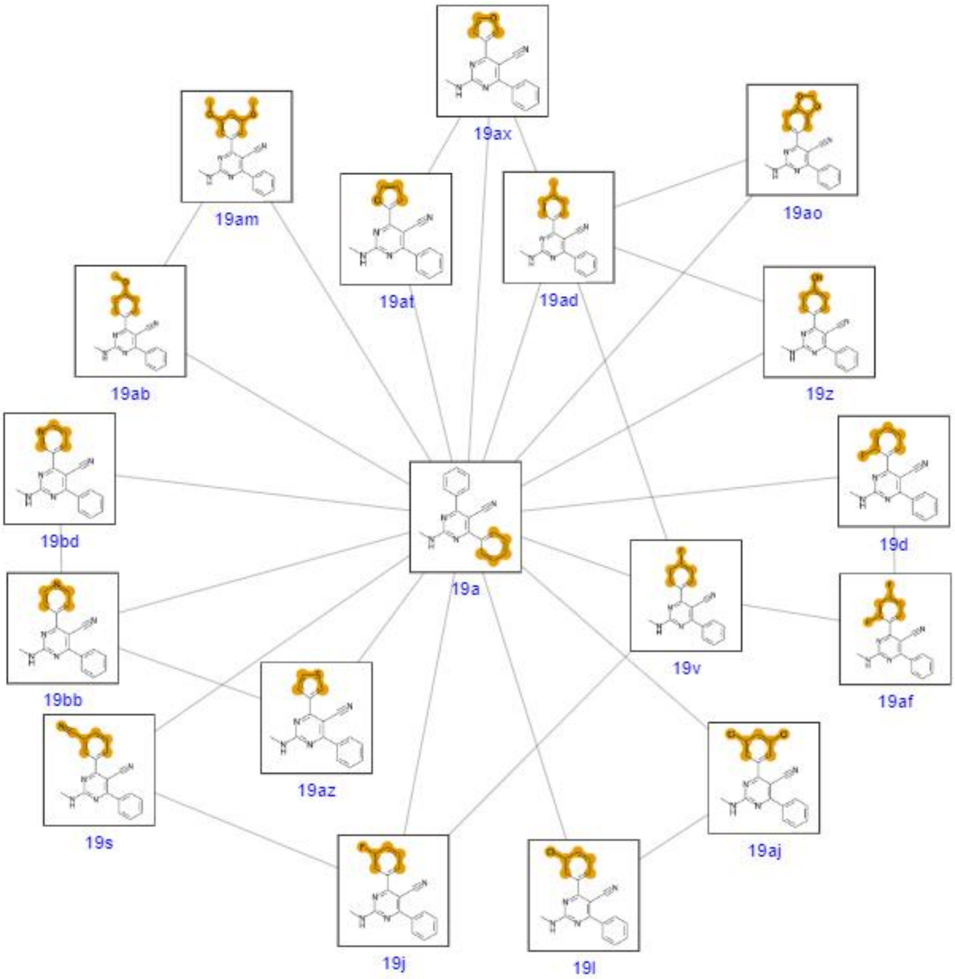

Supplementary Figure 1. FEP pathway used along this study: The 18 compounds (nodes) were characterized with a total of and 29 alchemical transformations (edges) between selected compound pairs. An ulterior cycle closure correction allows the conversion of the 29 calculated $\Delta\Delta G_{\text{binding}}$ between compound pairs, into estimates of the absolute binding affinity (ΔG_{bind}) for each compound.

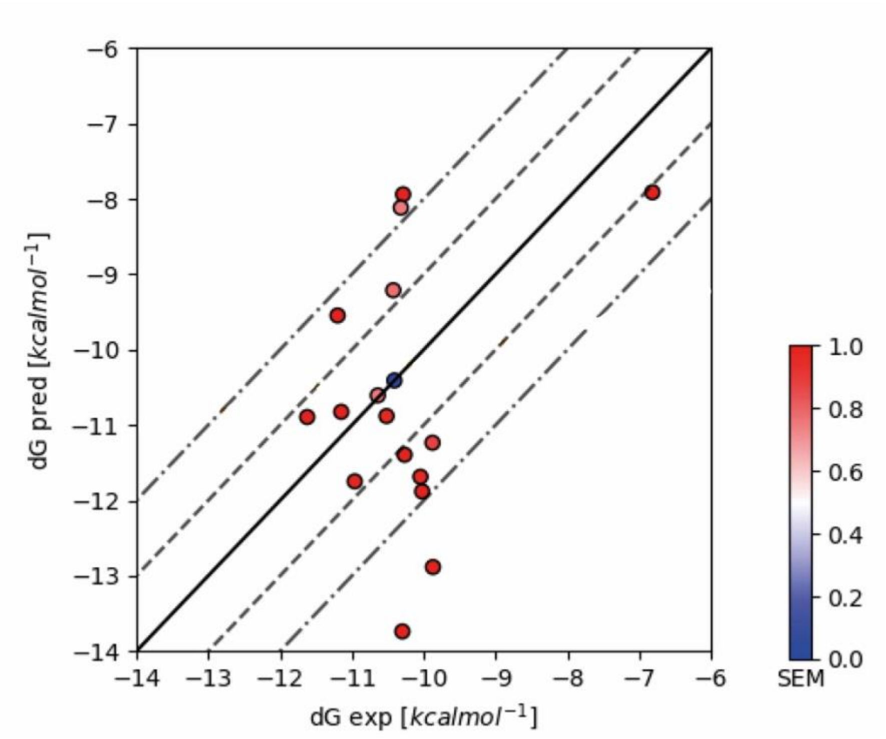

Supplementary Figure 2. Scatter plot of the predicted vs experimental binding affinities for the A_1AR, as determined by FEP calculations using conformation B. Statistical data: MAE = 1.68 ± 0.39, RMSE = 2.26 ± 0.59, SEM 1.12 ± 0.1. Confidence intervals for the regression metrics were estimated using bootstrap sampling.

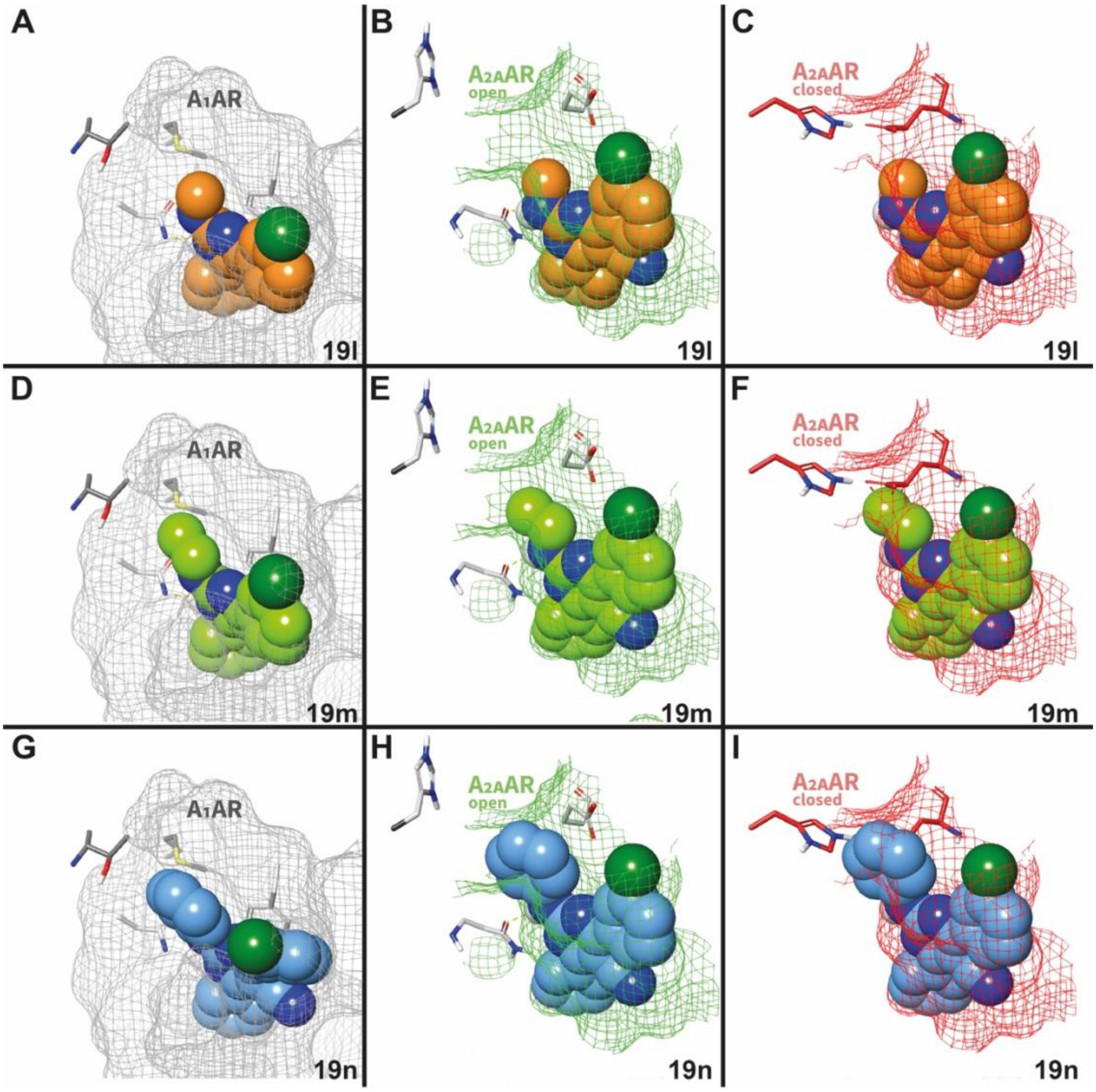

Supplementary Figure 3. Surface and balls representation for ligands N-substituted. A, B and C) 19l binding A_1AR, $A_{2A}AR$ (open, rotamers from PDB: 3uzc) and $A_{2A}AR$ (closed, PDB: 4eiy) respectively. N-methylation allows efficient binding in A_1AR but difficult binding in $A_{2A}AR$ closed, not stabilizing $A_{2A}AR$ open. D, E and F) 19m binding A_1AR, $A_{2A}AR$ (open, rotamers from PDB: 3uzc) and $A_{2A}AR$ (closed, PDB: 4eiy) respectively. N-ethylation allows binding in A_1AR but difficult binding in $A_{2A}AR$ closed, slightly stabilizing $A_{2A}AR$ open. G, H and I) 19n binding A_1AR, $A_{2A}AR$ (open, rotamers from PDB: 3uzc) and $A_{2A}AR$ (closed, PDB: 4eiy) respectively. N-phenylation causes a loss of a hydrogen bond by rearrangement of the molecule in A_1AR but strongly stabilizes $A_{2A}AR$ open.

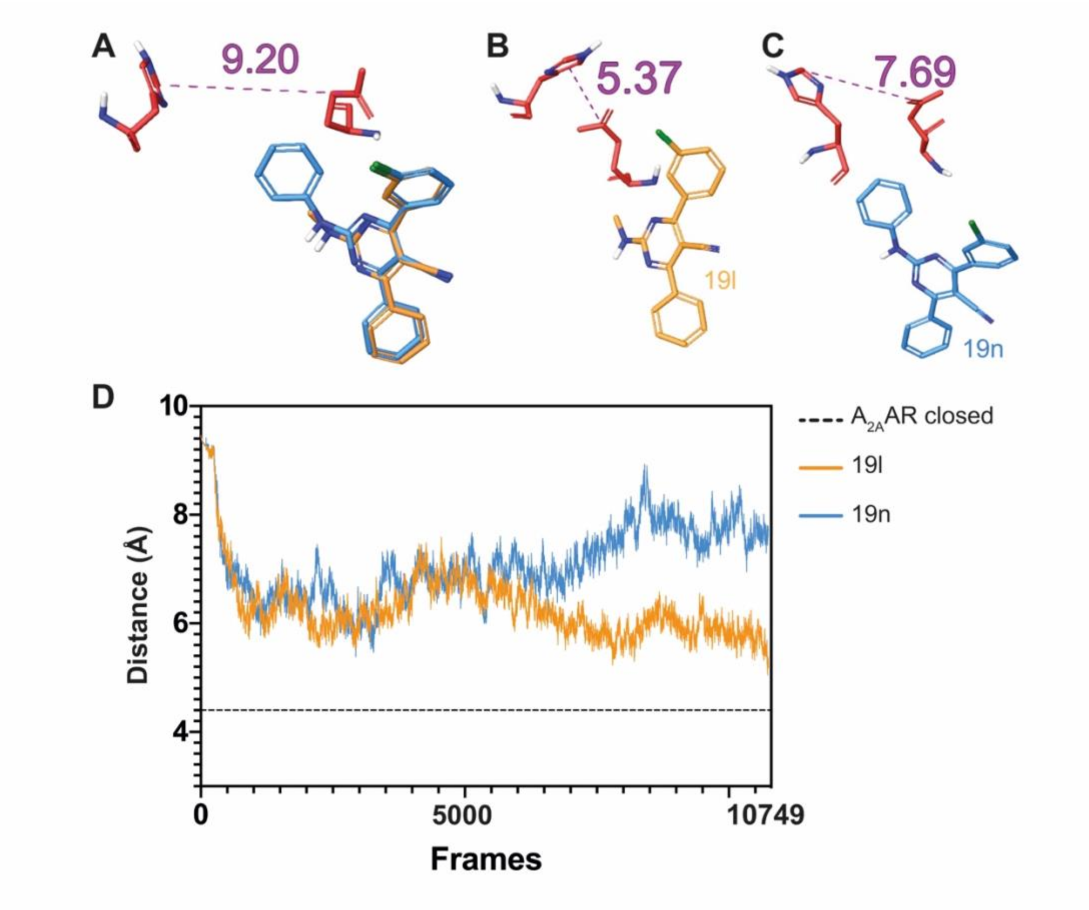

Supplementary Figure 4. A. Initial state at A_{2A}AR conformation in MD simulations. Distance between C-His264^{EL3} and CO-Glu169^{EL2} in Å. **B.** Final state conformation of 19l (*N*-methyl compound) at A_{2A}AR. **C**. Final state conformation of 19n (*N*-phenyl compound) at A_{2A}AR. **D.** Distance between C-His264^{EL3} and CO-Glu169^{EL2} in Å in MD simulations. 19l (orange) does not able to keep the A_{2A}AR open, but 19n (blue) stabilized the open A_{2A}AR open conformation.

Supplementary Tables

The functional profile of selected ligands (**19ao, 19l** and **19v**) was evaluated in cAMP assays following experimental procedures previously reported by us.[2,3,4]

Supplementary Table 1. Functional studies of **19ao**, **19l**, **19v (**at the four adenosine receptors).

	***h*A$_1$**	***h*A$_{2A}$**[2]	***h*A$_{2B}$**[3]	***h*A$_3$**[4]
**Compound**	**Kb (nM)**	**% Inhib. NECA (1 µM)**	**% Inhib. NECA (1 µM)**	**% Inhib. NECA (1 µM)**
**19l**	6.21	16%	11%	9%
**19v**	9.72	25%	1%	5%
**19ao**	3.90	18%	1%	5%

Human Microsomal Stability. The human microsomes employed were purchased from Tebu-Xenotech. The compound was incubated with microsomes at 37 °C in a 50 mM phosphate buffer (pH = 7.4) containing 30 mM $MgCl_2$, 10 mM NADP, 100 mM glucose-6-phosphate, and 40 U/mL glucose-6-phosphate dehydrogenase. Samples (75 µL) were taken from each well at 0, 10, 20, 40, and 60 min and transferred to a plate containing 75 µL of acetonitrile (4 °C), and 30 µL of 0.5% formic acid in water was added for improving the chromatographic conditions. The plate was centrifuged (4000g, 60 min) and supernatants were taken and analyzed in a UPLC-MS/MS (Xevo-TQD, Waters) by employing a BEH C18 column and an isocratic gradient of 0.1% formic acid in water: 0.1% formic acid acetonitrile (60:40). The metabolic stability of the compounds was calculated from the logarithm of the remaining compounds at each of the time points studied.

Supplementary Table 2. Stability parameters obtained for each of the studied compounds.

		**Human**	
**Compound**	**% Remanent (sampling time 60 min)**	**$t_{1/2}$ (min)**	**CL_{int} ($\mu L \cdot mg_{protein}^{-1} \cdot min^{-1}$)**
**19l**	3.7	6.24	27.78
**19v**	15.1	18.65	32.41
**19ao**	11.4	15.24	50.28
**Testosterone**	8.77	17.09	27.78

Supplementary Table 3. pKi of compounds selected for docking studies. The selection criteria were based on the primary structure of the novel compounds. Taking the compounds that show affinity data (Ki), the pKi $[-log\ (K_i)]$ was calculated for both receptors and with all the R^2 substitutions (H, Me, Et, Ph) analogues. With this, ΔpKi was calculated as $\Delta pKi_{receptor} = pK_i\ (R_2 = H)_{receptor} - pK_i\ (R_2 \neq H)_{receptor}$ for A_1AR and $A_{2A}AR$. With this data, ΔΔpKi was calculated as $\Delta\Delta pKi_{R_2} = \Delta pKi_{A_{2A}} - \Delta pKi_{A_1}$. Pairs of compounds showing more difference between the H and the R^2 (the largest absolute values of ΔΔpKi) was selected with the same scaffold compounds for docking studies (Graphic below). N-methylated compounds with this scaffold was selected for FEP studies.

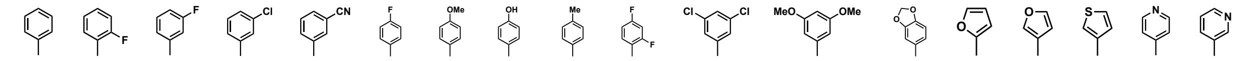

R^2 / R^4																			
H	A_1	8.4	8.2	7.9	8.4	8.2	8.4	8.1	8.4	8.3	8.4	7.8	8.6	8.8	8.0	7.7	8.3	6.7	7.7
	A_{2A}	8.1	7.8	7.7	7.8	7.1	7.8	7.3	7.6	8.0	7.8	7.0	8.8	8.0	8.0	7.3	7.7	8.2	8.1
Me	A_1	8.0	7.5	7.5	7.8	8.5	7.6	7.2	7.4	7.6	7.6	7.6	8.0	8.2	8.2	5.0	7.4	7.2	7.7
	A_{2A}	6.1	5.0	5.0	5.0	7.1	5.0	5.0	5.0	5.0	5.0	5.0	7.9	5.0	6.4	5.0	5.0	5.0	5.0
Et	A_1	8.2	-	-	8.3	8.6	-	-	-	-	-	6.9	-	8.2	-	-	-	-	7.9
	A_{2A}	6.4	-	-	6.5	6.8	-	-	-	-	-	5.0	-	6.0	-	-	-	-	8.1
Ph	A_1	7.3	8.2	5.0	5.0	7.7	7.4	5.0	5.0	7.4	5.0	5.0	5.0	7.9	7.5	5.0	5.0	5.0	5.0
	A_{2A}	7.3	7.3	5.0	8.3	5.0	5.0	7.3	5.0	6.8	5.0	5.0	7.8	7.6	8.7	6.4	7.0	5.0	8.3

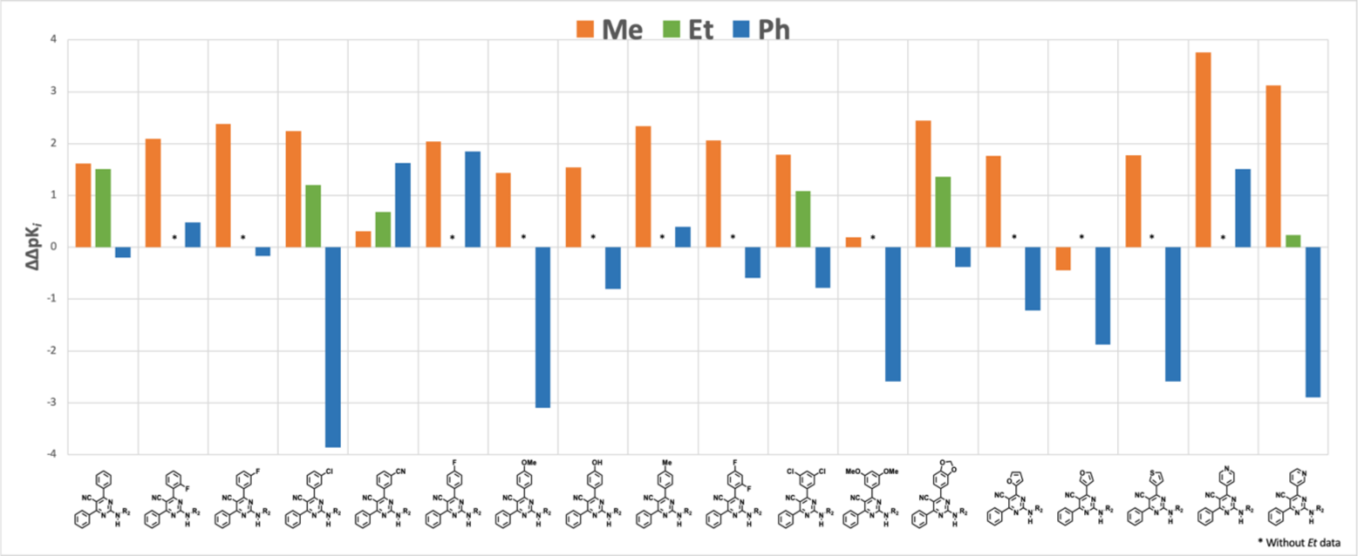

Supplementary Table 4. Calculated and experimental relative binding affinity ($\Delta\Delta G_{bind}$) between the pairs of compounds defined in the FEP pathway defined in Supplementary Figure 2. Errors are defined as standard error of the mean (sem) from 10 independent simulations. UE indicates the unnasigned error as compared to experiment (Exp) on each conformation modelled (A or B)

**Lig 1**	**Lig 2**	**Exp**	**Conformation A**		**Conformation B**	
		$\Delta\Delta G_{bind}$	$\Delta\Delta G_{bind}$	UE	$\Delta\Delta G_{bind}$	UE
**19a**	**19ab**	1,09	0.83 ± 0.45	0.26	-1.14 ± 1.01	2,23
**19a**	**19aj**	0,64	-2.54 ± 0.16	3.18	3,63 ± 0,09	2,99
**19a**	**19am**	0,11	-1.98 ± 0,57	2,09	8,09 ± 1,01	7,98
**19a**	**19ao**	-0,24	-0.55 ± 0.36	0.31	2.20 ± 1.25	2.44
**19a**	**19at**	-0.19	0.49 ± 0.25	0.68	0.92 ± 1.25	1.11
**19a**	**19ax**	4.14	0.94 ± 0.18	3.20	3.83 ± 1.14	0.31
**19a**	**19az**	0.91	-1.28 ± 0.17	2.19	0.06 ± 0.39	0.85
**19a**	**19bb**	1.08	-0.16 ± 0.18	1.24	0.51 ± 0.27	0.57
**19a**	**19bd**	0.44	-0.30 ± 0.13	0.74	0.87 ± 0.39	0.42
**19a**	**19d**	0.69	0.20 ± 0.15	0.49	0.35 ± 0.78	0.34
**19a**	**19j**	0.67	0.01 ± 0.21	0.66	3.81 ± 0.87	3.13
**19a**	**19l**	0.32	-0.57 ± 0.16	0.89	1.14 ± 0.09	0.82
**19a**	**19s**	-0.66	-1.39 ± 0.43	0.73	0.85 ± 1.17	1.52
**19a**	**19v**	0.55	0.95 ± 0.13	0.40	1.34 ± 0.98	0.79
**19a**	**19z**	0.94	0.93 ± 0.21	0.01	-0.14 ± 0.87	1.07
**19ab**	**19am**	-0.98	-3.20 ± 0.62	2.22	9.22 ± 1.01	10.20
**19ad**	**19a**	-0.66	-0.37 ± 0.18	0.29	1.99 ± 1.05	2.65
**19ad**	**19ao**	-0.90	-0.26 ± 0.35	0.64	4.19 ± 1.25	5.09
**19ad**	**19ax**	3.48	1.02 ± 0.46	2.46	5.83 ± 1.25	2.34
**19ad**	**19v**	-0.11	1.18 ± 0.33	1.29	3.33 ± 1.17	3.44
**19ad**	**19z**	0.28	0.68 ± 0.23	0.40	1.86 ± 0.87	1.58
**19aj**	**19l**	-0.32	1.06 ± 0.31	1.38	-2.49 ± 0.09	2.17
**19ax**	**19at**	-4.33	-0.98 ± 0.17	3.35	-2.91 ± 1.25	1.42
**19bb**	**19az**	-0.17	-0.99 ± 0.17	0.82	-0.45 ± 0.39	0.28
**19bb**	**19bd**	-0.64	0.15 ± 0.16	0.79	0.35 ± 0.39	0.99
**19d**	**19af**	-0.16	1.51 ± 0.16	1.67	2.18 ± 0.78	2.34
**19s**	**19j**	1.34	1.14 ± 0.37	0.20	2.95 ± 1.17	1.62
**19v**	**19af**	-0.02	0.27 ± 0.23	0.29	1.20 ± 0.78	1.21
**19v**	**19j**	0.12	-1.28 ± 0.28	1.40	2.47 ± 1.17	2.35
			**MUE**	1.18	**MUE**	2.22

Supplementary Table 5. Calculated and experimental absolite binding affinity (ΔG_{bind}) for each compound within the FEP dataset (the data is plotted in Figure 6B and Supplementary Figure 1). Errors are defined by the cycle closure correction (CCC) estimated from the FEP pathway shown in Supplementary Figure 2, with the reference compound indicated (REF).

**Lig**	**Exp**	**Conformation A**		**Conformation B**	
	ΔG_{bind}	ΔG_{bind}	UE	ΔG_{bind}	UE
**19a**	-10.96	-10.31 ± 0.23	0.65	-11.75 ± 0.98	0.79
**19ab**	-9.87	-9.35 ± 0.32	0.52	-12.88 ± 1.40	3.01
**19ad**	-10.30	-10.20 ± 0.43	0.10	-13.74 ± 1.17	3.44
**19af**	-10.42	-8.78 ± 0.42	1.64	-9.21 ± 0.78	1.21
**19aj**	-10.32	-12.55 ± 0.54	2.23	-8.12 ± 0.75	2.20
**19am**	-10.85	-12.42 ± 0.32	1.57	-3.66 ± 1.40	7.19
**19ao**	-11.20	-10.66 ± 0.43	0.54	-9.55 ± 1.57	1.65
**19at**	-11.15	-10.00 ± 0.40	1.15	-10.83 ± 1.57	0.32
**19ax**	-6.82	-9.19 ± 0.38	2.37	-7.91 ± 1.71	1.10
**19az**	-10.05	-11.58 ± 0.22	1.54	-11.69 ± 0.96	1.64
**19bb**	-9.88	-10.58 ± 0.28	0.70	-11.24 ± 0.88	1.36
**19bd**	-10.52	-10.52 ± 0.28	0.00	-10.88 ± 0.96	0.36
**19d**	-10.27	-10.20 ± 0.34	0.06	-11.39 ± 1.24	1.13
**19j**	-10.29	-10.40 ± 0.23	0.11	-7.94 ± 1.17	2.35
**19l**	-10.64	-11.18 ± 0.54	0.54	-10.61 ± 0.75	0.03
**19s**	-11.62	-11.62	(REF)	-10.89 ± 1.65	0.73
**19v**	-10.41	-9.14 ± 0.329	1.27	-10.41	(REF)
**19z**	-10.02	-9.45 ± 0.356	0.57	-11.88 ± 1.30	1.86
		MUE	0.92	MUE	1.77

HPLC traces for lead compounds

Project Name Rubén Prieto
Reported by User: Breeze user (Breeze)

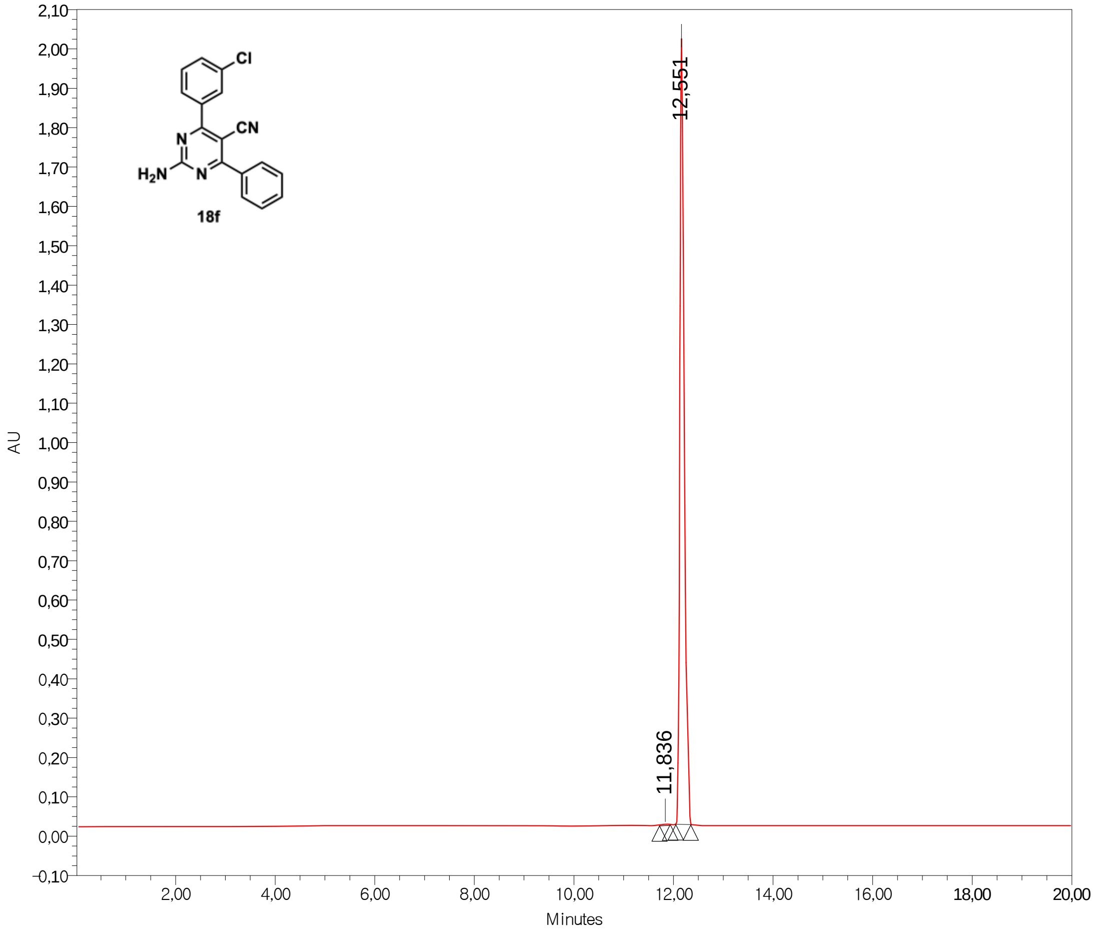

Sample Name: 18f; Date Acquired: 25/02/2021 11:15:52 CET; Vial: 1; Injection: 1

Peak Summary with Statistics
Peak Name:

	Sample Name	Vial	Inj.	RT (min)	Area (µV*sec)	% Area	Height (µV)
1	18f	1	1	12,551	24163640,67	99,50	4004378
2	18f	1	1	11,836	121425,33	0,50	212118
Mean				12,194	12142533		2012769
Std. Dev.				505,5	17000413,5		2816487,63
% RSD				4,14	140,01		139,931

Report Method: Peak Summary Report
Printed: 03/09/2021 17:40:22 Europe/Madrid
Page: 1 of 2

Acquisition Log

Acquired By	Breeze
Injection	1
Date Acquired	25/02/2021 11:15:52 CET
Run Time	20,00(Minutes)
Acq Method Set	20%DCMIPAGradF1 set
Injection Volume	20,00(uL)
Injection Id	1600
Instrument Method Name	20%DCMIPAGradF1
Superseded	No

Project Name Rubén Prieto
Reported by User: Breeze user (Breeze)

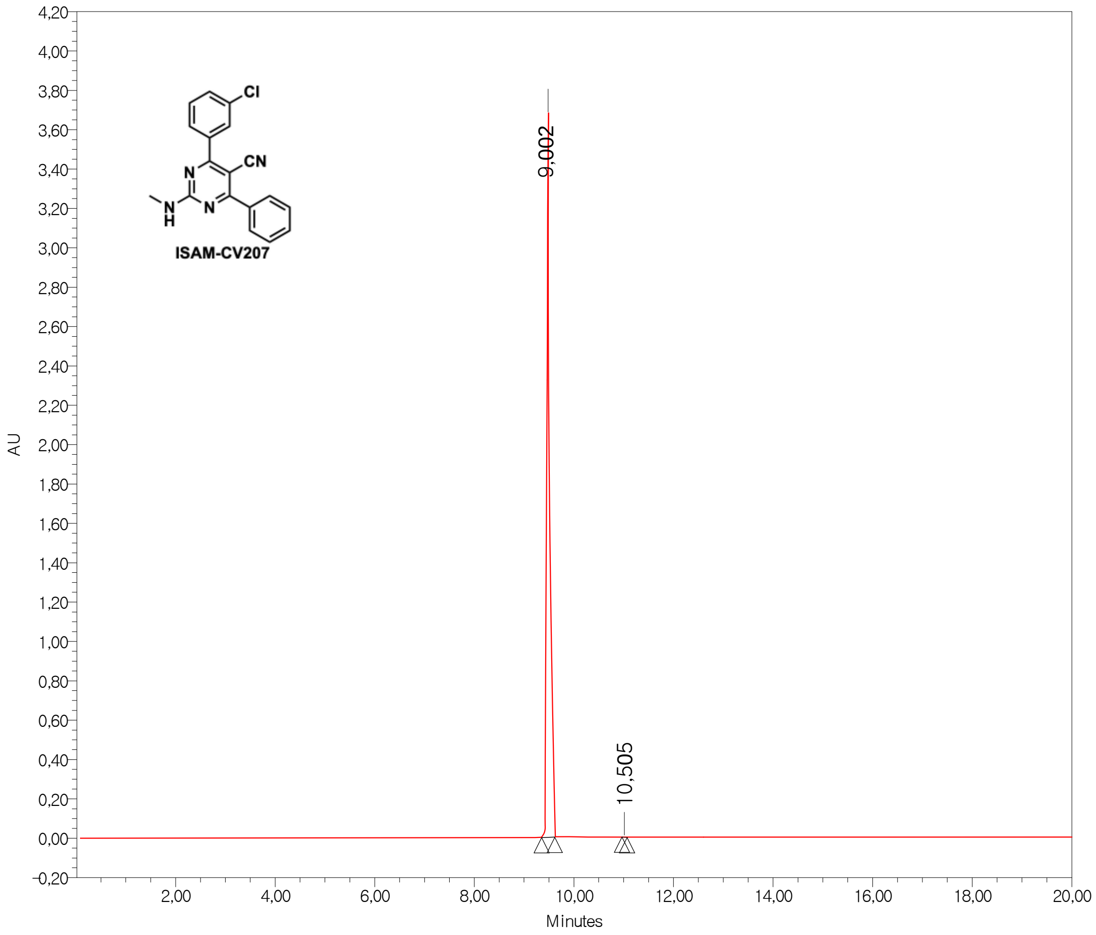

Peak Summary with Statistics
Peak Name:

	Sample Name	Vial	Inj.	RT (min)	Area (µV*sec)	% Area	Height (µV)
1	ISAM-CV207	1	1	9,002	51410697	99,98	8008756
2	ISAM-CV207	1	1	10,505	103027	0,02	2121
Mean				9,754	25756862		4005439
Std. Dev.				1,062	36280001,35		5661545,9
% RSD				10,8	140,85		141,346

Report Method: Peak Summary Report
Printed: 03/09/2021 17:42:21 Europe/Madrid
Page: 1 of 2

Acquisition Log

Acquired By	Breeze
Injection	1
Date Acquired	25/02/2021 12:16:23 CET
Run Time	20,00(Minutes)
Acq Method Set	20%DCMIPAGradF1 set
Injection Volume	20,00(uL)
Injection Id	1601
Instrument Method Name	20%DCMIPAGradF1
Superseded	No

Project Name Rubén Prieto
Reported by User: Breeze user (Breeze)

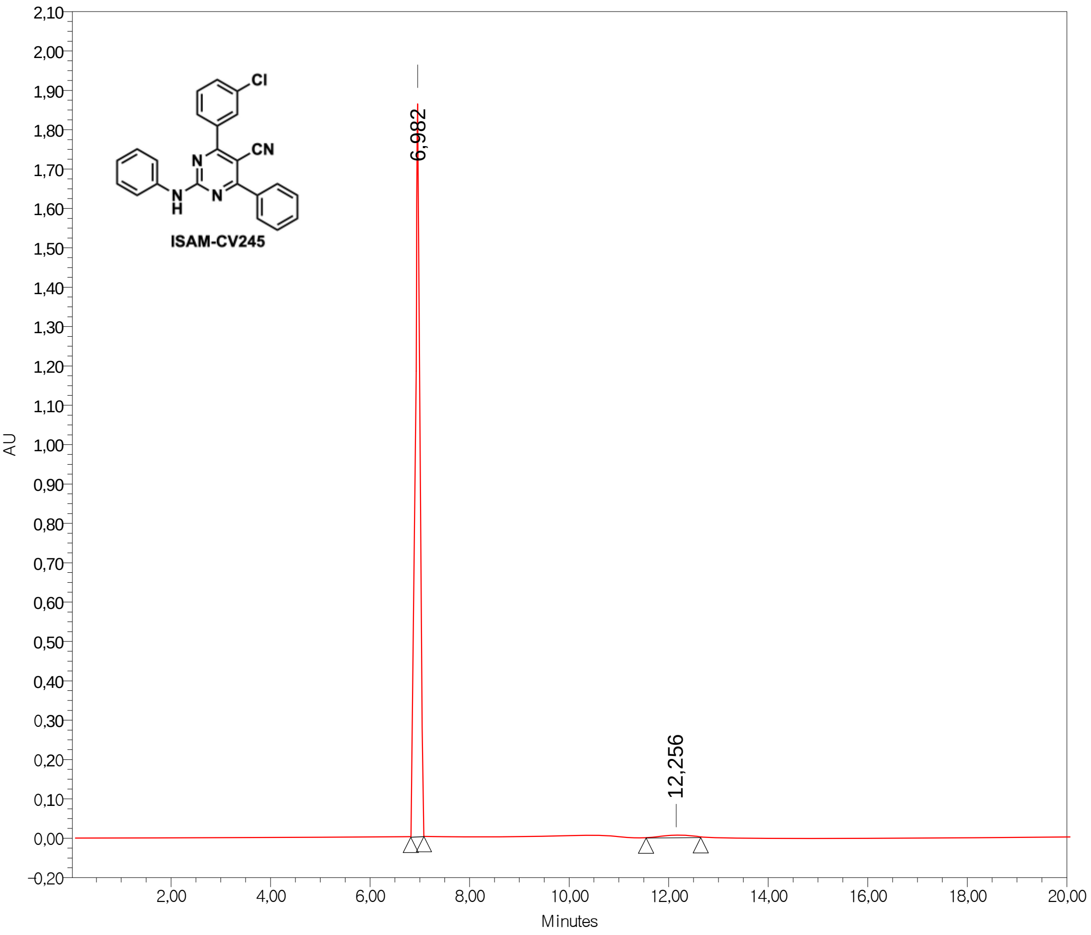

Sample Name: ISAM-CV245; Date Acquired: 25/02/2021 12:22:22 CET; Vial: 1; Injection: 1

Peak Summary with Statistics
Peak Name:

	Sample Name	Vial	Inj.	RT (min)	Area (µV*sec)	% Area	Height (µV)
1	ISAM-CV245	1	1	6,928	23354666	97,75	4005211
2	ISAM-CV245	1	1	12,256	5375754	2,25	42121
Mean				9,592	14365211		2023666
Std. Dev.				3,767	12713010,83		2802327,81
% RSD				39,3	88,50		138,477

Report Method: Peak Summary Report
Printed: 03/09/2021 18:10:22 Europe/Madrid
Page: 1 of 2

Project Name	Rubén Prieto
Reported by User:	Breeze user (Breeze)

Acquisition Log

Acquired By	Breeze
Injection	1
Date Acquired	25/02/2021 12:22:22 CET
Run Time	20,00(Minutes)
Acq Method Set	20%DCMIPAGradF1 set
Injection Volume	20,00(uL)
Injection Id	1602
Instrument Method Name	20%DCMIPAGradF1
Superseded	No

References

(1) Cheng, R. K. Y.; Segala, E.; Robertson, N.; Deflorian, F.; Doré, A. S.; Errey, J. C.; Fiez-Vandal, C.; Marshall, F. H.; Cooke, R. M. Structures of Human A_1 and A2A Adenosine Receptors with Xanthines Reveal Determinants of Selectivity. *Structure* **2017**, *25*, 1275-1285.

(2) Areias, F.; Costa, M.; Castro, M.; Brea, J.; Gregori-Puigjané, E.; Proença, M. F.; Mestres, J.; Loza, M. I. New Chromene Scaffolds for Adenosine A_{2A} Receptors: Synthesis, Pharmacology and Structure–Activity Relationships. *Eur. J. Med. Chem.* **2012**, *54*, 303–310.

(3) El Maatougui, A.; Azuaje, J.; González-Gómez, M.; Miguez, G.; Crespo, A.; Carbajales, C.; Escalante, L.; García-Mera, X.; Gutiérrez-De-Terán, H.; Sotelo, E. Discovery of Potent and Highly Selective A_{2B} Adenosine Receptor Antagonist Chemotypes. *J. Med. Chem.* **2016**, *59*, 1967–1983.

(4) Yaziji, V.; Rodríguez, D.; Gutiérrez-De-Terán, H.; Coelho, A.; Caamaño, O.; García-Mera, X.; Brea, J.; Loza, M. I.; Cadavid, M. I.; Sotelo, E. Pyrimidine Derivatives as Potent and Selective A_3 Adenosine Receptor Antagonists. *J. Med. Chem.* **2011**, *54*, 457–471.
